# Prediction of Prognosis in Patients With Endometrial Carcinoma and Immune Microenvironment Estimation Based on Ferroptosis-Related Genes

**DOI:** 10.3389/fmolb.2022.916689

**Published:** 2022-07-15

**Authors:** Shouze Liu, Qianqian Zhang, Wenhua Liu, Xianghua Huang

**Affiliations:** ^1^ Department of Gynecology, The Second Hospital of Hebei Medical University, Shijiazhuang, China; ^2^ Department of Pain, Hebei Cangzhou Hospital of Integrated Traditional Chinese and Western Medicine, Cangzhou, China

**Keywords:** ferroptosis, endometrial cancer, prognosis, signature, immune microenvironment

## Abstract

**Background:** Ferroptosis, a form of non-apoptotic cell death, has aroused worldwide interest in cancer researchers. However, the current study about the correlation between ferroptosis-related genes (FRGs) and endometrial cancer (EC) remains limited.

**Methods:** First, the transcriptome profiling and clinical data of EC patients were downloaded from The Cancer Genome Atlas (TCGA) and Clinical Proteomic Tumor Analysis Consortium (CPTAC) program as the training group and testing group, respectively. FRGs were acquired through literature mining. Then, we used R 4.1.1 software to screen the differently expressed FRGs from TCGA, which was also connected with the prognosis of EC patients. Subsequently, the risk score of each tumor sample was identified by LASSO regression analysis, and we classified these samples into the high- and low-risk groups in the light of the median risk score. Receiver operating characteristic (ROC) curve analysis and Kaplan-Meier analysis were performed to assess the accuracy of this signature. Significantly, the data from CPTAC was used to validate the prediction model externally. Furthermore, we evaluated the immune microenvironment in this model via single-sample gene set enrichment analysis (ssGSEA).

**Results:** Among the 150 FRGs, 6 differentially expressed genes (DEGs) based on TCGA had a relationship with the prognosis of EC patients, namely, TP53, AIFM2, ATG7, TLR4, PANX1 and MDM2. The survival curve indicated a higher survival probability in the low-risk group. Moreover, the FRGs-based signature acted well in the prediction of overall survival (OS). The results of external verification confirmed the prediction model we established. Finally, ssGSEA revealed significant differences in the abundance of 16 immune cells infiltration and the activity of 13 immune functions between different risk groups.

**Conclusion:** We identified a novel ferroptosis-related gene signature which could concisely predict the prognosis and immunotherapy in EC patients.

## Introduction

As endometrial cancer (EC) is the most common malignancies of the female genitals in developed countries, it is estimated that there were 417,367 new cases and 97,370 uterine corpus cancer-related deaths in 2020 (Sung et al., 2021). And the incidence continues to increase at an annual rate of 1% (Lortet-Tieulent et al., 2018). Although a large number of patients are diagnosed in the early stage, some of the early patients will suffer from recurrence. In addition, it is difficult to effectively identify these high-risk groups and apply individualized adjuvant therapies. Thus, there is an urgent need to improve the accuracy of the prognostic evaluation system for EC.

Ferroptosis, an iron-dependent non-apoptotic cell death, is caused by membrane damage mediated by excess lipid peroxidation (Stockwell et al., 2017). It is reported that ferroptosis is a vital process in varieties of cancers including EC (Mou et al., 2019; Zhang Y. Y. et al., 2021). The activation of ferroptosis can significantly enhance the cytotoxic effect of gemcitabine on pancreatic cancer (Ye et al., 2021). CircRHOT1 is involved in the malignant progression of breast cancer by attenuating ferroptosis (Zhang H. et al., 2021). *In vivo* experiments, the energy metabolism of HepG2 cells can be blocked by inducing ferroptosis (Cui et al., 2022). What’s more, ferroptosis shows enormous potential to induce cancer cells death in the treatment of gastrointestinal tumors and lung cancer (Li and Liu, 2021; Sun et al., 2021; Zhao et al., 2021). A recent study indicates that PTPN18 participated in the regulation of ferroptosis in EC cells by targeting the p-p38/Gpx4/XCT axis, thus inhibiting the proliferation of EC cells (Wang et al., 2021a). Therefore, ferroptosis seems to be a promising target for cancer therapy. There have been several reports on the correlation between ferroptosis-related genes (FRGs) and clinical prognosis in EC (Liu et al., 2021; Weijiao et al., 2021). Wang et al. identify a 13-FRGs prognostic signature for EC. Liu et al. establish a prognostic model based on 6-FRGs. In addition, Wei et al. construct a 6-FRGs contained prediction signature. However, genes in these models are widely divergent, and the relationship between ferroptosis and the prognosis of EC is still controversial. Therefore, the search for new biomarkers of ferroptosis is of great significance not only to clarify the specific mechanism of ferroptosis in EC more precisely, but also to improve the prognosis prediction for patients with EC.

In this study, we purpose to recognize the role of FRGs in EC, which can help to improve the prognosis evaluation system of EC and build a bridge between ferroptosis and immune microenvironment, expecting to provide theoretical reference for immunotherapy of EC.

## Materials and Methods

### Ferroptosis-Related Genes Data Acquisition

We acquired the quantification of gene expression and clinical data of cases from ‘TCGA-UCEC’ project in The Cancer Genome Atlas (TCGA) (https://portal.gdc.cancer.gov/projects/TCGA-UCEC), including 35 normal samples and 552 tumor samples. Then the gene expression of 99 tumor samples and the survival outcomes of corresponding patients were extracted on the basis of the Clinical Proteomic Tumor Analysis Consortium (CPTAC) database (https://proteomics.cancer.gov/programs/cptac) (Edwards et al., 2015). In addition, the clinical data must consist of survival time, vital status, stage, grade and age of patients. In this study, the tumor samples from TCGA and CPTAC represented the training group and the testing group, respectively. Finally, a total of 150 FRGs were identified based on FerrDb database (Zhou and Bao, 2020) as well as previous literature (Supplementary Table S1) (Stockwell et al., 2017; Bersuker et al., 2019; Doll et al., 2019; Hassannia et al., 2019).

### Ferroptosis-Related Genes Signature Construction

First, differentially expressed FRGs between tumor and normal samples from TCGA were sifted by the “limma” package in R 4.1.1 software following the threshold of false discovery rate (FDR) < 0.05. Then, we used the “survival” package to do univariate Cox analysis of overall survival (OS), and identified potential genes with prognostic difference (*p* < 0.05) in the training group. And the intersection of the two gene sets as mentioned above were the target FRGs with prognostic values, with which we queried the STRING website (https://cn.string-db.org/) (Szklarczyk et al., 2021) to construct functional protein association networks. Finally, we used the “glmnet” package in R to fit the least absolute shrinkage and selection operator (LASSO) regression model and classified the risk of patients in the training group and the testing group.

### Ferroptosis-Related Genes-Associated Prognostic Signature Verification

To examine the accuracy of the signature, we used “survival” package to draw Kaplan-Meier curves for the comparison of OS between high- and low-risk groups firstly, then used “timeROC” and “pheatmap” package to conduct receiver operating characteristic (ROC) curve analysis. t-Distributed Stochastic Neighbor Embedding (t-SNE) as well as Principal component analysis (PCA) was employed to reveal the dimensionality reduction of target FRGs data. Finally, the prognosis-related factors were recognized by univariate and multivariate Cox analyses from clinical information.

Additionally, we used the Human Protein Atlas (HPA) database (https://www.proteinatlas.org/) to test the core FRGs in other cancers prognosis.

### Risk-Related Differential Genes Identified and Enrichment Analysis

According to the filter criteria of FDR <0.05 as well as |log fold change (log FC)|>1, we filtered out differential FRGs between two risk cohorts using the “limma” package. Afterwards, Gene Ontology (GO) and Kyoto Encyclopedia of Genes and Genomes (KEGG) analyses were performed *via* “clusterProfiler”, “org.Hs.eg.db”, “enrichplot” and “ggplot2” packages in R.

### Immune Microenvironment Assessment

We implemented Single sample gene set enrichment analysis (ssGSEA) to visualize the activity of immune functions and the infiltration levels of immune cells in the risk prediction model in terms of the ssGSEA scores through “limma”, “ggpubr” and “reshape2” R packages. Prior to that, we involved the 16 immune cells and 13 immune functions in the calculation of ssGSEA scores.

### Drug Sensitivity

We performed drug sensitivity prediction with the “pRRophetic” package so as to investigate the therapeutic benefit of EC patients from chemotherapy.

## Results

### Target Ferroptosis-Related Genes Identification

**FIGURE 1:**
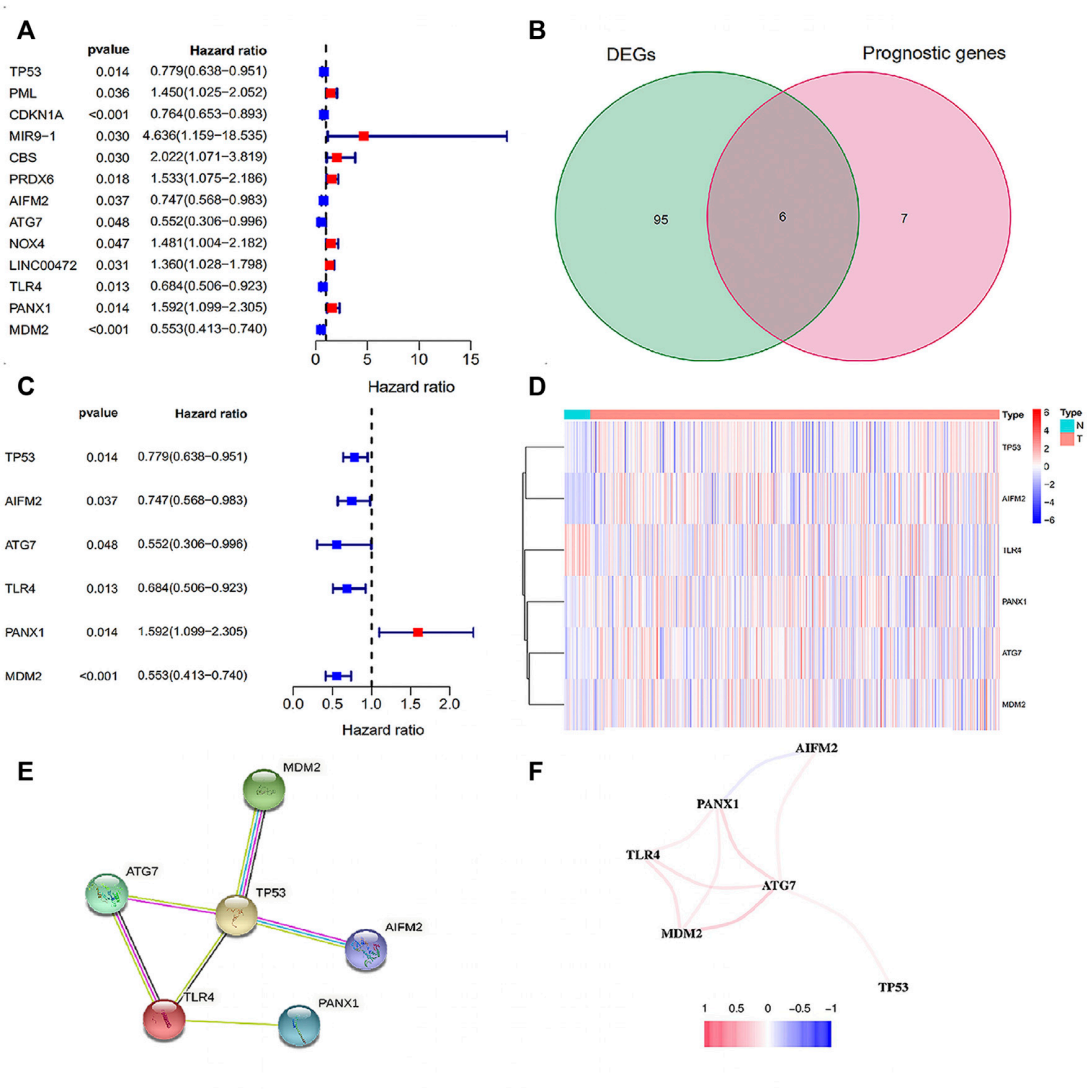
The screening of FRGs related to prognosis. **(A)** The forest plot of 13 prognosis-related FRGs; **(B)** The Venn plot of intersection FRGs. **(C)** The forest plot of intersection FRGs; **(D)** The heatmap of intersection FRGs; **(E)** The PPI network of the 6 FRGs; **(F)** The correlation network of the 6 FRGs. Red line: the positive correlation; blue line: the negative correlation.

Through wilcoxTest, 101 differentially expressed FRGS were obtained between 552 tumor samples and 35 normal samples, and 13 FRGs might significantly influence the prognosis of EC patients via univariate Cox analysis (Figure 1A). Picking the intersection of the two gene sets, we got 6 candidate FRGs (Supplementary Table S2), namely, TP53, AIFM2, ATG7, TLR4, PANX1 and MDM2 (Figure 1B). In addition, the univariate Cox analysis results revealed that TP53, AIFM2, ATG7, TLR4 and MDM2 were favorable genes, while PANX1 was the unfavorable gene (Figure 1C). In order to visualize the differences of the gene expression in the signature between normal and tumor samples, the heatmap is generated (Figure 1D). Specifically, the expression of TP53, AIFM2, PANX1, ATG7, MDM2 was up-regulated in tumor samples, while TLR4 was down-regulated (Supplementary Table S3). The details of the 6 ferroptosis-related genes were provided in Supplementary Table S4 (Hou et al., 2016; Bersuker et al., 2019; Chen et al., 2019; Doll et al., 2019; Su et al., 2019; Dai et al., 2020; Venkatesh et al., 2020; Zhang et al., 2020; Lei et al., 2021; Zhang Y. et al., 2021; Zhu et al., 2021).

### The Construction for Protein-Protein Interactions and Correlation Network

The PPI results based on the STRING database was disclosed in Figure 1E. And as shown in the correlation network, we observed a positive correlation of coexpression among ATG7, TLR4, MDM2, AIFM2 and TP53, and a negative correlation between PANX1 and AIFM2 (Figure 1F). In brief, both of the two networks confirmed the close relationship among the six candidate genes.

### Ferroptosis-Related Genes-Associated Prognostic Model Construction and Verification

**FIGURE 2:**
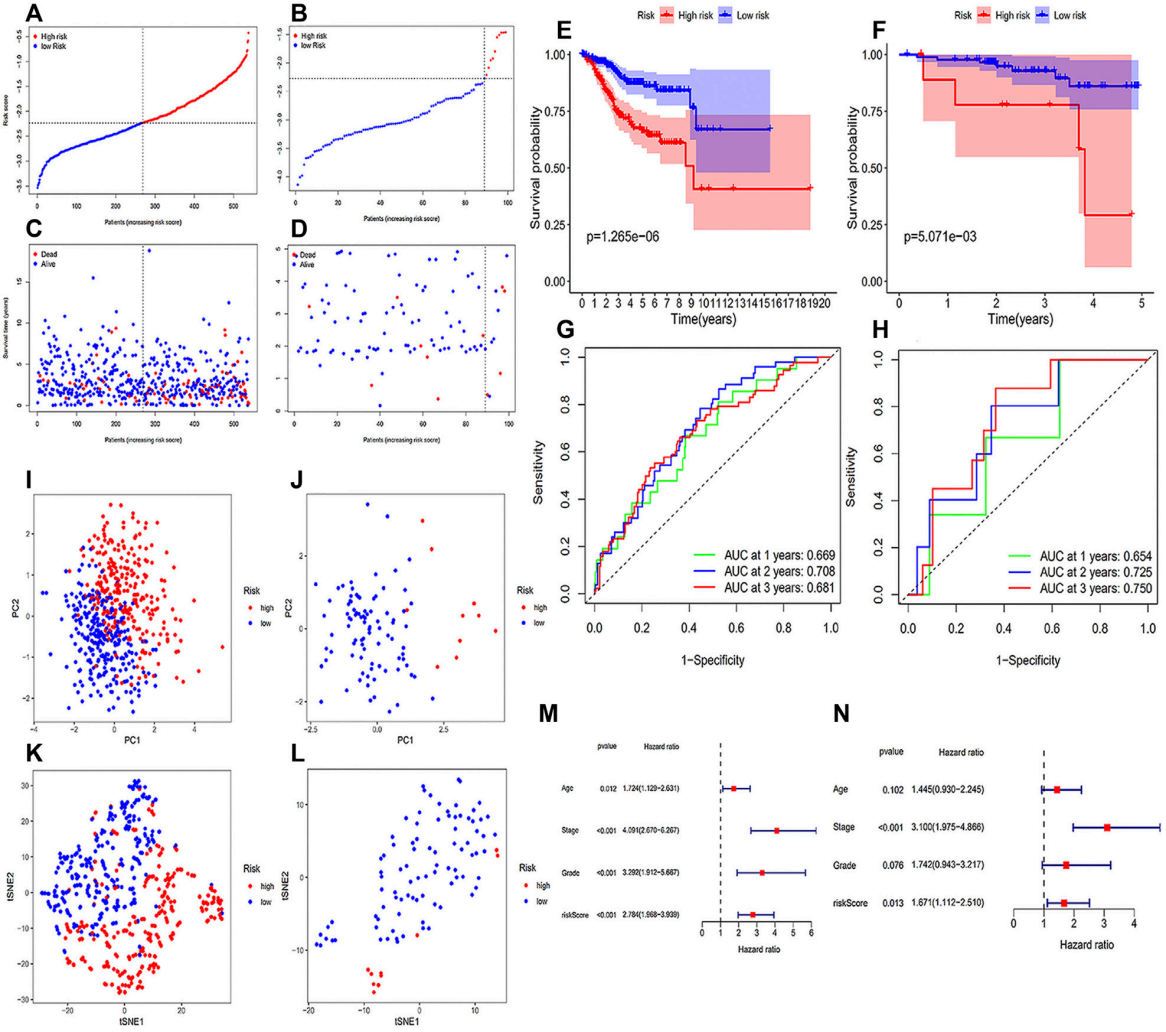
Construction and validation of the prognostic signature. **(A)** The distribution and median value of the risk scores of patients from the training cohort; **(B)** The distribution and median value of the risk scores of patients from the testing cohort; **(C)** Survival status distribution in the training cohort; **(D)** Survival status distribution in the testing cohort. **(E)** The Kaplan-Meier curves for the OS of patients in the training group; **(F)** the Kaplan-Meier curves for the OS of patients in the testing group; **(G)** the ROC curves in the training group; **(H)** the ROC curves in the testing group. **(I)** PCA plot of the training cohort; **(J)** PCA plot of the testing cohort; **(K)** t-SNE analysis result of the training cohort; **(L)** t-SNE analysis result of the testing cohort. **(M**,**N)** Independent prognostic factors in the univariate **(M)** and multivariate Cox regression **(N)**.

Table 1 showed the coef value of each gene, and the risk score of each sample = (gene1 expression * gene1 coef) + (gene2 expression * gene2 coef) + … + (gene6 expression * gene6 coef). With this method, we determined the risk scores of each sample in two databases, and patients from CPTAC were categorized into two groups with different risks in accordance with the median value in the TCGA database (Figures 2A–D). It was clear that high-risk patients showed a worse survival probability than those with low-risk scores in the Kaplan-Meier curves (Figures 2E,F). And the area under the curve (AUC) of the ROC curve were 0.669, 0.708 and 0.681 at 1, 2 and 3 years, respectively, in the training group (Figure 2G). Similarly, the 1-, 2- and 3-year AUC were 0.654, 0.725 and 0.750 in the testing group (Figure 2H). The results of PCA and t-NSE indicated that the EC patients could be well distinguished according to our risk prediction model (Figures 2I–2L). Taken together, our FRGs signature performed well in predicting prognosis in both training and testing groups.

**TABLE 1:** The coefficient of each gene.

Gene	Coef
TP53	−0.116722317967062
AIFM2	−0.22722405952422
ATG7	−0.349280187919115
TLR4	−0.170943566583093
PANX1	0.525584244027421
MDM2	−0.504409372893519

Coef, coefficient.

The test results of the core FRGs in other cancers suggested that these genes were also significantly associated with multiple cancers survival, including prostate cancer, urothelial cancer, renal cancer, colorectal cancer, testis cancer and cervical cancer (Supplementary Figure S1). Except that the high expression of TLR4 was associated with the poor prognosis of testis cancer, the effects of other genes on the survival of patients with different tumors were all consistent with that of our study. To be a bit more specific, TP53, AIFM2, ATG7 and MDM2 were favorable genes for tumor patients survival, while PANX1 represented a favorable factor affecting the prognosis of patients with renal cancer.

### Independent Prognostic Factor for Overall Survival in Endometrial Cancer Patients

Age, grade, FIGO stage and risk score were brought into the univariate and multivariate Cox regression analyses. As illustrated in Figure 2M, univariate Cox analysis implied that all age, grade, FIGO stage and risk score were significantly correlated with survival. Furthermore, the multivariate Cox analysis results demonstrated that both FIGO stage [hazard ratio (HR): 3.100; 95% confidence interval (CI): 1.975-4.866] and risk score (HR: 1.671; 95% CI: 1.112-2.510) were independent prognostic factors in OS of EC patients (Figure 2N).

### Gene Ontology and Kyoto Encyclopedia of Genes and Genomes Pathway Enrichment Analysis

**FIGURE 3:**
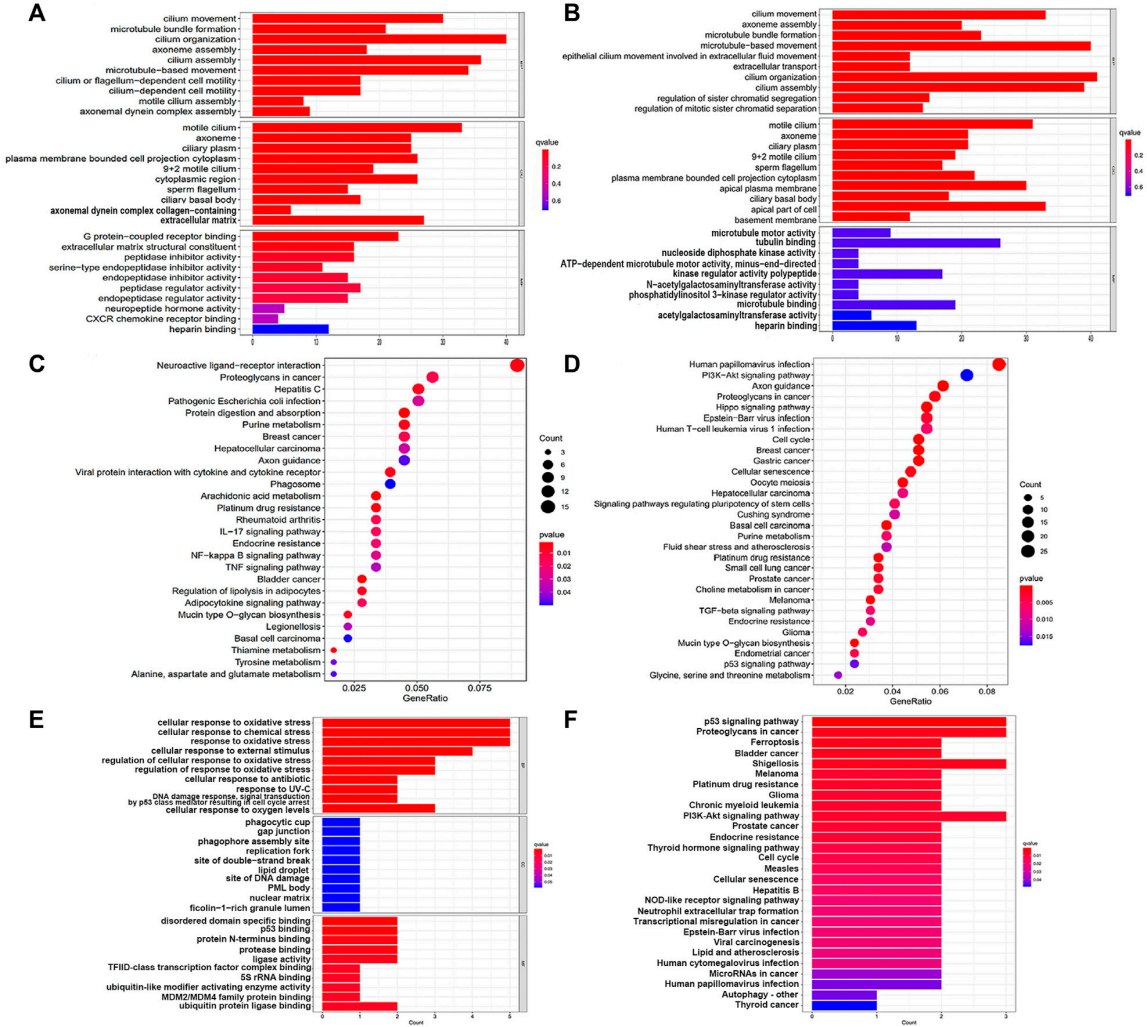
GO and KEGG enrichment analysis results. **(A**,**C)** GO and KEGG pathway enrichment analysis in the training cohort; **(B**,**D)** GO and KEGG pathway enrichment analysis in the testing cohort; **(E,F)** GO and KEGG pathway enrichment analysis of the core genes in this signature.

Differential genes between two groups were used for GO and KEGG analysis (Figures 3A–D). It was observed from the biological processes category that these genes were mainly involved in cilium movement, axoneme assembly and cilium organization. And the cellular compounds results demonstrated significant enrichment of these genes in motile cilium, axoneme and ciliary plasm. For the category of molecular functions, genes had a marked relationship with G protein-coupled receptor binding and microtubule motor activity.

It was noteworthy that we found many cancer and immune-related pathways-related pathways, such as ‘Proteoglycans in cancer’, ‘Hepatocellular carcinoma’, ‘Breast cancer’, ‘Gastric cancer’, ‘Endometrial cancer’, ‘Platinum drug resistance’, ‘p53 signaling pathway’, ‘Cellular senescence’, ‘Cell cycle’, ‘NF-kappa B signaling pathway’ and ‘Human T-cell leukemia virus 1 infection’ from the results of KEGG analysis. In addition, these genes were also involved in ferroptosis-associated pathways, including ‘Arachidonic acid metabolism’, ‘PI3K-Akt signaling pathway’ and ‘Glycine, serine and threonine metabolism’.

Correspondingly, we carried out enrichment analysis for the 6 FRGs to gain more insight into the function of core genes in this signature (Figures 3E,F). GO term showed these genes mainly took part in the biological processes of ‘cellular response to oxidative stress’, ‘cellular response to chemical stress’ and ‘response to oxidative stress’. Remarkably, we noticed that these genes were signaficantly enriched in ‘ferroptosis pathway’ and some cancer-related pathways, suggestting the strong possibility of genes to affect tumor initiation and progression in a ferroptosis manner.

### Immune Profile

**FIGURE 4:**
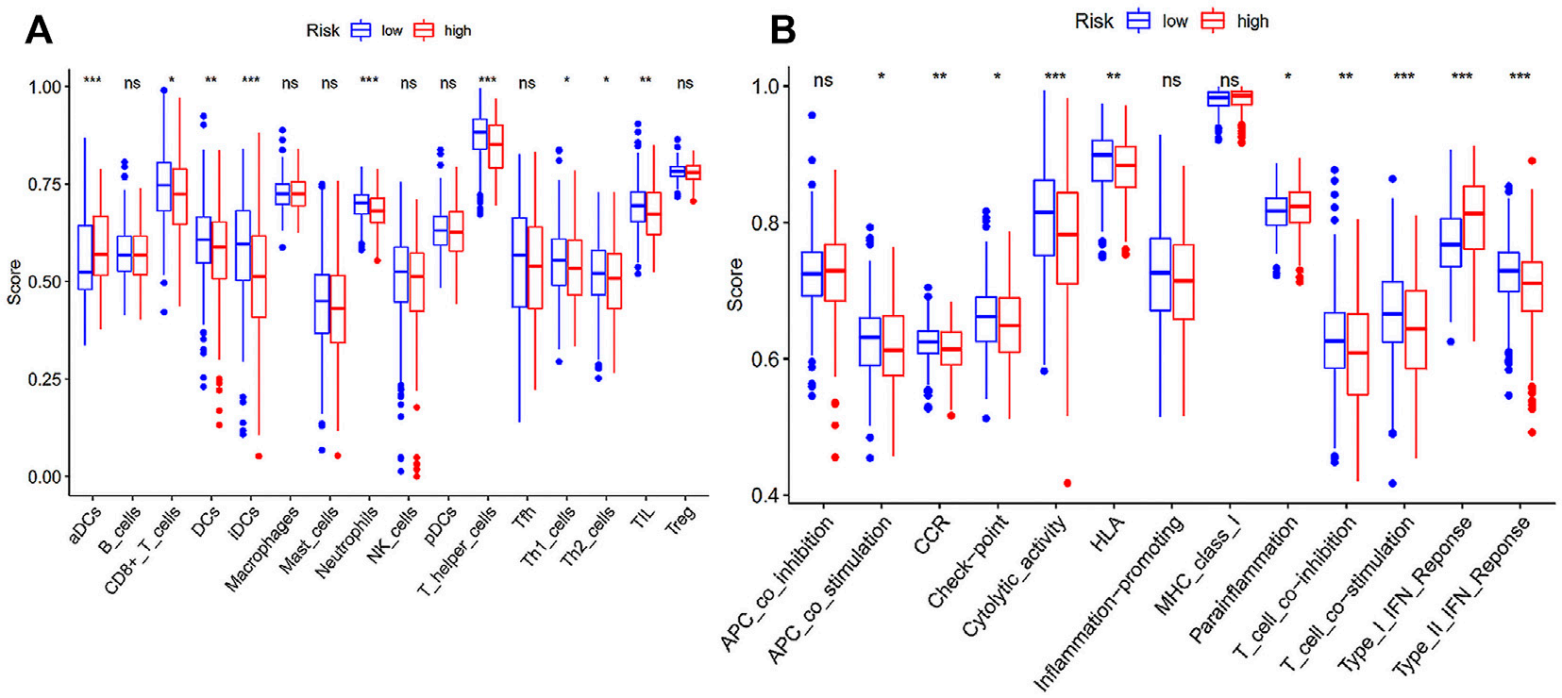
Immune status between high- and low-risk group. **(A)** Scores of 16 immune cells; **(B)** Scores of 13 immune-related functions; *p < 0.05; **p < 0.01; ***p < 0.001.

Considering ferroptosis has been documented to play an indispensable part in anti-tumor immunity, we performed ssGSEA to quantify the differences in immune status in our risk model (Figure 4). It was apparent from the ssGSEA results that there was a significant difference in the infiltration of immune cells between the two different risk states on the basis of the immune scores. CD8^+^ T cells, dendritic cells (DCs), interstitial DCs, neutrophils, T helper cells, Th1, Th2 cells and tumor-infiltrating lymphocytes (TILs) had higher infiltrating scores in the low-risk group. In other words, high abundance of these immune cells may be connected to a relatively good prognosis in EC patients. On the contrary, activated DCs displayed a higher infiltration in high-risk patients. In addition, we can notice that the enrichment of immune functions like antigen-presenting cell (APC) coinhibition, type I interferons (IFN) response and parainflammation were more significant in patients with high-risk scores, while type II IFN response, chemokine receptor (CCR), cytolytic activity, check point, human leukocyte antigen, T cell costimulation and T cell coinhibition were more significantly enriched in low-risk patients.

**FIGURE 5:**
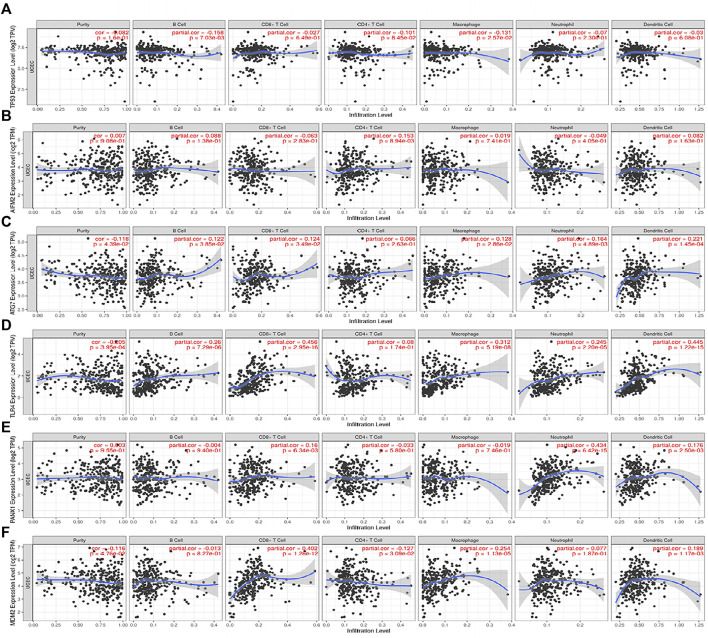
The correlations between the expression of 6 FRGs and immune cells from the TIMER database. **(A)** TP53 and immune cells; **(B)** AIFM2 and immune cells; **(C)** ATG7 and immune cells; **(D)** TLR4 and immune cells; **(E)** PANX1 and immune cells; **(F)** MDM2 and immune cells.

Furthermore, the 6 FRGs were imported into the TIMER database (https://cistrome.shinyapps.io/timer/) (Li et al., 2017) separately to explore the correlation between single FRG and immune infiltration in TCGA (Figure 5). MDM2 was negatively correlated with CD4+T cells (*p* = 0.0309), but correlated positively with CD8+T cells, macrophages and DC (*p* < 0.05). AIFM2 had a positively correlation with CD4+T cells (*p* = 0.00894), and ATG7 positively correlated with B cells, CD8+T cells, macrophages, neutrophils and DC (*p* < 0.05). In addition, there should be a significantly positive correlation between TLR4 and such immune cells, including B cells, neutrophils, macrophages, CD8+T cells and DC (*p* < 0.001). PANX1 expression level was positively correlated with neutrophils, CD8+T cells and DC (*p* < 0.01), and TP53 correlated negatively with B cells and macrophages (*p* < 0.05).

### Analysis in Response to Chemotherapy

**FIGURE 6:**
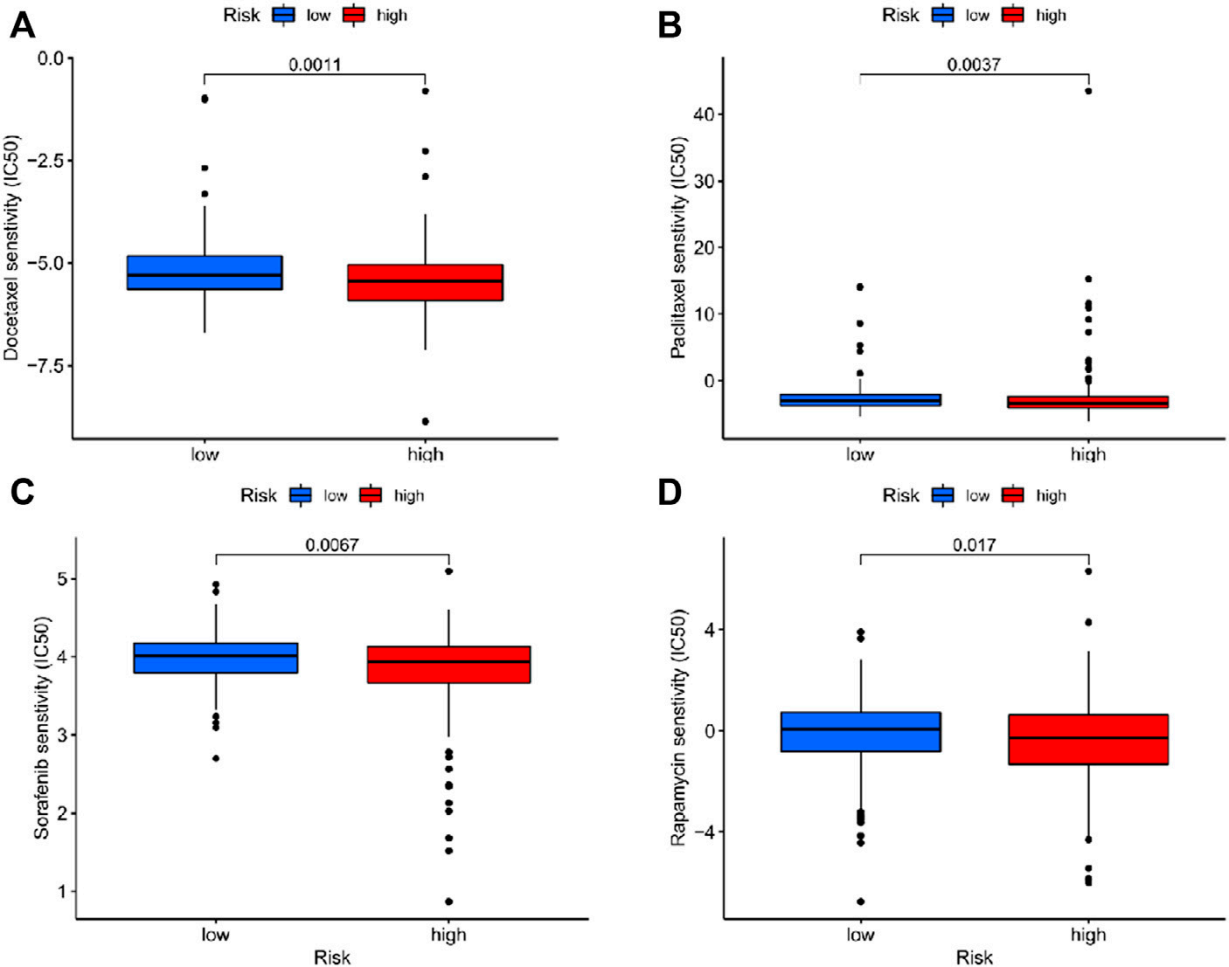
The correlation between different risk groups and drug sensitivity in EC patients. **(A)** Docetaxel; **(B)** Paclitaxel; **(C)** Sorafenib; **(D)** Rapamycin.

There was a significant difference in the estimated half-maximal inhibitory concentration (IC50) between two risk cohorts (Supplementary Presentation S1). Crucially, compared with the low-risk patients, IC50 values of docetaxel, paclitaxel, sorafenib and rapamycin were lower in the high-risk group (Figure 6), which indicated that the patients with high-risk scores had a more sensitive response to chemotherapy commonly applicated in EC treatment.

## Discussion

In order to realize the landscape expansion in EC therapeutic, gigantic efforts have been devoted to unveiling the role of ferroptosis in inducing EC cell death over recent years (Wang et al., 2021a; Zhang M. et al., 2021; Zhang Y. Y. et al., 2021). Moreover, previous studies have attempted to explore the feasibility of ferroptosis in prognosis prediction of EC patients (Liu et al., 2021; Wang et al., 2021b; Weijiao et al., 2021). Compared with them, we have incorporated more updated FRGs in this research. Meanwhile, we directly identified DEGs between the whole EC samples and normal samples, rather than, as in other similar studies, only half of the EC samples were involved in the process due to the existence of internal verification. Both of them make our research more comprehensive and scientific. Futhermore, the conclusions of this study have been verified by an external database rigorously, which is precisely lacking in other studies. In the present study, we first constructed a prognostic model that integrated 6 FRGs in TCGA, namely, TP53, AIFM2, ATG7, TLR4, PANX1 and MDM2. EC patients were categorized into high- and low-risk groups in this signature according to the expression levels of the FRGs and the corresponding coef value. It was found that low-risk cases had a significant survival advantage, which was verified by data from the CPTAC database. Finally, we discovered significant differences between high- and low-risk samples in the immune infiltration according to the prognostic signature. It provides brilliant ideas for improving the prognosis evaluation system and immunotherapy in EC patients.

As taught in the traditional sense, P53, the tumor suppressor, is involved in controlling cell division and survival under a variety of stresses, including cell cycle apoptosis and autophagy. In the past few years, It has been confirmed in both animal models and cell cultures that p53 stands for a fresh ferroptosis regulator (Jiang et al., 2015; Ou et al., 2016; Xie et al., 2017; Tarangelo et al., 2018). Unfortunately, the bidirectional control of p53 in the network of ferroptosis remains unknown and needs to be identified according to the context. In combination, our results indicated a favorable effect of P53 on prognosis in EC patients. AIFM2, a DNA-binding oxidoreductase protein derived from the mitochondria, is characterized as an endogenous ferroptosis suppressor. Remarkably, it has been reported that AIFM2 protects cancer cells from erastin-, sorafenib-, and RSL3-induced ferroptosis via an approach independent of ubiquinol (Bersuker et al., 2019; Doll et al., 2019). Similarly, several studies have shown that AIFM2 represents an unfavorable prognostic factor in prostate cancer, acute myeloid leukemia and uveal melanoma (Luo and Ma, 2021; Lv et al., 2021; Song et al., 2021). However, a recent study has indicated that AIFM2 act as a favorable factor for OS of gastric cancer patients, which is consistent with the findings of our study (Shao et al., 2021). Therefore, it is interesting to explore the effect of AIFM2 on the prognosis of different types of tumors. ATG7, known as a vital autophagy-related gene, exerts a major impact on the formation of the autophagosome. ATG5-ATG7-NCOA4 pathway is critical for the regulation of iron metabolism and knockout of ATG7 can lead to a limited elastin-induced ferroptosis in pancreatic cancer cell lines (Hou et al., 2016). It is an embodiment of the interplay between ferroptosis and autophagy. Likewise, ATG7 predicts a good prognosis in EC patients in our research. Gene TLR4 encodes a transmembrane protein TLR4, responsible for regulating the innate immune response (Moresco et al., 2011). *In vitro* and *in vivo* experiments have confirmed that the activation of TLR4 contributes to neuronal ferroptosis through TLR4-p38 MAPK signaling pathway (Zhu et al., 2021). In addition, TLR4/TRIF/type I IFN signaling pathway participates in the recruitment of neutrophils to the injured myocardium in the process of ferroptosis (Li et al., 2019). Notably, TLR4 agonists have aroused widespread concern as a brilliant immunotherapeutic for the treatment of cancer (Shetab Boushehri and Lamprecht, 2018). Recently, protective effects of TLR4 have been identified on the outcome for lung adenocarcinoma (Ren et al., 2021; Zhang A. et al., 2021), which are in good agreement with our signature in EC. More significantly, TLR4 expression is severely attenuated in EC tissues, while its expression levels positively correlate with the infiltration of multiple immune cell types significantly, including B cell (cor = 0.26, *p* = 7.29e−06), CD8^+^ T cell (cor = 0.456, *p* = 2.95e−16), macrophages (cor = 0.312, *p* = 5.19e−08) and dendritic cells (cor = 0.445, *p* = 1.22e−15). Hopefully, these findings may thus offer exciting research prospects for immunotherapy of EC. Serving as an E3 ligase, MDM2 has been considered a negative regulator of p53 which can mediate it ubiquitination (Karni-Schmidt et al., 2016). Nevertheless, previous research has also revealed that MDM2 facilitates ferroptosis in cells with or without p53 by altering PPARα activity (Venkatesh et al., 2020). Although MDM2 downregulation in EC tissues may inhibit the progression of EC via suppressing the migration, invasion and anti-apoptosis of EC cells (Liu et al., 2019). The effect of MDM2 expression on the prognosis of EC is still controversial. But it is undeniable that there is a strong positive correlation between the expression of MDM2 and CD8^+^ T cells in the tumor microenvironment.

As a damage-associated molecular pattern (DAMP) molecule, PANX1 represents a key player in modulating ATP release. And PANX1 knockout protects against renal ischemia/reperfusion injury through attenuating MAPK/ERK activation in a ferroptotic pathway. Moreover, it is reported that PANX1 promotes tumorigenesis in breast cancer through EMT pathway.

Damage-related molecules are released along with the process of ferroptosis, which are perceived by immune cells to amplify the inflammatory response. With the development of modern medicine, people are increasingly conscious of the crucial role of the immune microenvironment in cancer treatment (López-Janeiro et al., 2021). In this article, we performed ssGSEA to evaluate the immune status in different risk groups. Undoubtedly, in contrast to the previous model (Weijiao et al., 2021), low-risk patients were present with more abundant immune cells infiltration and immune functions enrichment in ours. As the most important anti-tumor effector cells, activated CD8^+^ T cells eliminate tumor cells through recognition by their expressed T cell receptors of tumor-associated antigens on major histocompatibility complex I (MHC I) (Gajewski et al., 2013). The high infiltration of CD8^+^ T cells is associated with favorable survival outcomes in EC (Qin et al., 2021). DCs bridge the gap between the innate and the adaptive immune systems (Hinshaw and Shevde, 2019). And numerous strategies in the fight against cancer have been developed, which exactly target DCs (Wculek et al., 2020). Th1 and Th2, T helper cells subclasses, are involved in molecular crosstalk among different immune signaling pathways, whose relevance in tumor immunotherapy is under investigation. In particular, Th1 cells can kill tumor cells directly by activating death receptors on the surface of them, enhance CD8^+^ T cells priming and expansion via cytokines release and promote natural killer cells and type I macrophages to recruit to tumors (Knutson and Disis, 2005; LaCasse et al., 2011; Kim and Cantor, 2014). In addition, increased Th1 cells have been reported to be associated with protracted disease-free survival in colorectal cancer patients (Tosolini et al., 2011).

The decline of human leukocyte antigen (HLA) function will cause the loss of HLA-I expression on the surface of tumor cells, thereby leading to deficiencies in the antigen presentation pathway and helping tumors escape from immunotherapy (2018; Hazini et al., 2021). It is clear that the HLA function was significantly insufficient in high-risk patients. Parainflammation is a low-grade inflammatory response between primary dynamic balance and chronic inflammation. Animal experiments have suggested that parainflammation attenuation can prevent tumor growth, which may be related to its inability to recruit immune cells (Lasry et al., 2017). The antitumoral immune cytolytic activity of the patients is calculated from the geometric mean of perforin 1 (PRF1) and granzyme A (GZMA) genes expression (Rooney et al., 2015; Narayanan et al., 2018). While the overexpression of these two effector molecules is upon the activation of CD8+T cells and high scores of checkpoint in the low-risk group (Johnson et al., 2003). This also validates the emphasis that neoadjuvant therapy combined with multiple immune checkpoints expands the clinical benefits of tumor patients (Zaravinos et al., 2019). To add to that, coupled with the poor enrichment of APC costimulation, type II IFN response and T cell costimulation, the immune response to EC cells is suppressed in the high-risk group.

Furthermore, the response to chemotherapy in high-risk patients was significantly stronger than that in the low-risk group, which may provide more rational choices in the treatment for patients at a high risk.

## Conclusion

In the current study, we identified a prognostic signature consisting of 6 FRGs with novel forecast value in the OS of EC. Significantly, we verified the results based on an external database for the first time. The ssGSEA results pointed out a tight link between ferroptosis and the tumor immune microenvironment, which is expected to provide a new prospect for the immunotherapy of EC in future work.

## Data Availability

The datasets presented in this study can be found in online repositories. The names of the repository/repositories and accession number(s) can be found below: https://portal.gdc.cancer.gov/, TCGA-UCEC; https://proteomics.cancer.gov/programs/cptac, 3.

## Abbreviations

EC, endometrial cancer; FRGs, ferroptosis-related genes; TCGA, The Cancer Genome Atlas; CPTAC, Clinical Proteomic Tumor Analysis Consortium; FDR, false discovery rate; OS, overall survival; LASSO, least absolute shrinkage and selection operator; ROC, receiver operating characteristic; t-SNE, t-Distributed Stochastic Neighbor Embedding; PCA, Principal component analysis; FC, fold change; GO, Gene Ontology; KEGG, Kyoto Encyclopedia of Genes and Genomes; ssGSEA, Single sample gene set enrichment analysis; PPI, protein-protein interactions; AUC, area under the curve; HR, hazard ratio; CI, confidence interval; DCs, dendritic cells; TILs, tumor-infiltrating lymphocytes; APC, antigen-presenting cell; IFN, interferons.
