# Supplementary figures and images for: Prediction of Prognosis in Patients With Endometrial Carcinoma and Immune Microenvironment Estimation Based on Ferroptosis-Related Genes
Source: Front Mol Biosci. 2022 Jul 15;9:916689. doi: 10.3389/fmolb.2022.916689 (PMC9334791; doi:10.3389/fmolb.2022.916689)

Risk 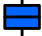 low 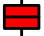 high

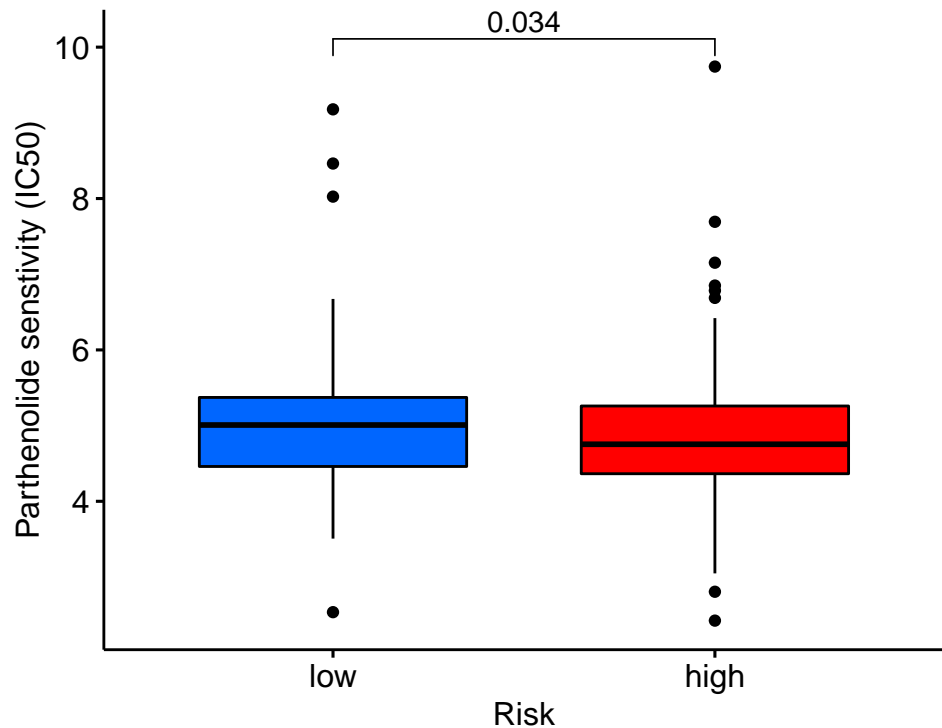

Supplement: Supplementary file 2 [file Presentation1.zip › durgSenstivity.Parthenolide.pdf]

Risk 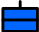 low 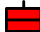 high

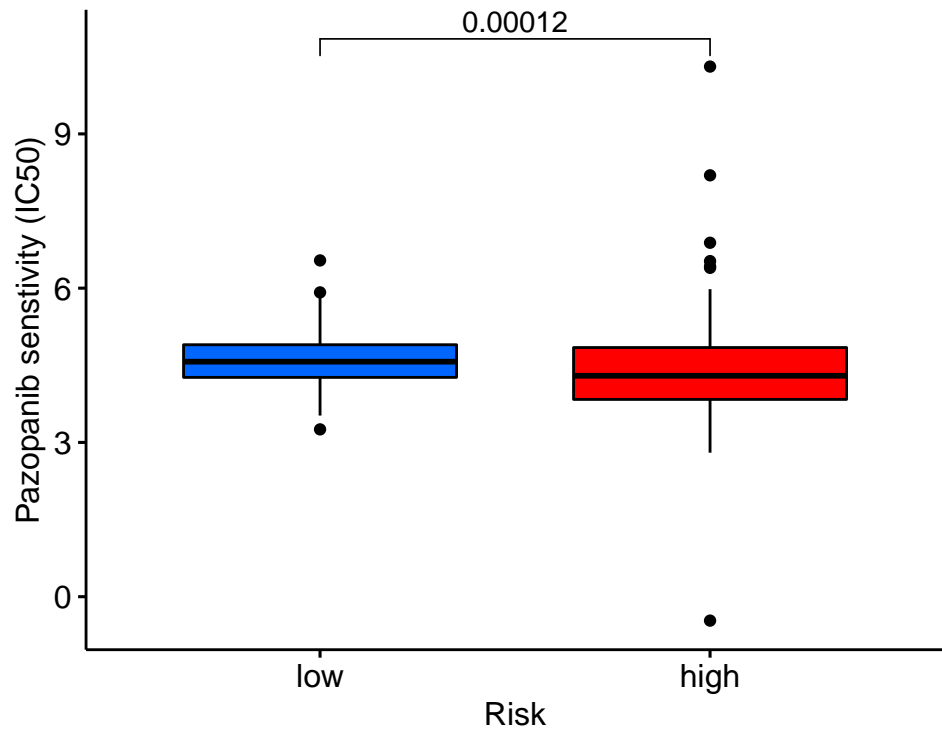

Supplement: Supplementary file 2 [file Presentation1.zip › durgSenstivity.Pazopanib.pdf]

Risk 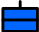 low 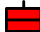 high

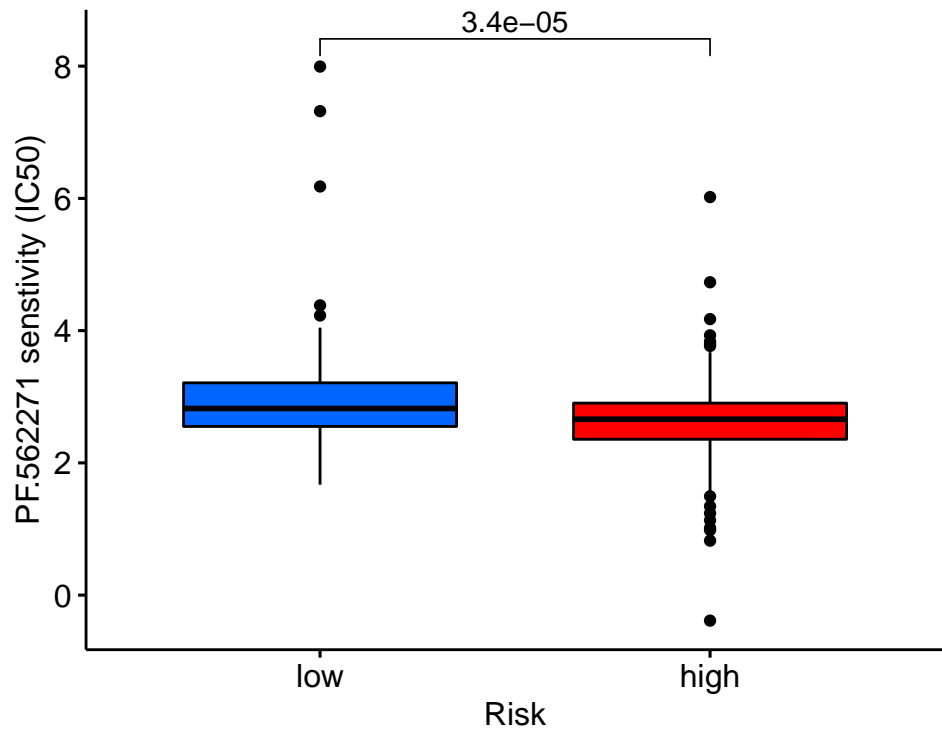

Supplement: Supplementary file 2 [file Presentation1.zip › durgSenstivity.PF.562271.pdf]

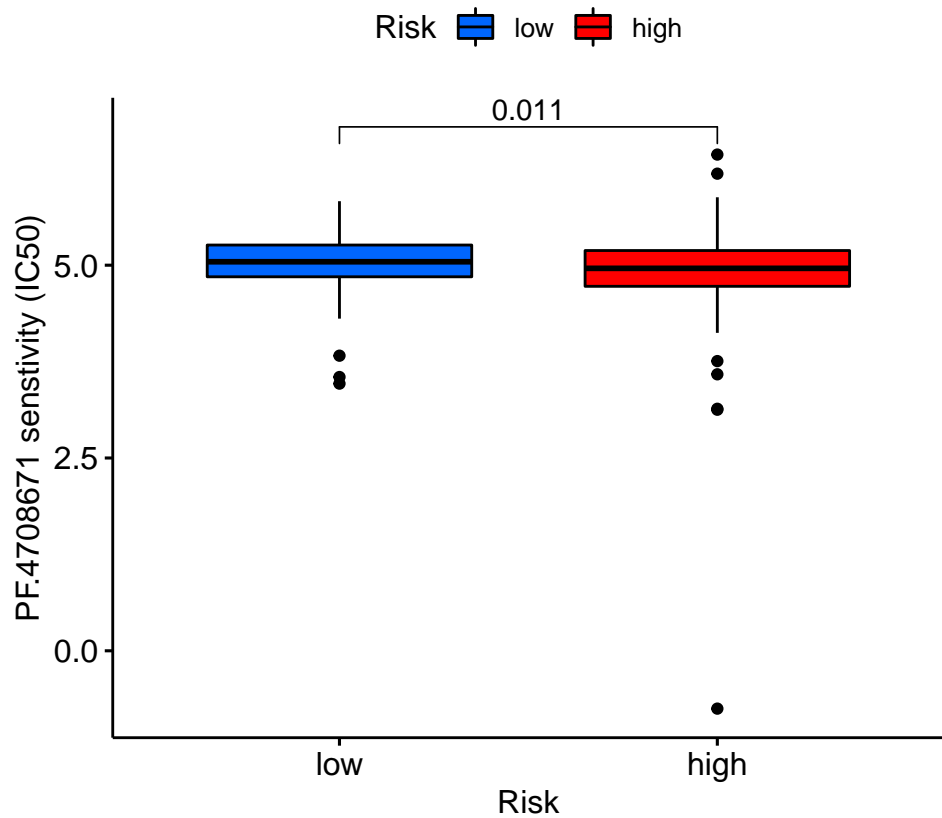

Supplement: Supplementary file 2 [file Presentation1.zip › durgSenstivity.PF.4708671.pdf]

Risk 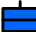 low 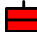 high

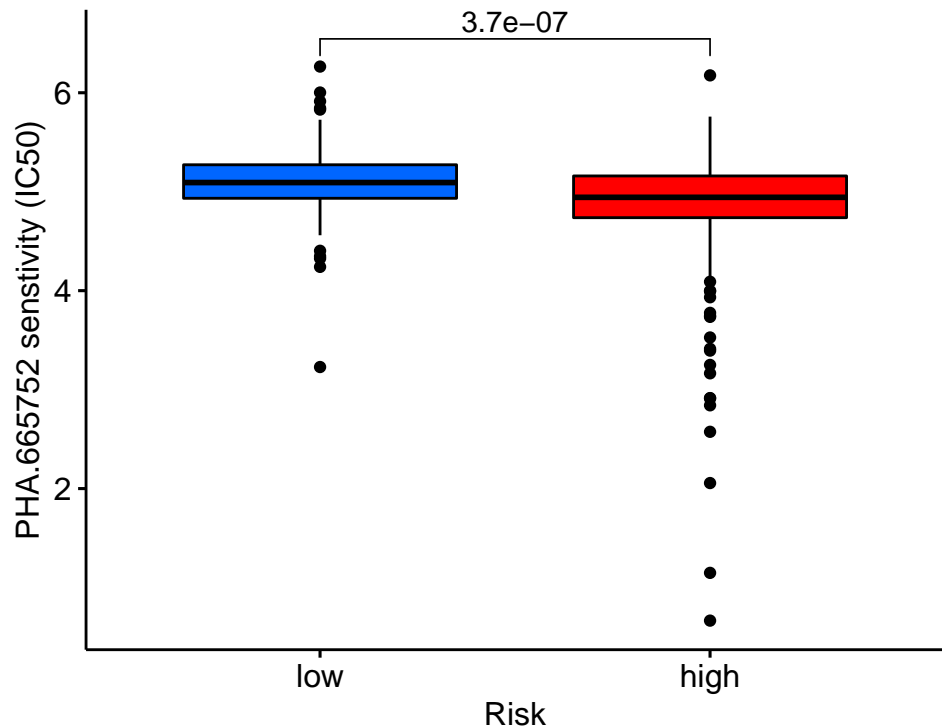

Supplement: Supplementary file 2 [file Presentation1.zip › durgSenstivity.PHA.665752.pdf]

Risk 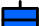 low 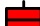 high

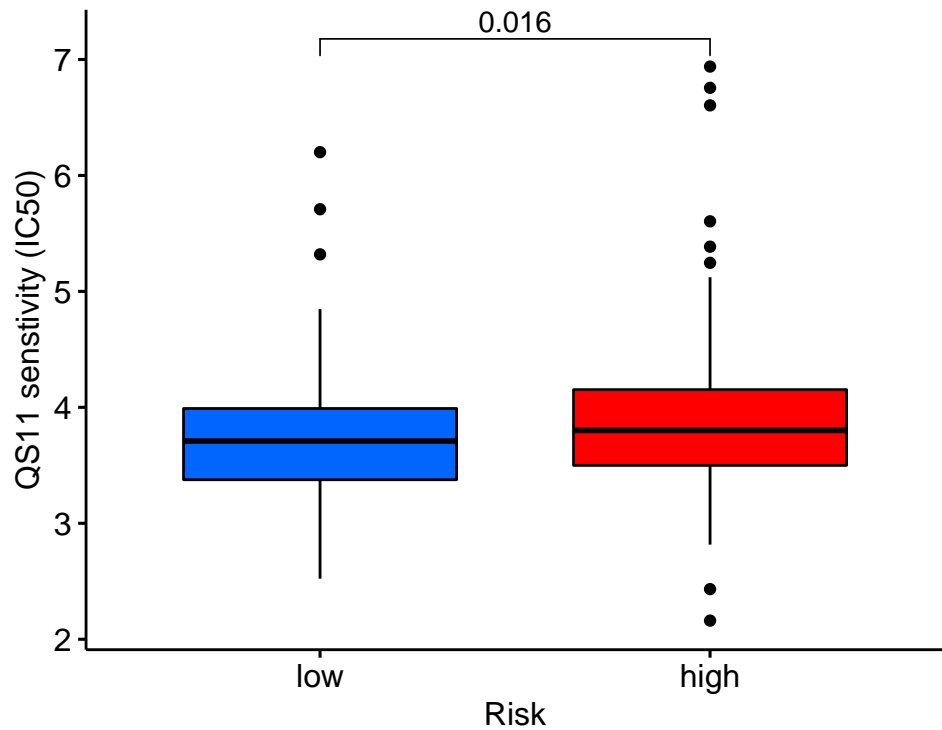

Supplement: Supplementary file 2 [file Presentation1.zip › durgSenstivity.QS11.pdf]

Risk 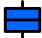 low 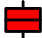 high

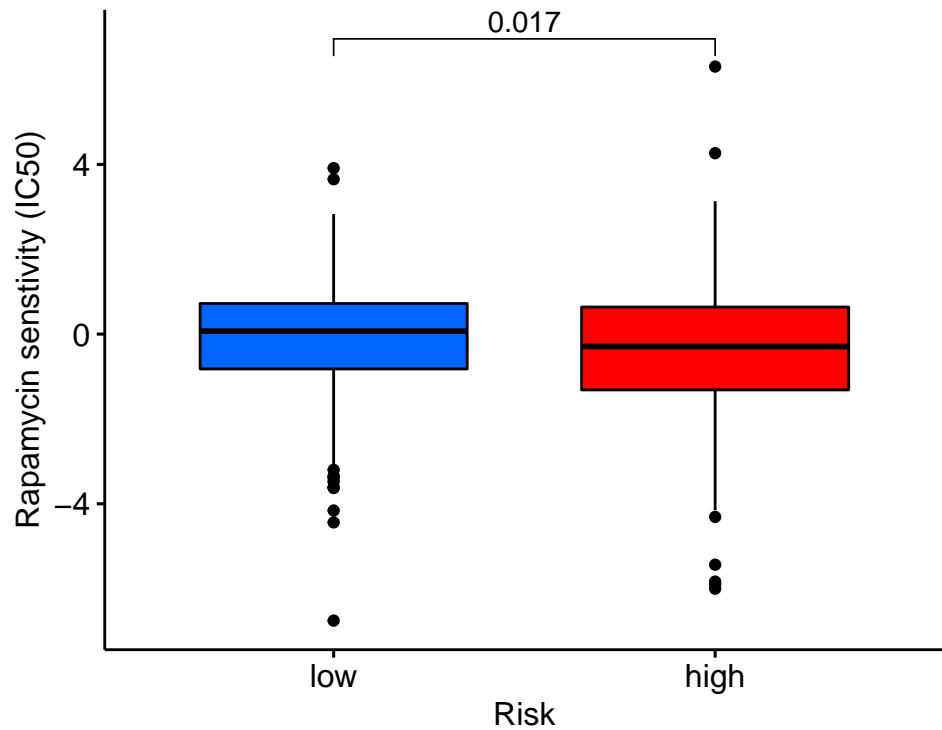

Supplement: Supplementary file 2 [file Presentation1.zip › durgSenstivity.Rapamycin.pdf]

Risk 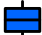 low 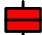 high

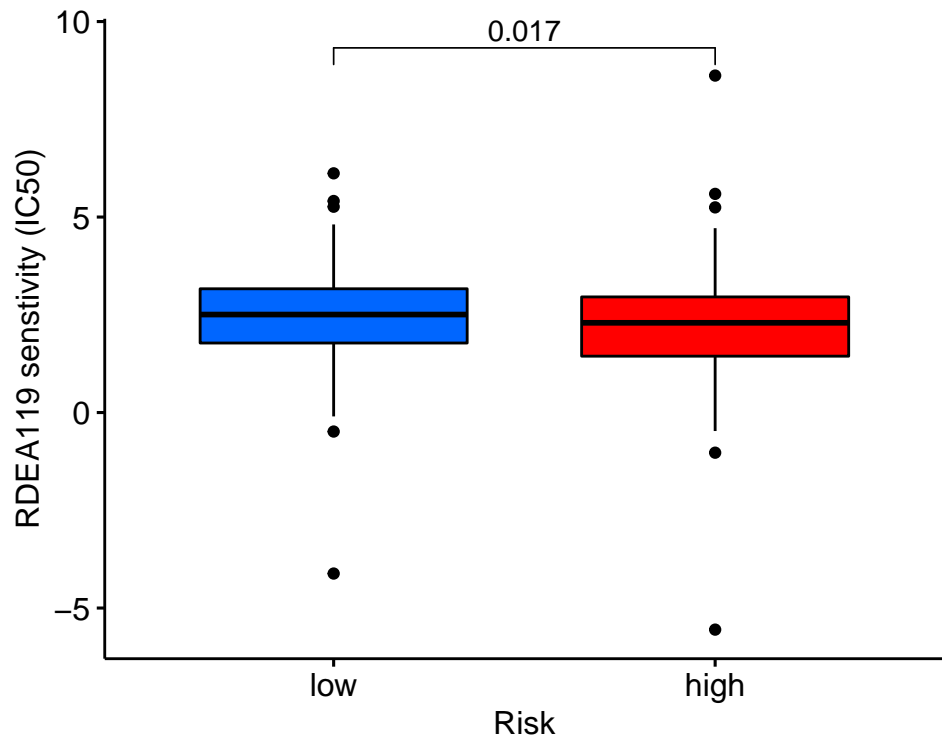

Supplement: Supplementary file 2 [file Presentation1.zip › durgSenstivity.RDEA119.pdf]

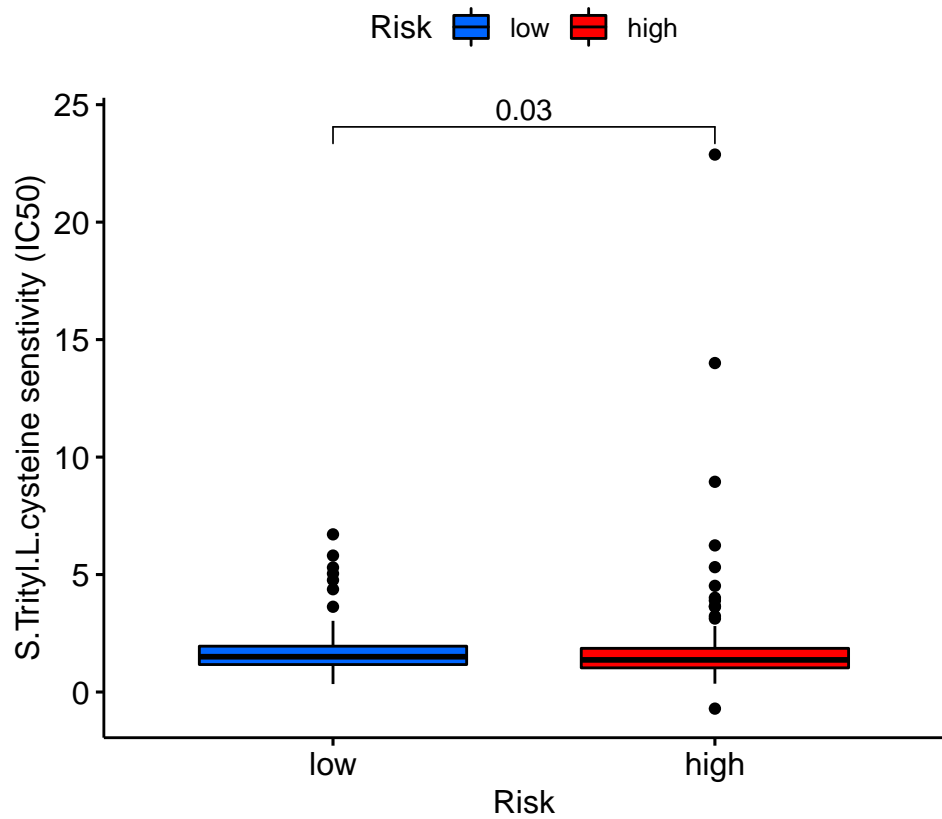

Supplement: Supplementary file 2 [file Presentation1.zip › durgSenstivity.S.Trityl.L.cysteine.pdf]

Risk 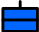 low 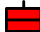 high

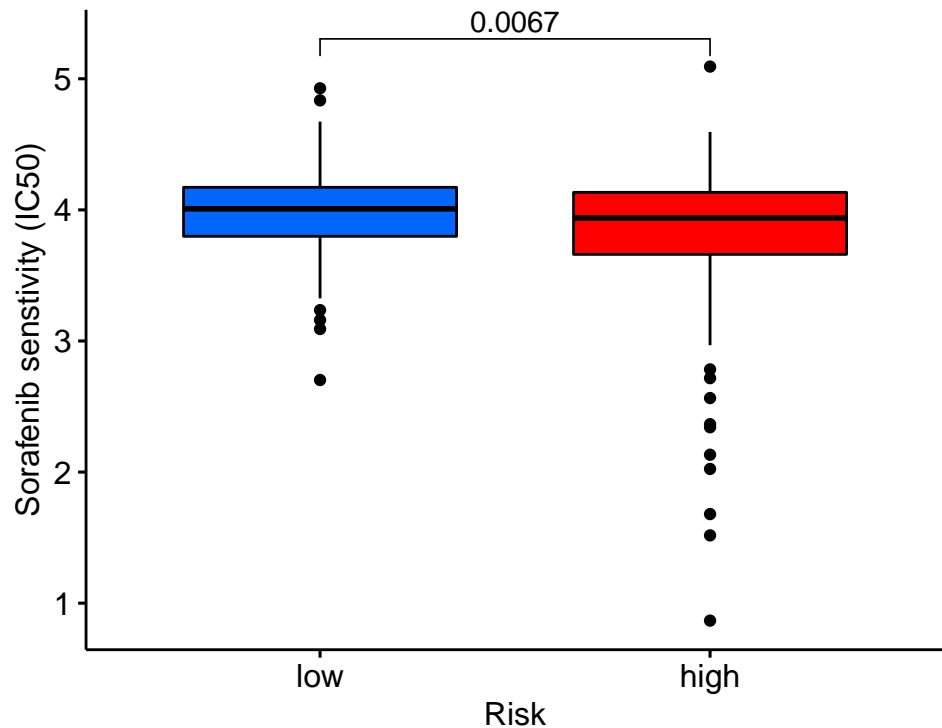

Supplement: Supplementary file 2 [file Presentation1.zip › durgSenstivity.Sorafenib.pdf]

Risk 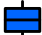 low 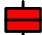 high

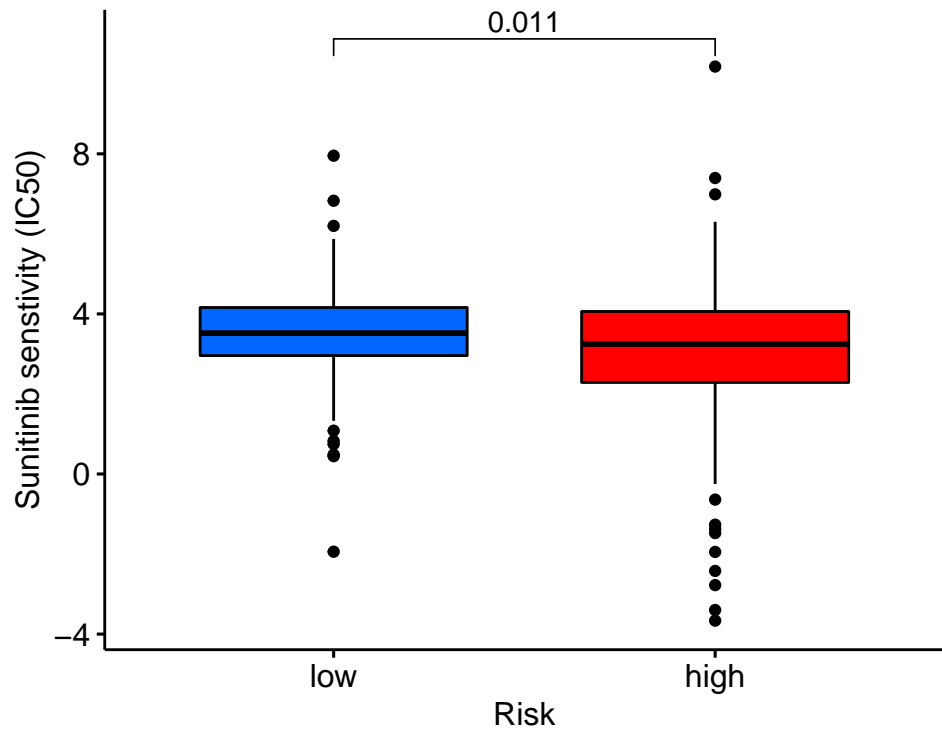

Supplement: Supplementary file 2 [file Presentation1.zip › durgSenstivity.Sunitinib.pdf]

Risk 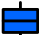 low 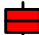 high

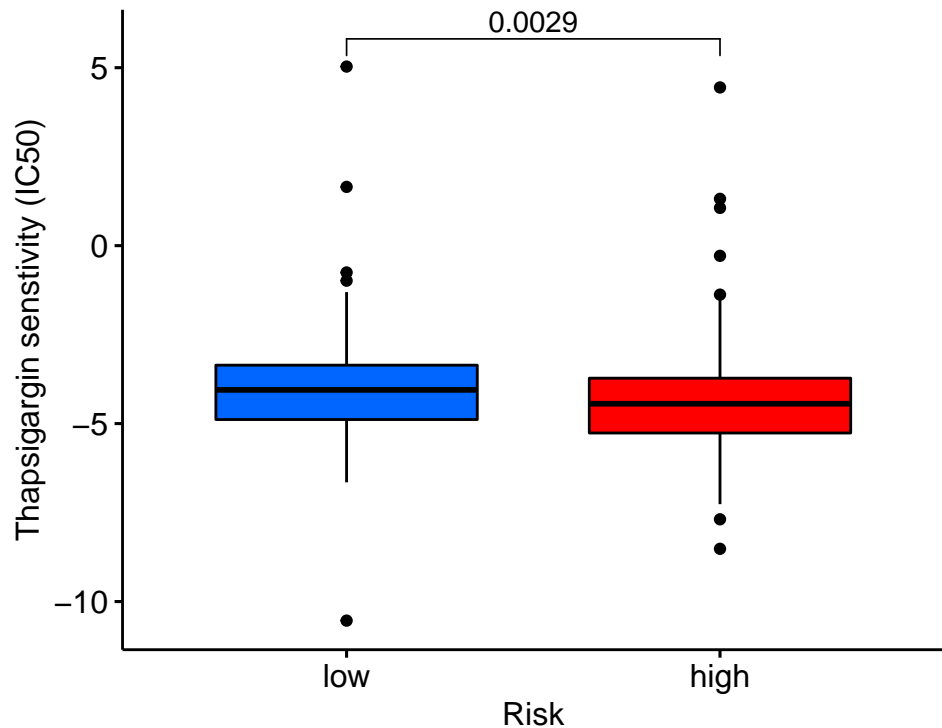

Supplement: Supplementary file 2 [file Presentation1.zip › durgSenstivity.Thapsigargin.pdf]

Risk low high

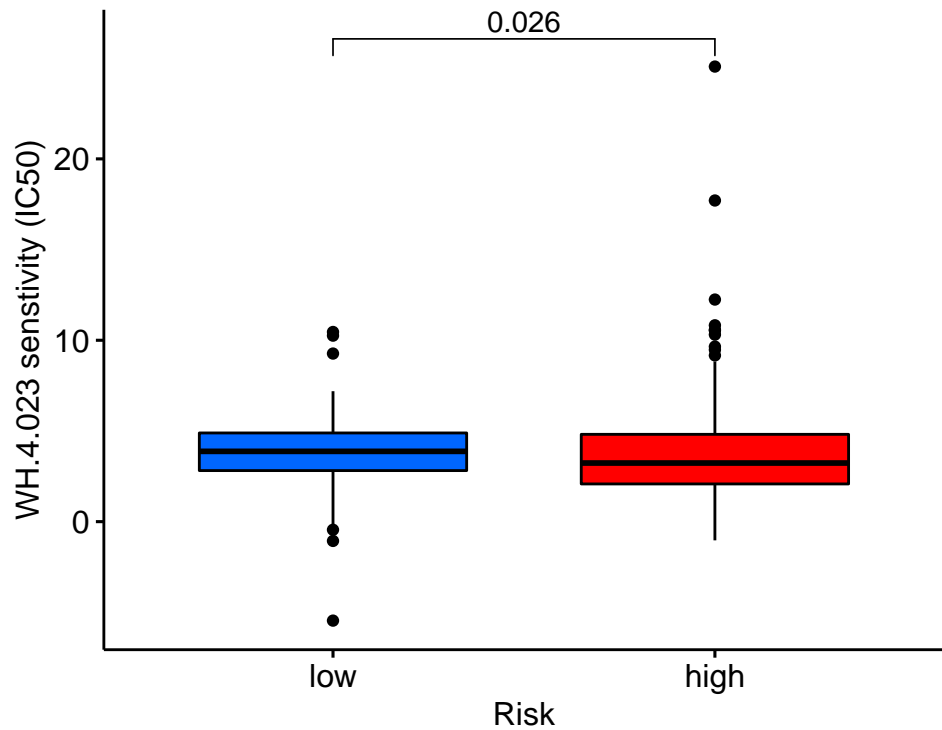

Supplement: Supplementary file 2 [file Presentation1.zip › durgSenstivity.WH.4.023.pdf]

Risk 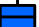 low 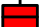 high

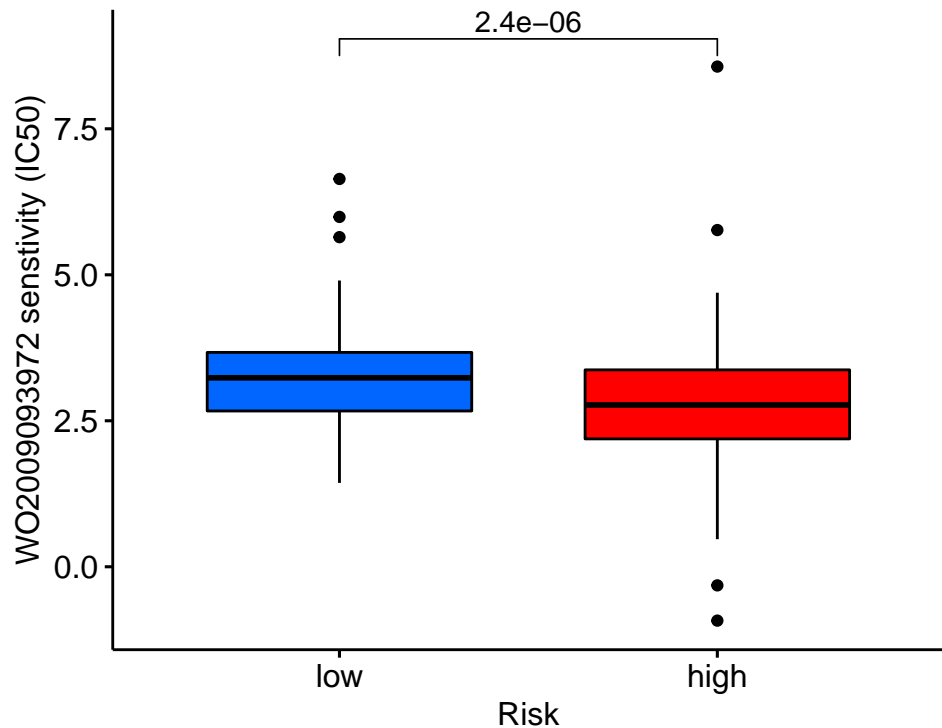

Supplement: Supplementary file 2 [file Presentation1.zip › durgSenstivity.WO2009093972.pdf]

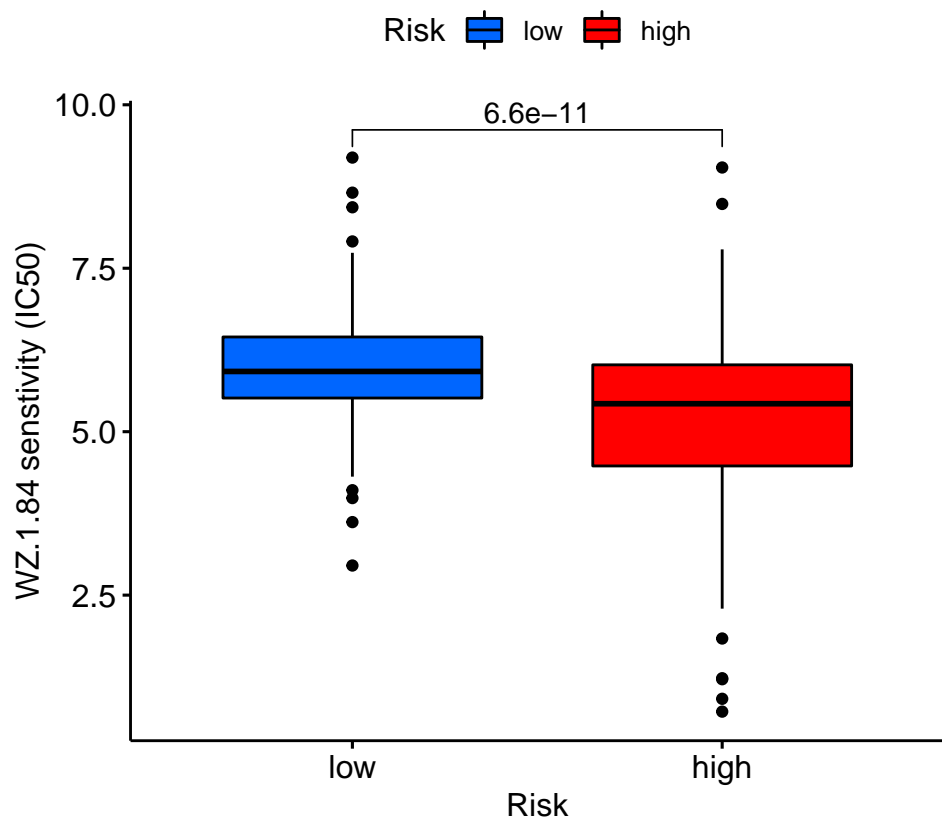

Supplement: Supplementary file 2 [file Presentation1.zip › durgSenstivity.WZ.1.84.pdf]

Risk 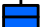 low 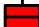 high

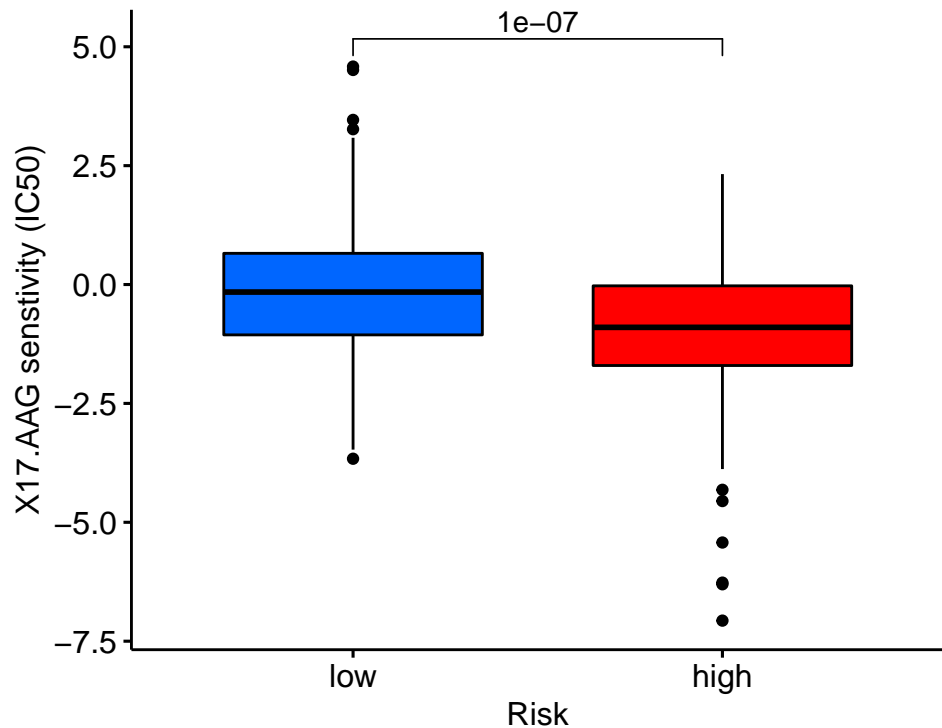

Supplement: Supplementary file 2 [file Presentation1.zip › durgSenstivity.X17.AAG.pdf]

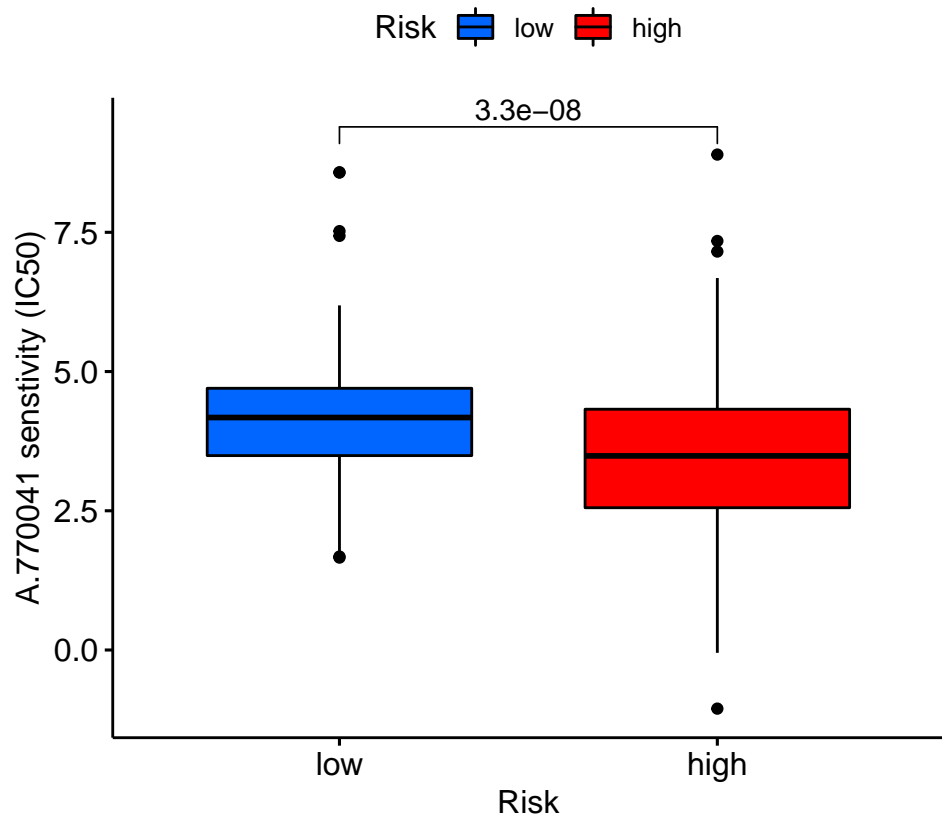

Supplement: Supplementary file 2 [file Presentation1.zip › durgSenstivity.A.770041.pdf]

Risk 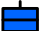 low 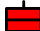 high

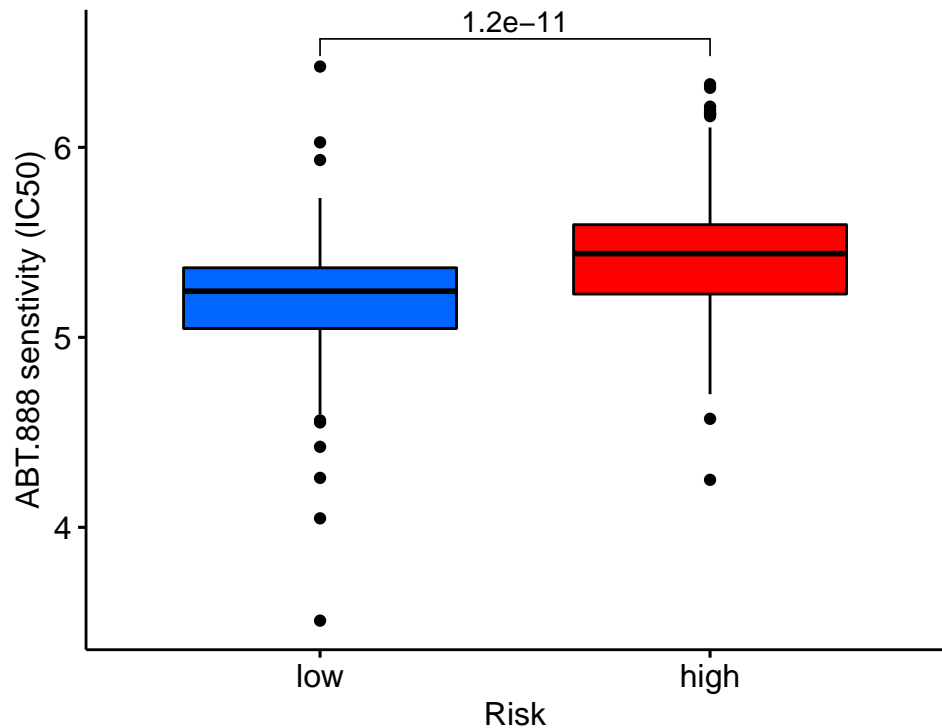

Supplement: Supplementary file 2 [file Presentation1.zip › durgSenstivity.ABT.888.pdf]

Risk 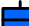 low 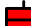 high

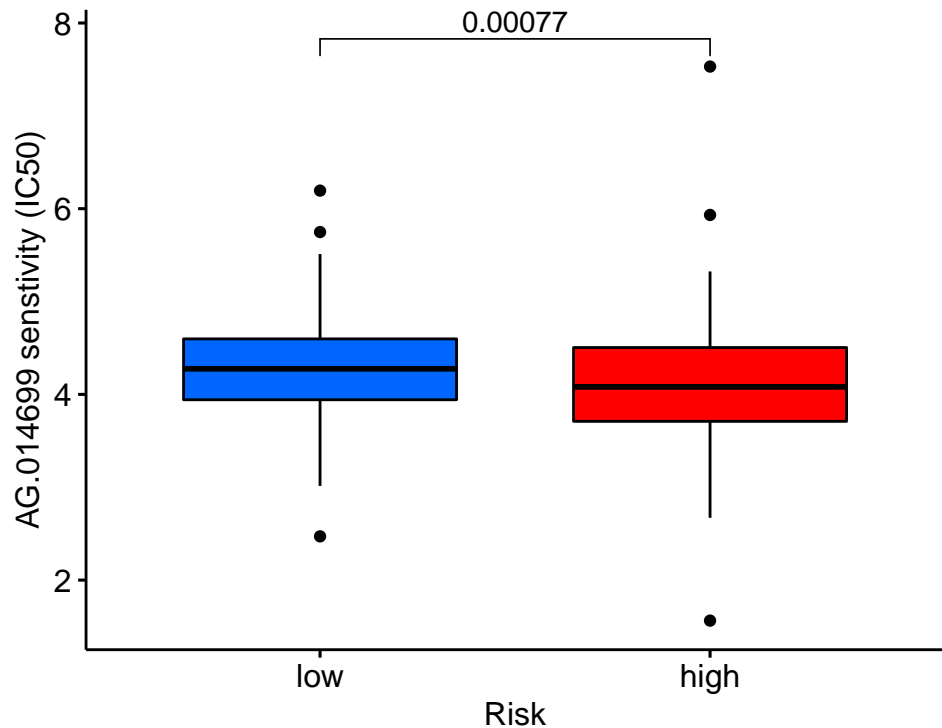

Supplement: Supplementary file 2 [file Presentation1.zip › durgSenstivity.AG.014699.pdf]

Risk 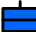 low 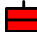 high

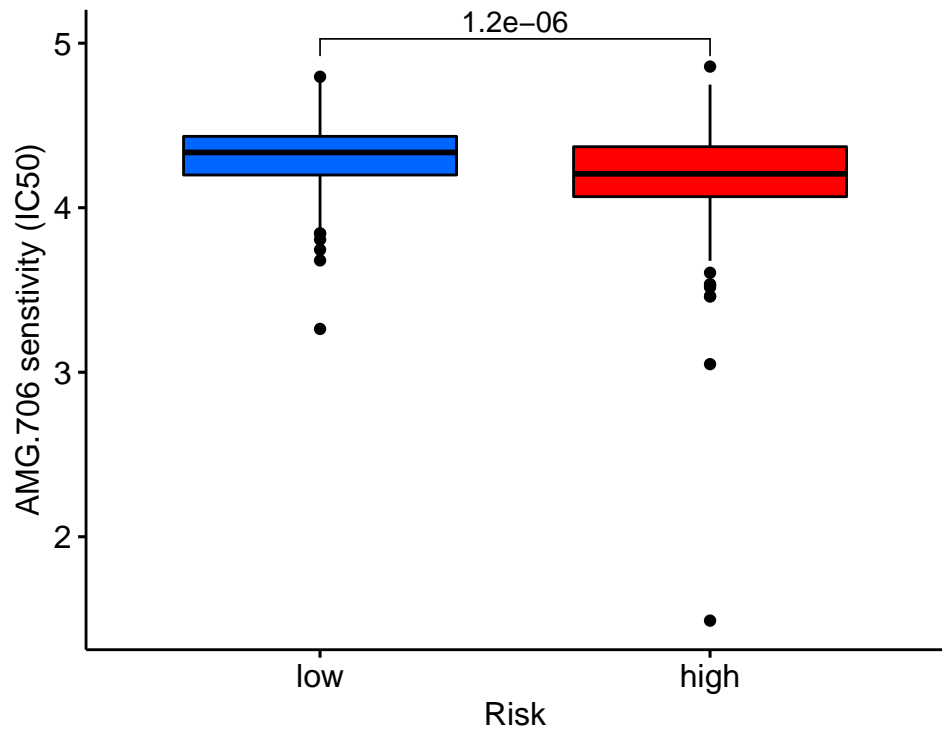

Supplement: Supplementary file 2 [file Presentation1.zip › durgSenstivity.AMG.706.pdf]

Risk 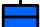 low 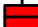 high

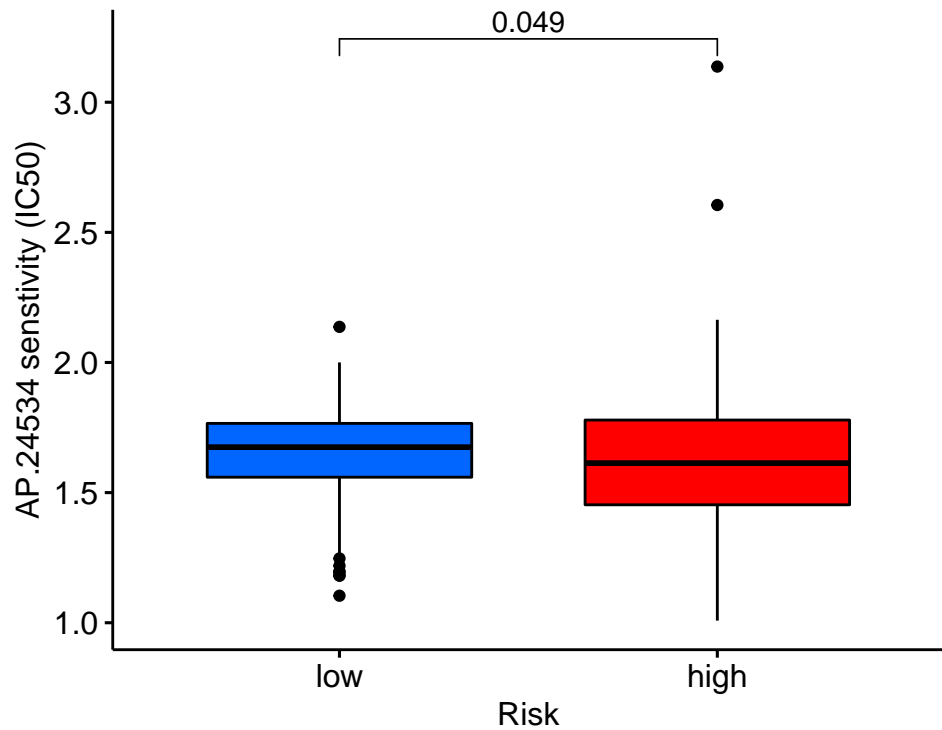

Supplement: Supplementary file 2 [file Presentation1.zip › durgSenstivity.AP.24534.pdf]

Risk 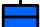 low 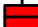 high

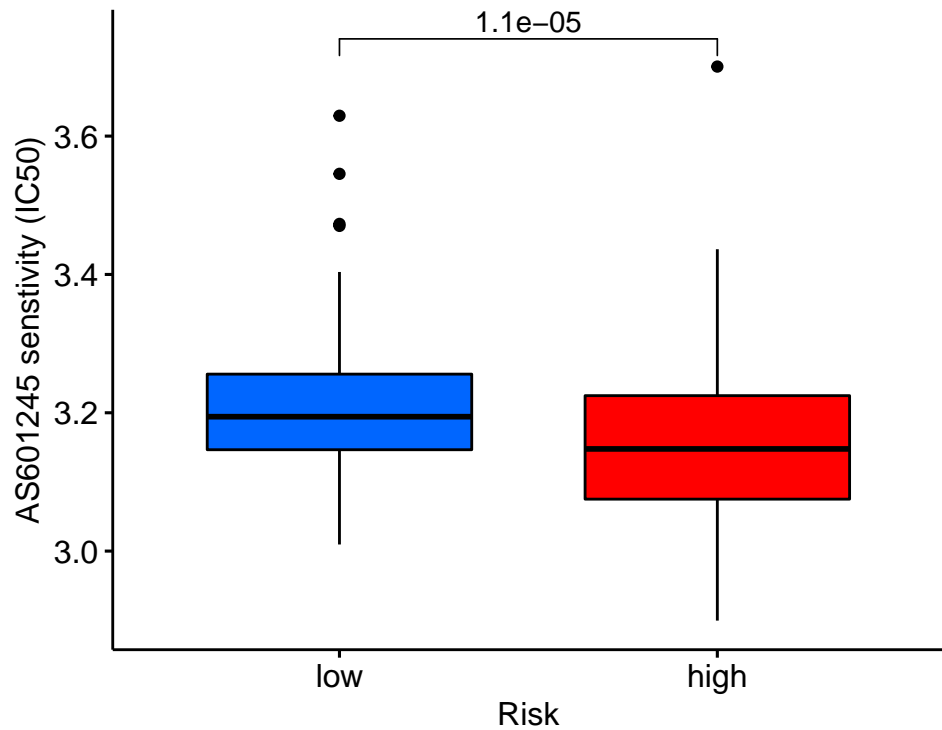

Supplement: Supplementary file 2 [file Presentation1.zip › durgSenstivity.AS601245.pdf]

Risk 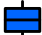 low 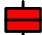 high

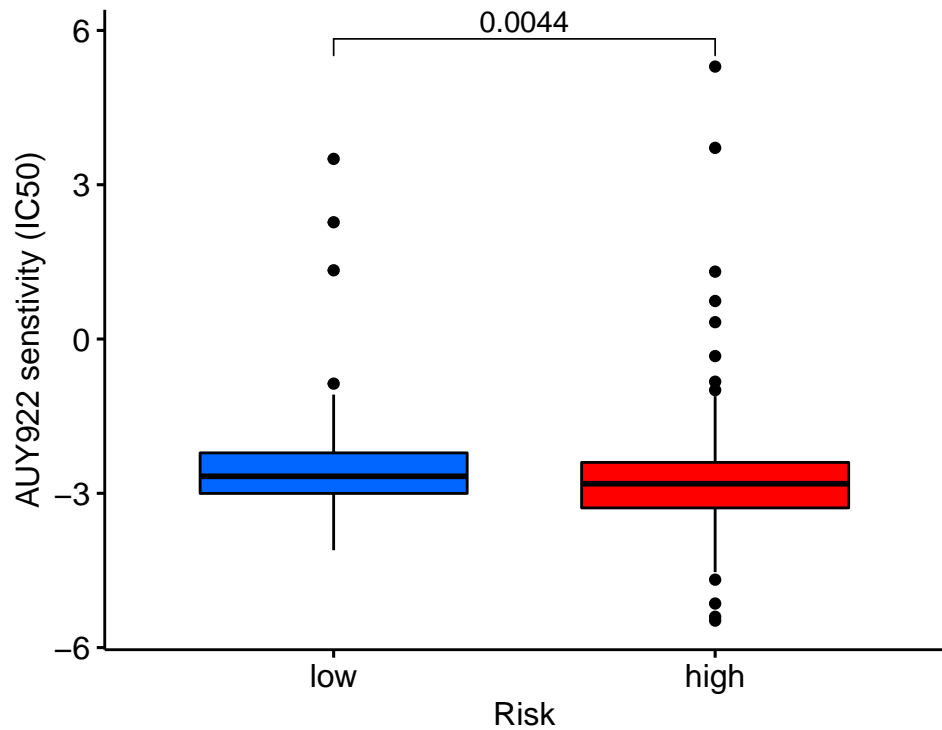

Supplement: Supplementary file 2 [file Presentation1.zip › durgSenstivity.AUY922.pdf]

Risk 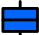 low 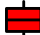 high

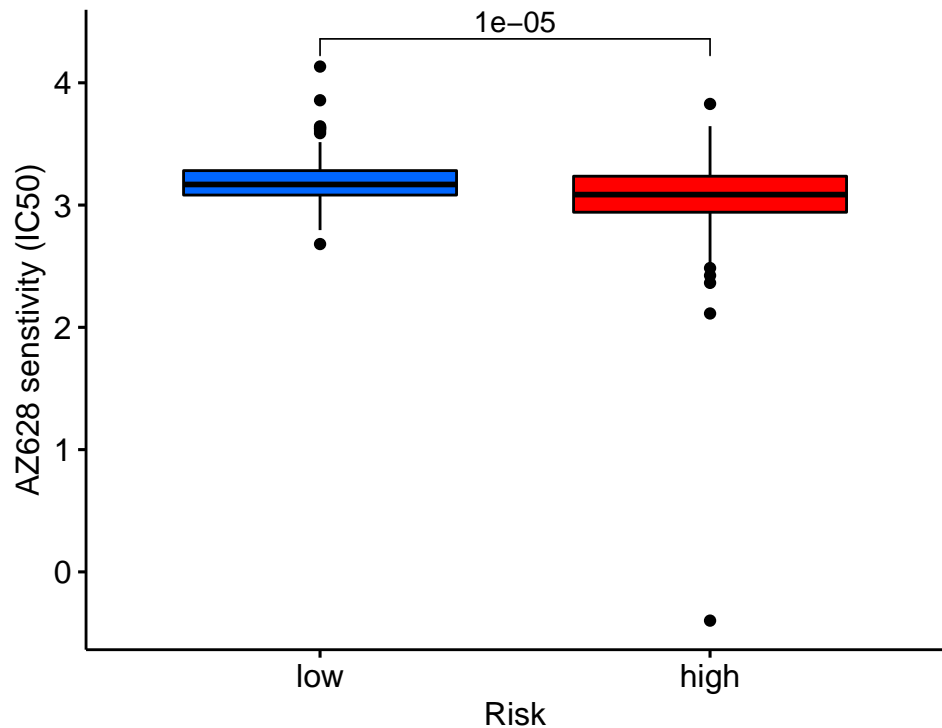

Supplement: Supplementary file 2 [file Presentation1.zip › durgSenstivity.AZ628.pdf]

Risk low high

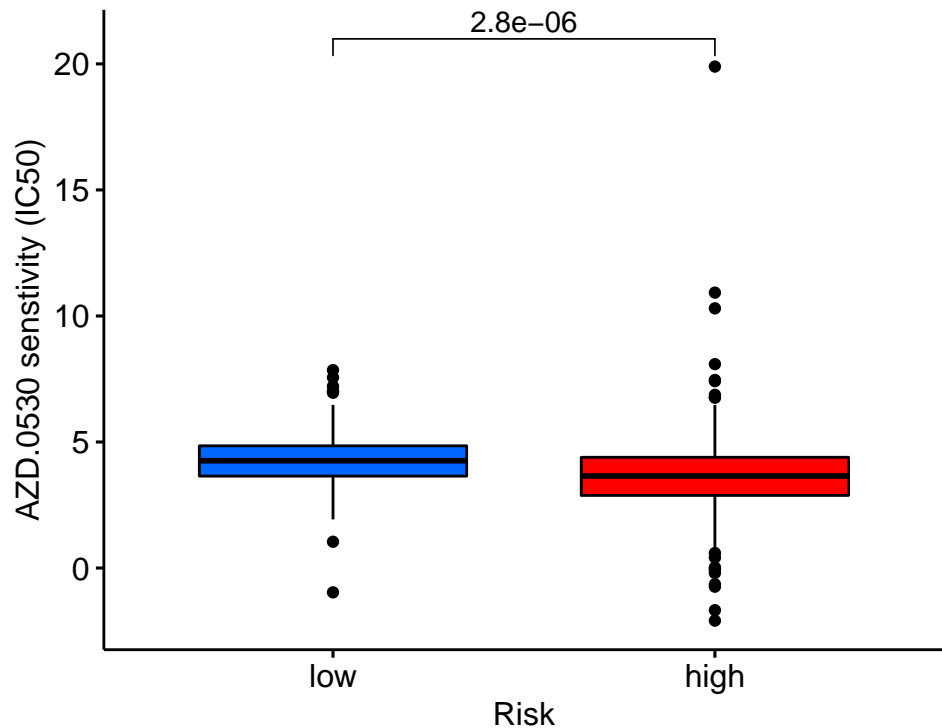

Supplement: Supplementary file 2 [file Presentation1.zip › durgSenstivity.AZD.0530.pdf]

AZD8055 sensitivity (IC50)

Risk 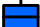 low 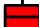 high

$7.4e-05$

low

high

Risk

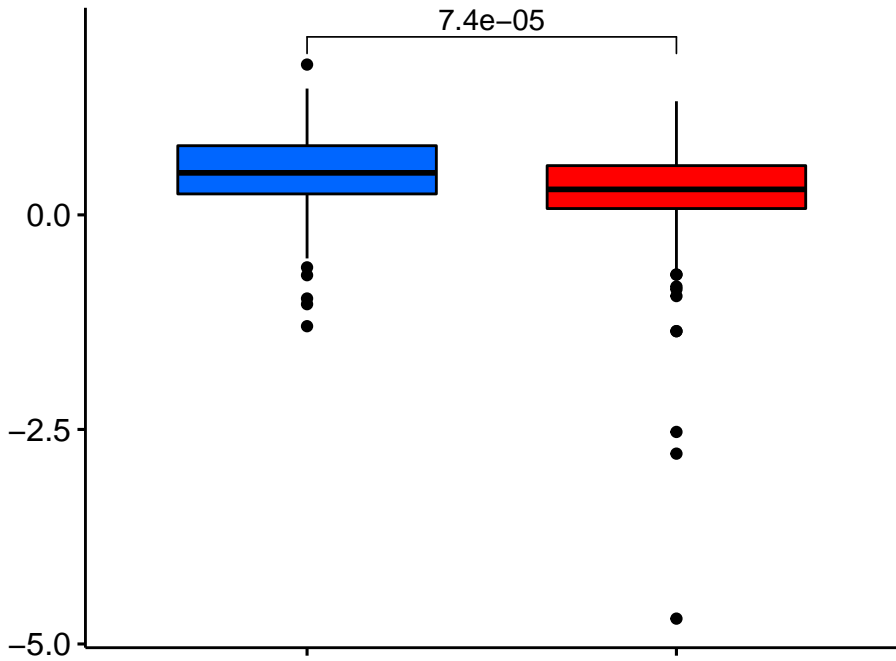

Supplement: Supplementary file 2 [file Presentation1.zip › durgSenstivity.AZD8055.pdf]

Risk 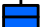 low 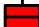 high

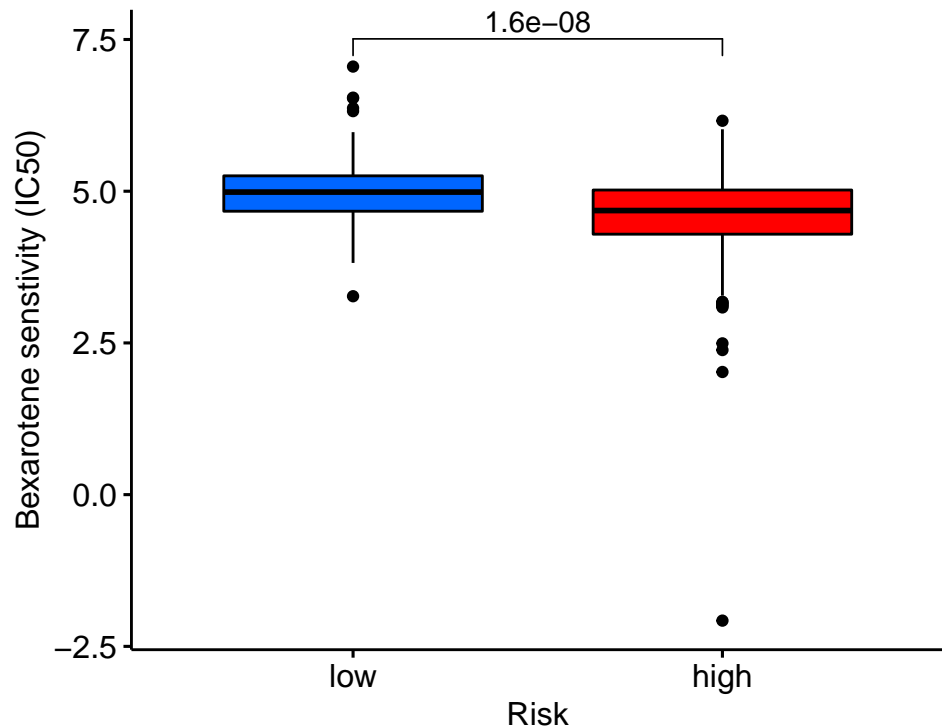

Supplement: Supplementary file 2 [file Presentation1.zip › durgSenstivity.Bexarotene.pdf]

Risk 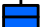 low 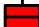 high

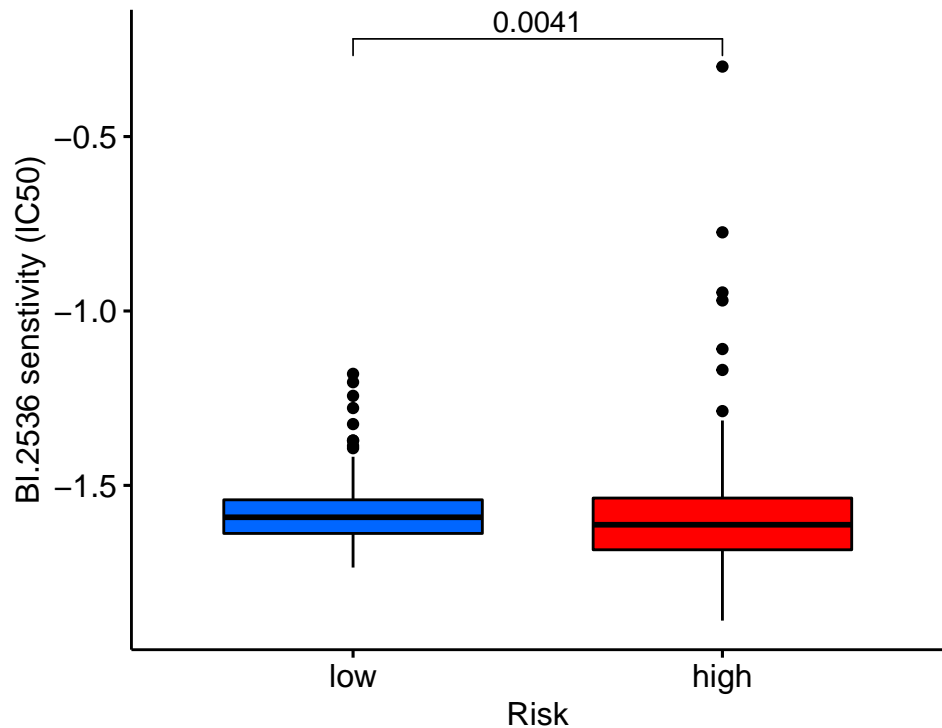

Supplement: Supplementary file 2 [file Presentation1.zip › durgSenstivity.BI.2536.pdf]

Risk 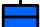 low 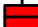 high

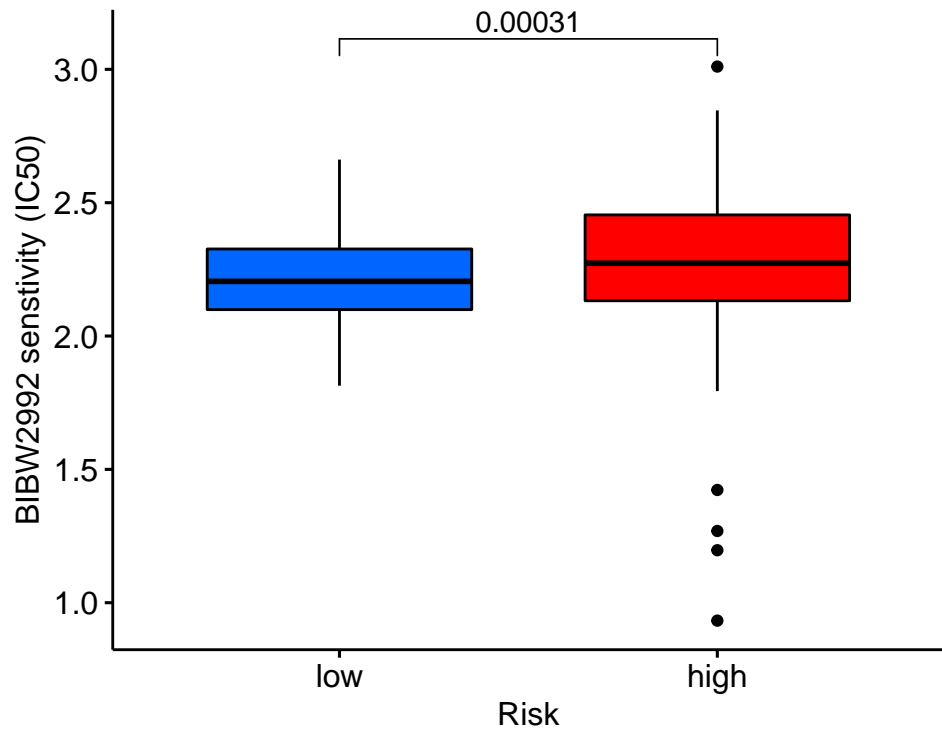

Supplement: Supplementary file 2 [file Presentation1.zip › durgSenstivity.BIBW2992.pdf]

Risk 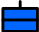 low 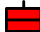 high

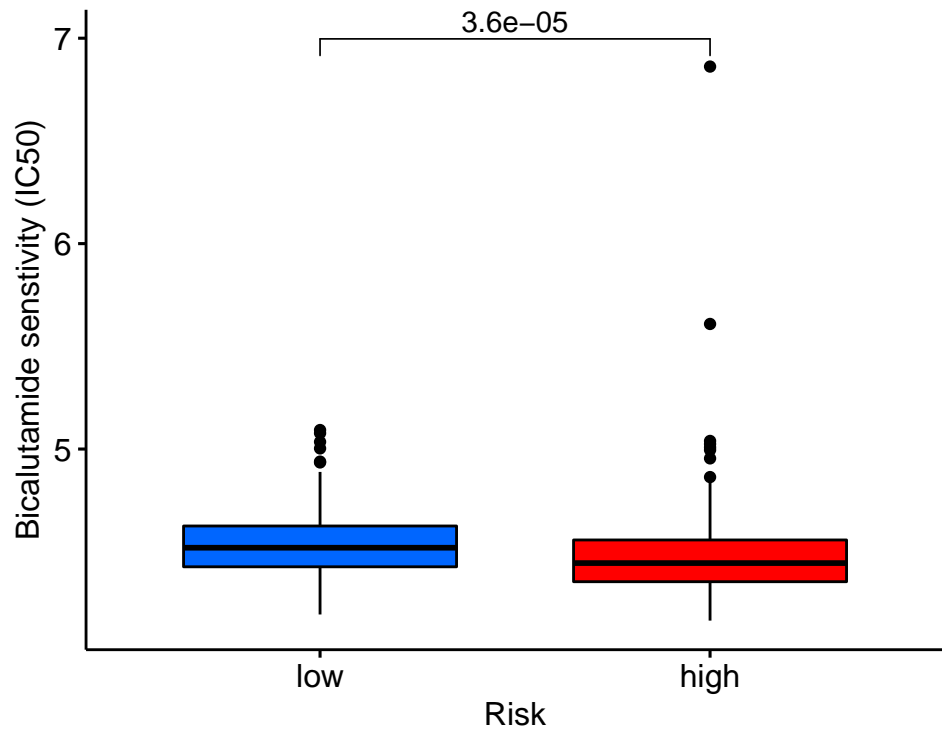

Supplement: Supplementary file 2 [file Presentation1.zip › durgSenstivity.Bicalutamide.pdf]

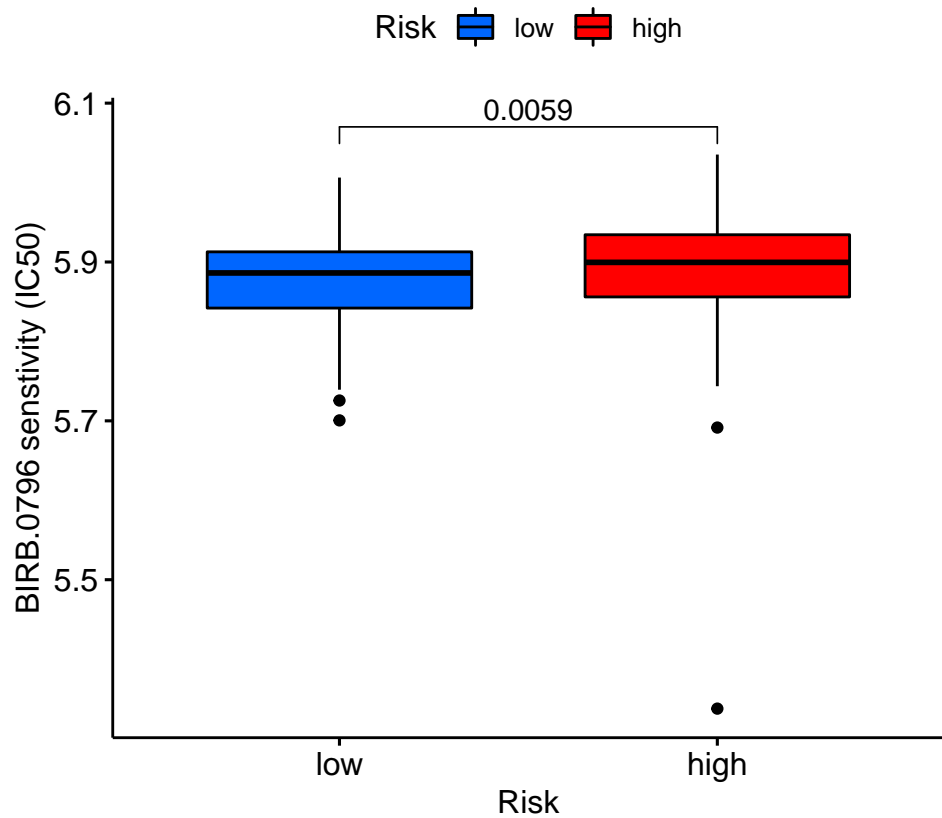

Supplement: Supplementary file 2 [file Presentation1.zip › durgSenstivity.BIRB.0796.pdf]

Risk 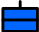 low 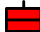 high

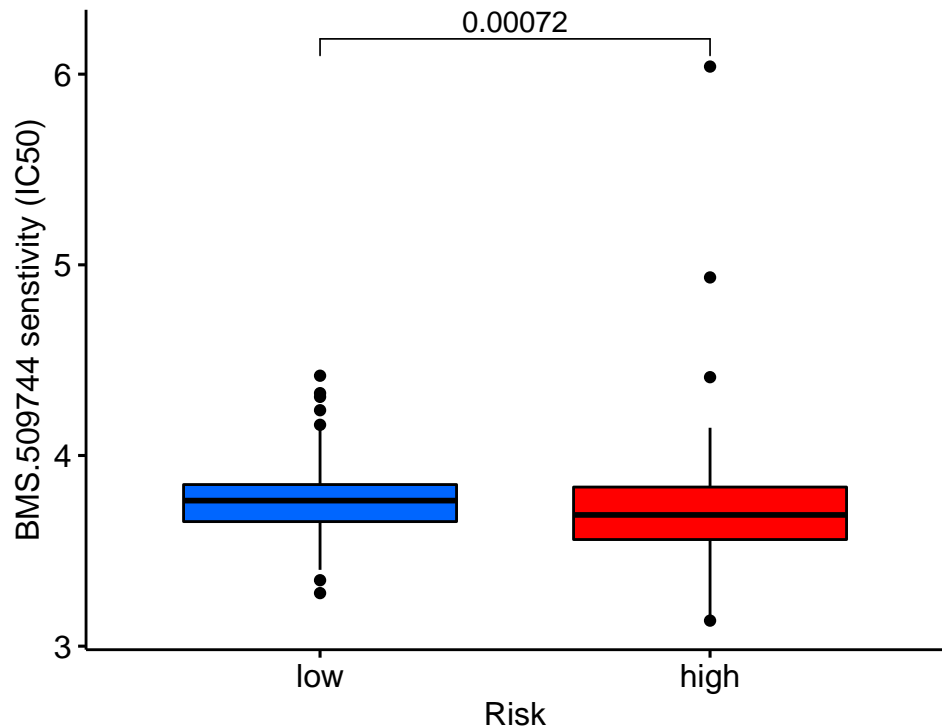

Supplement: Supplementary file 2 [file Presentation1.zip › durgSenstivity.BMS.509744.pdf]

Risk low high

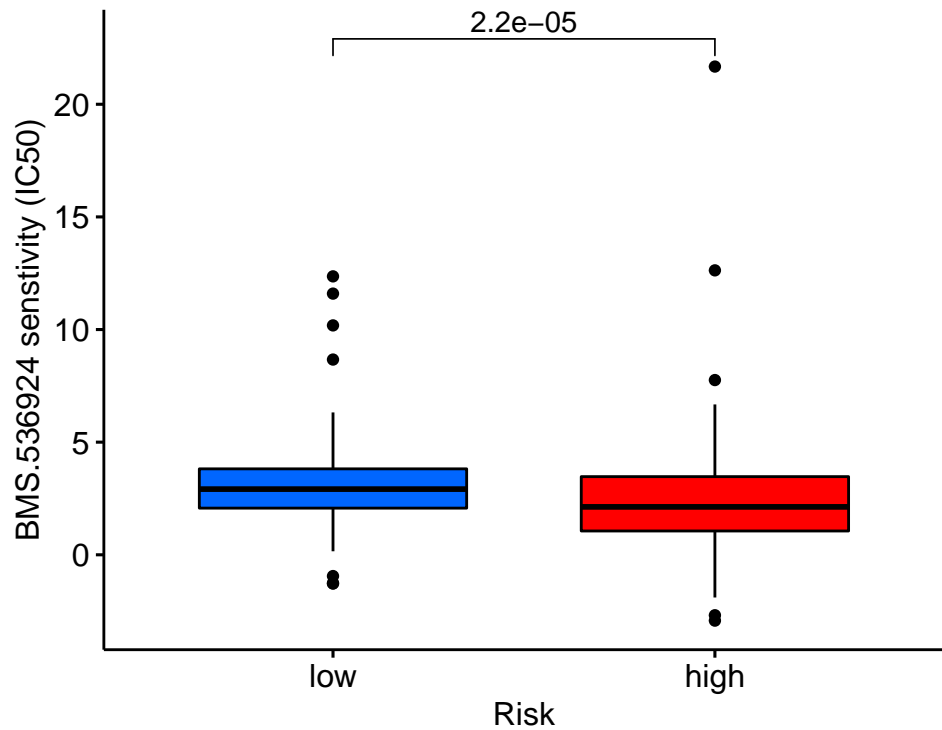

Supplement: Supplementary file 2 [file Presentation1.zip › durgSenstivity.BMS.536924.pdf]

BMS.754807 sensitivity (IC50)

Risk low high

0.04

low

high

Risk

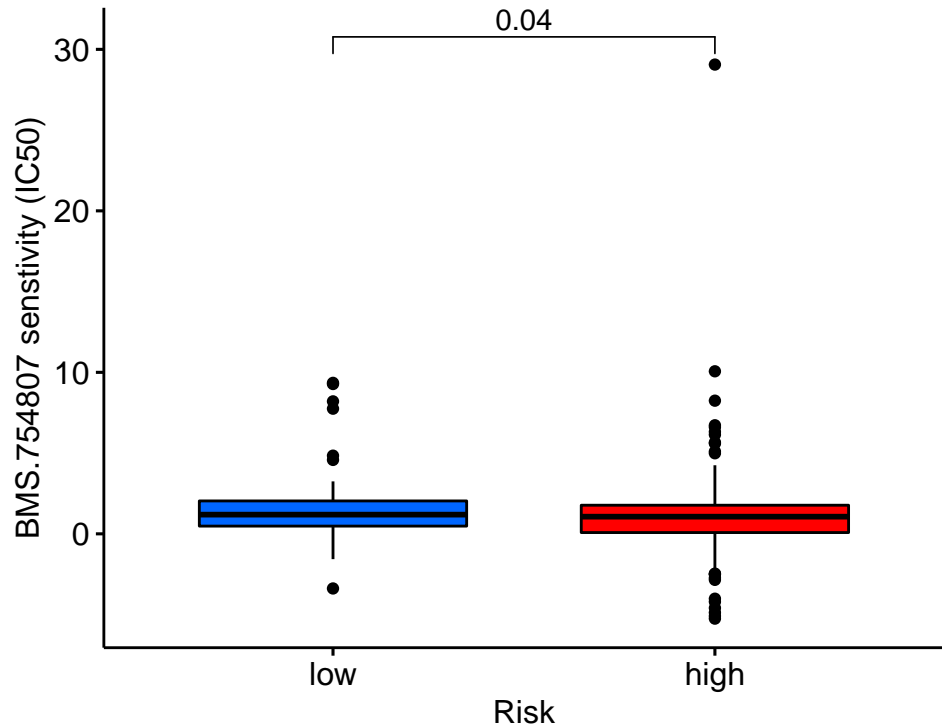

Supplement: Supplementary file 2 [file Presentation1.zip › durgSenstivity.BMS.754807.pdf]

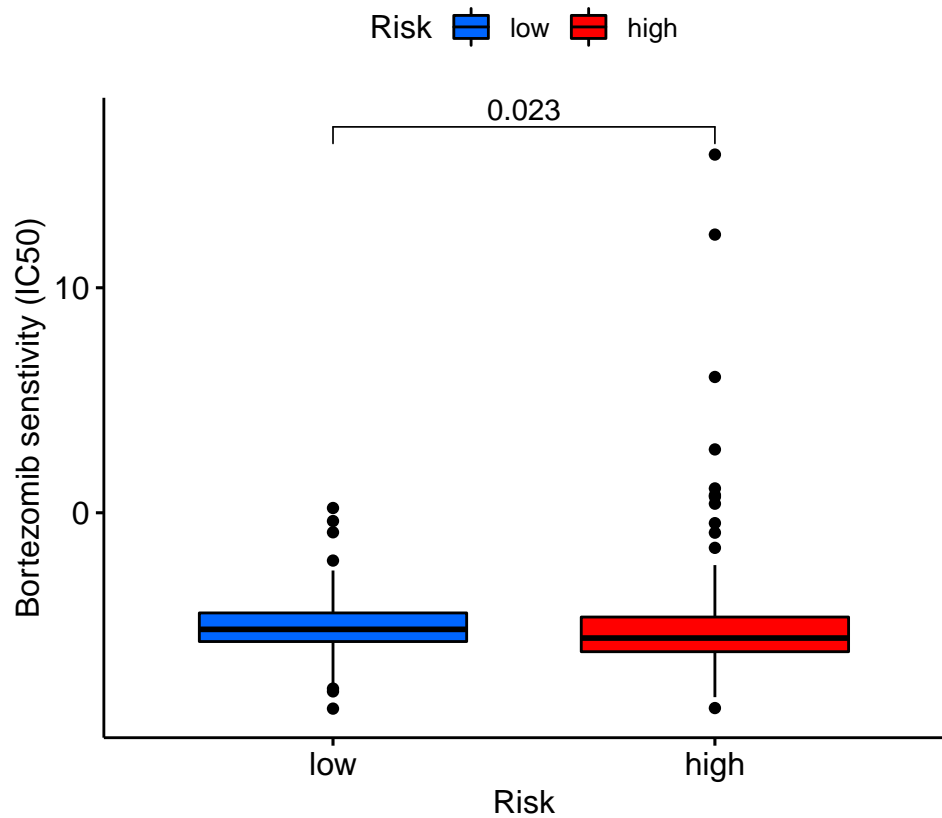

Supplement: Supplementary file 2 [file Presentation1.zip › durgSenstivity.Bortezomib.pdf]

Risk 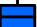 low 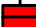 high

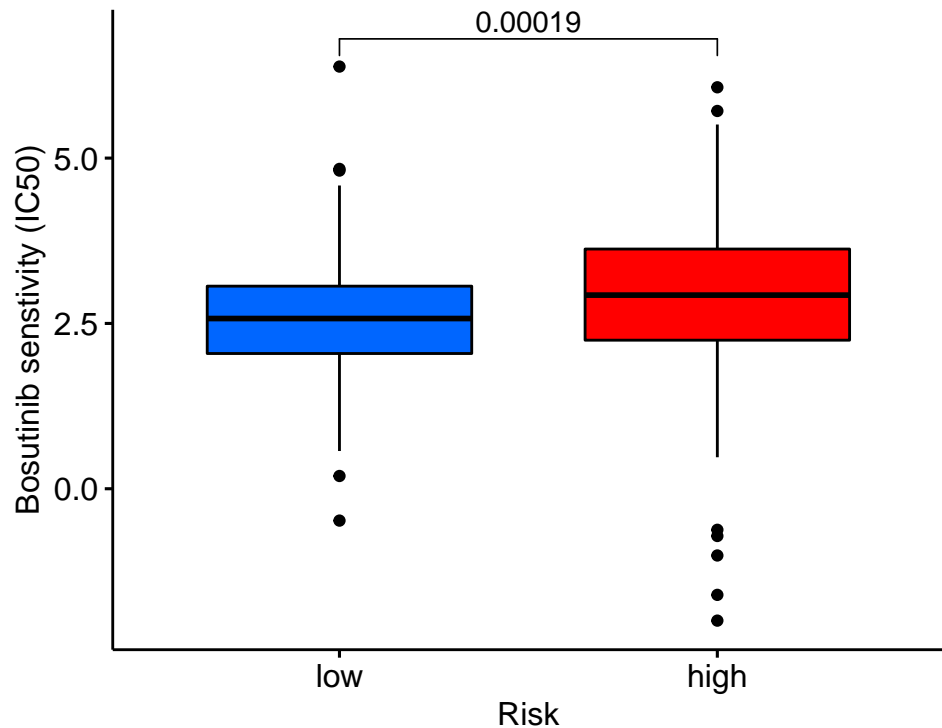

Supplement: Supplementary file 2 [file Presentation1.zip › durgSenstivity.Bosutinib.pdf]

Bryostat1 sensitivity (IC50)

Risk 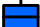 low 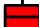 high

1.1e-11

low

high

Risk

0.0  
-0.5  
-1.0  
-1.5

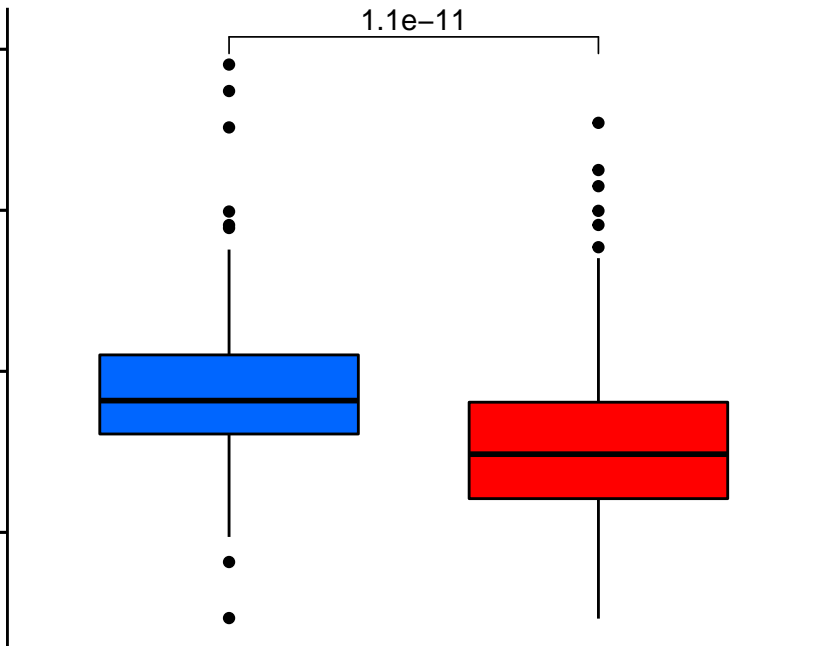

Supplement: Supplementary file 2 [file Presentation1.zip › durgSenstivity.Bryostatin.1.pdf]

Risk 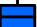 low 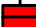 high

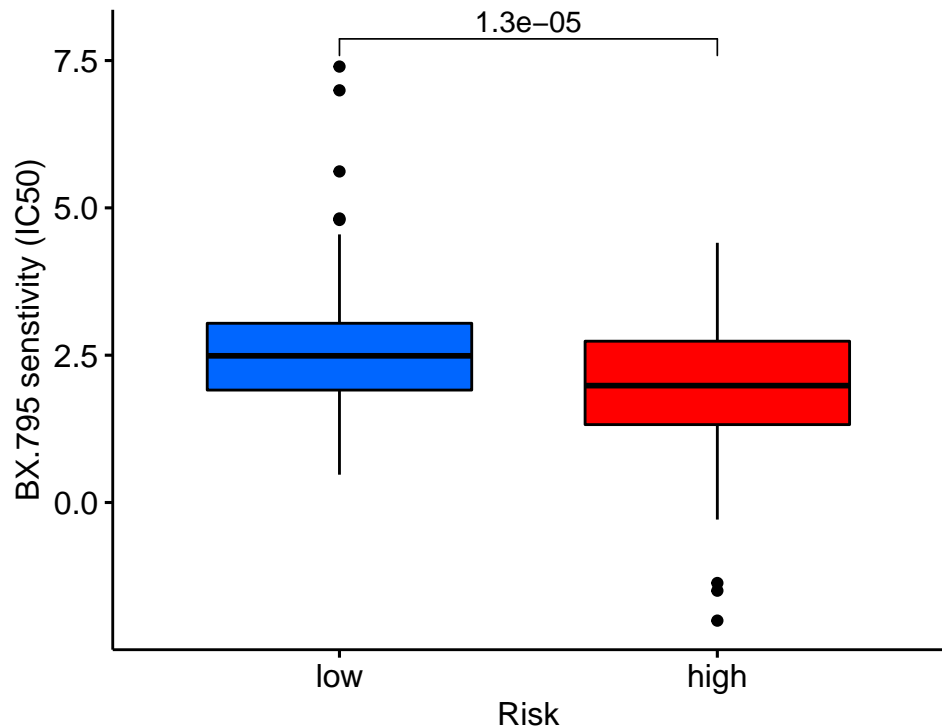

Supplement: Supplementary file 2 [file Presentation1.zip › durgSenstivity.BX.795.pdf]

Camptothecin sensitivity (IC50)

Risk 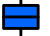 low 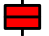 high

0.00068

low

high

Risk

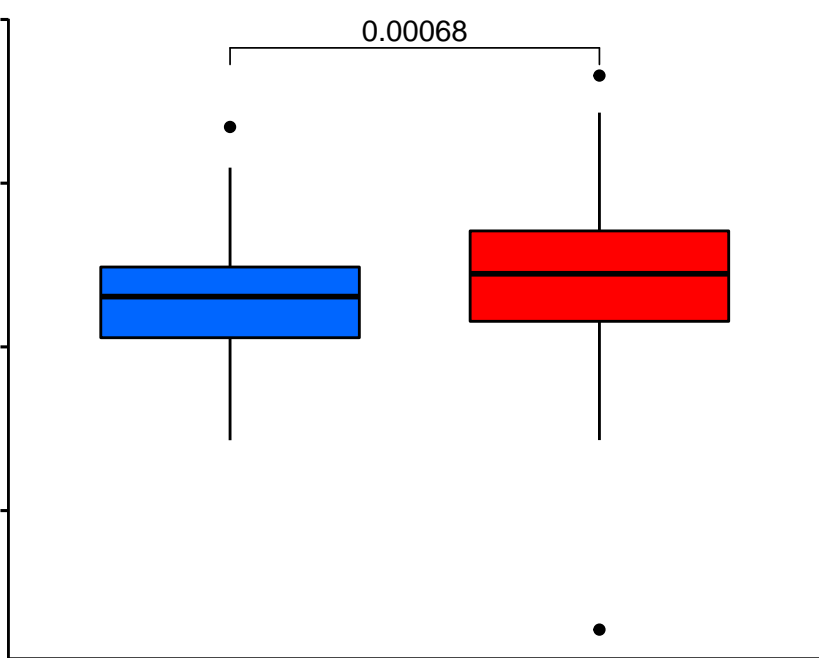

Supplement: Supplementary file 2 [file Presentation1.zip › durgSenstivity.Camptothecin.pdf]

Risk 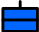 low 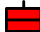 high

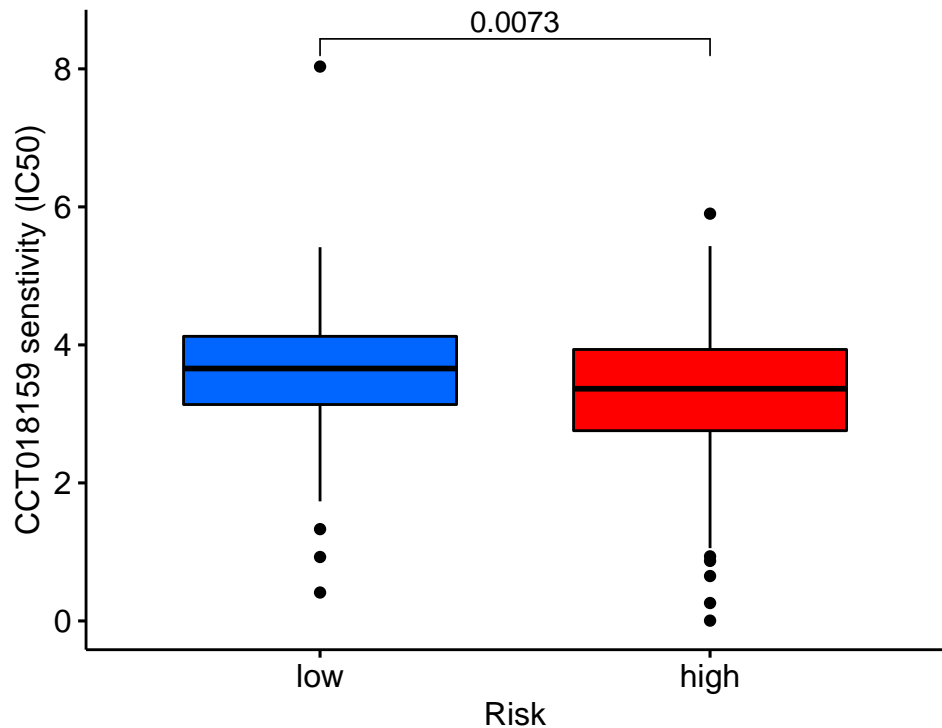

Supplement: Supplementary file 2 [file Presentation1.zip › durgSenstivity.CCT018159.pdf]

CGP.60474 sensitivity (IC50)

Risk 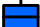 low 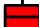 high

$8.9\text{e-}08$

low

high

Risk

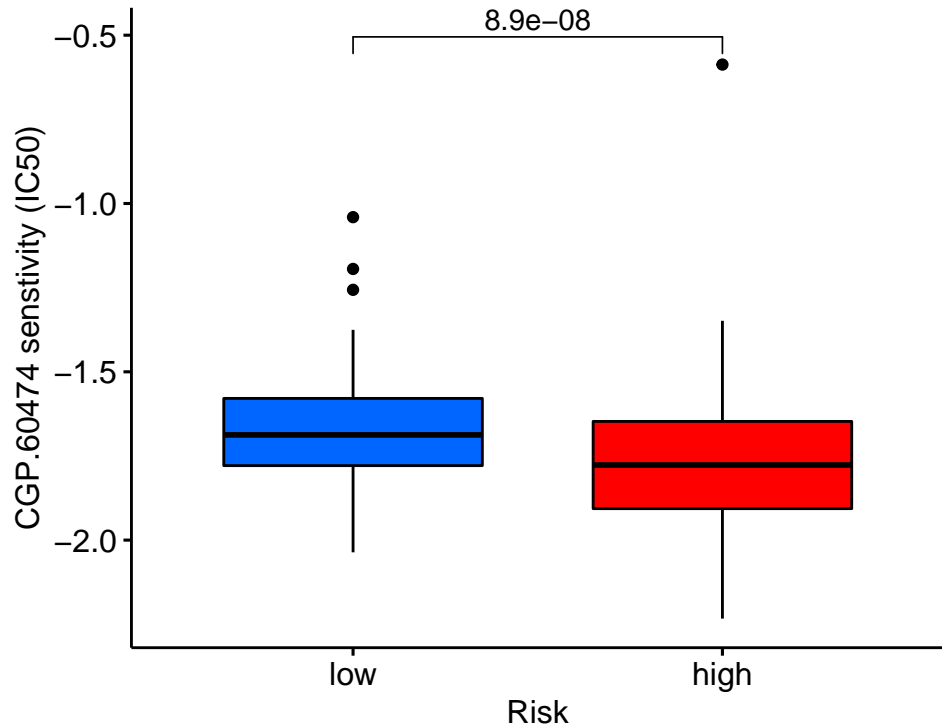

Supplement: Supplementary file 2 [file Presentation1.zip › durgSenstivity.CGP.60474.pdf]

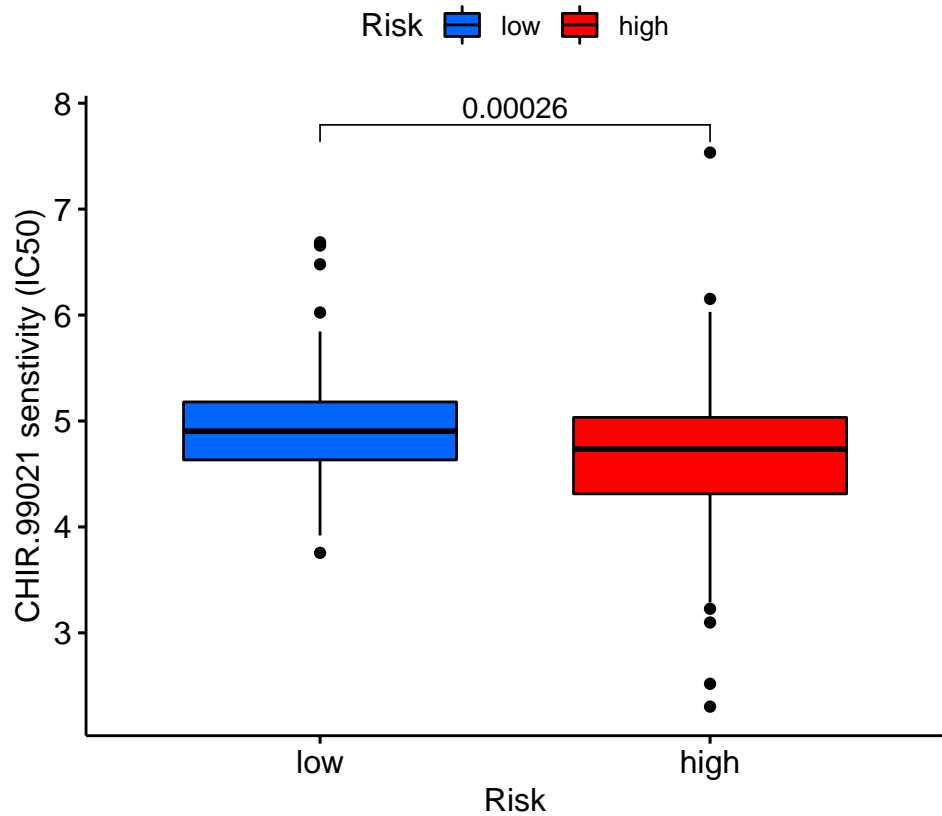

Supplement: Supplementary file 2 [file Presentation1.zip › durgSenstivity.CHIR.99021.pdf]

Risk 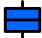 low 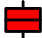 high

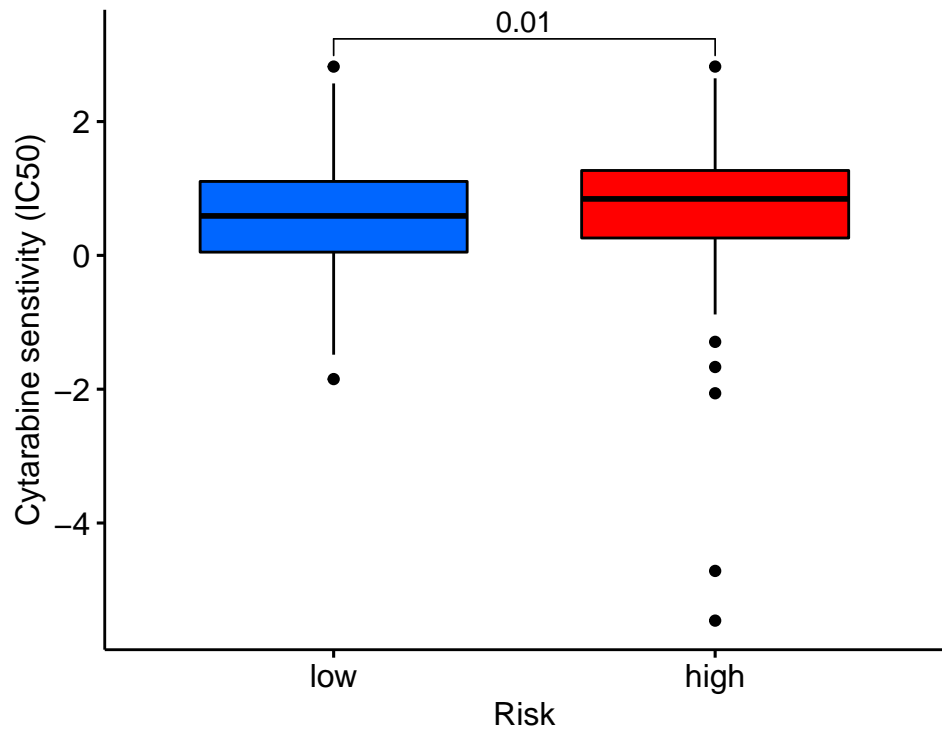

Supplement: Supplementary file 2 [file Presentation1.zip › durgSenstivity.Cytarabine.pdf]

Risk low high

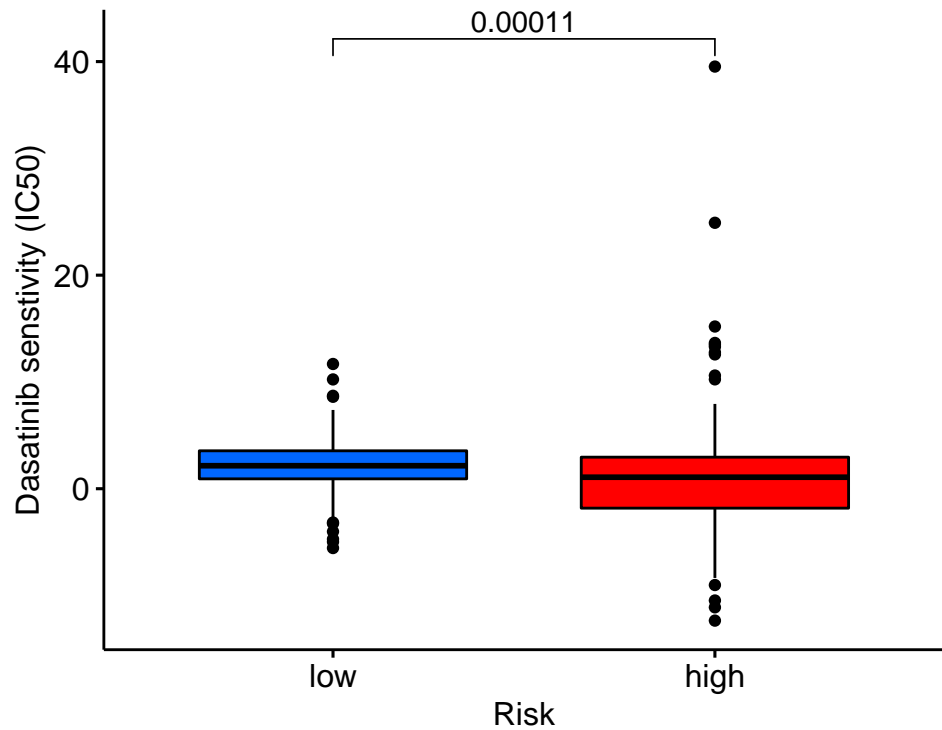

Supplement: Supplementary file 2 [file Presentation1.zip › durgSenstivity.Dasatinib.pdf]

Risk low high

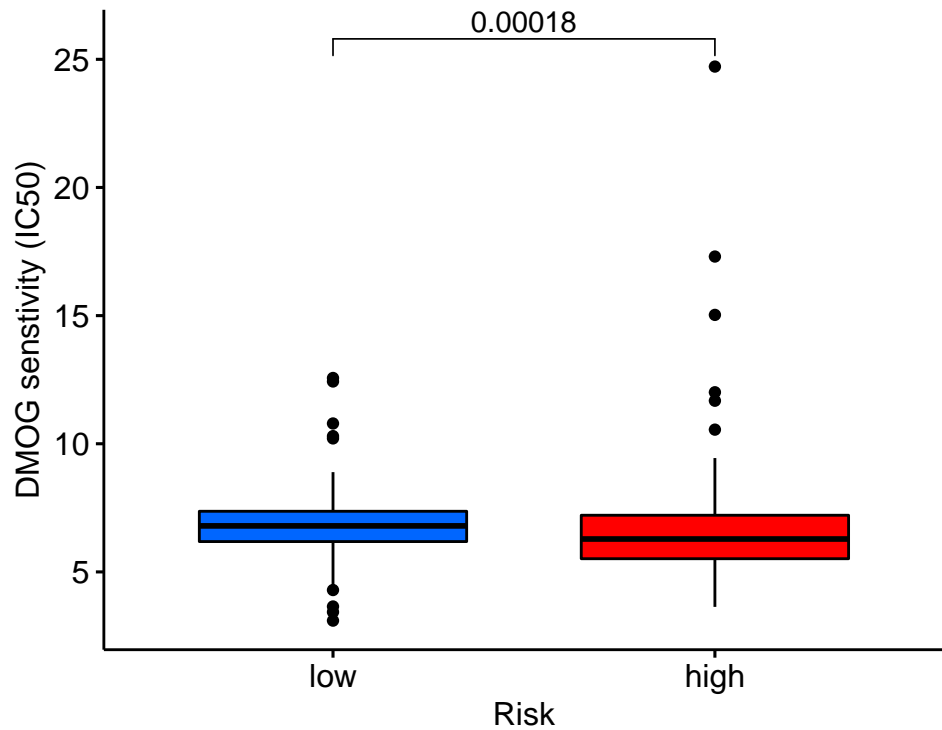

Supplement: Supplementary file 2 [file Presentation1.zip › durgSenstivity.DMOG.pdf]

Risk 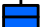 low 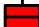 high

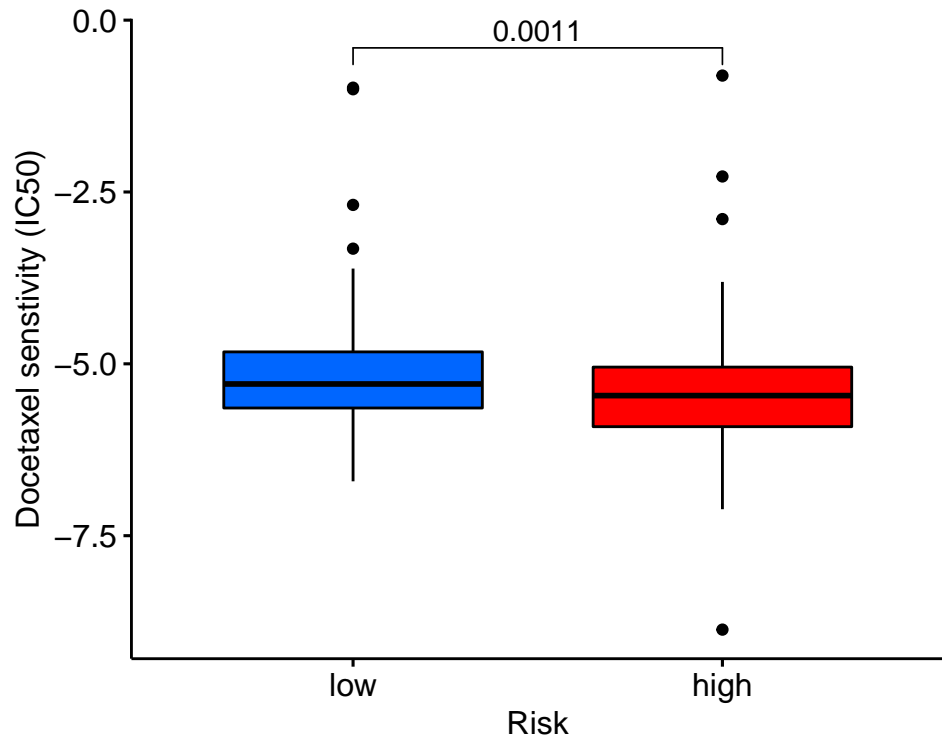

Supplement: Supplementary file 2 [file Presentation1.zip › durgSenstivity.Docetaxel.pdf]

Risk 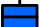 low 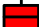 high

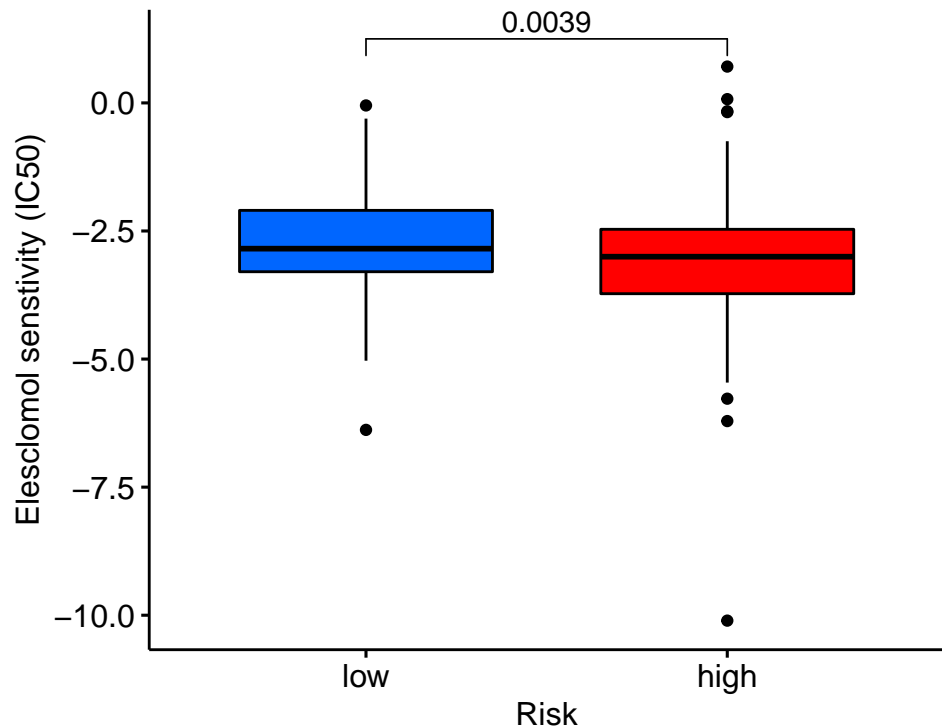

Supplement: Supplementary file 2 [file Presentation1.zip › durgSenstivity.Elesclomol.pdf]

Risk 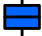 low 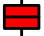 high

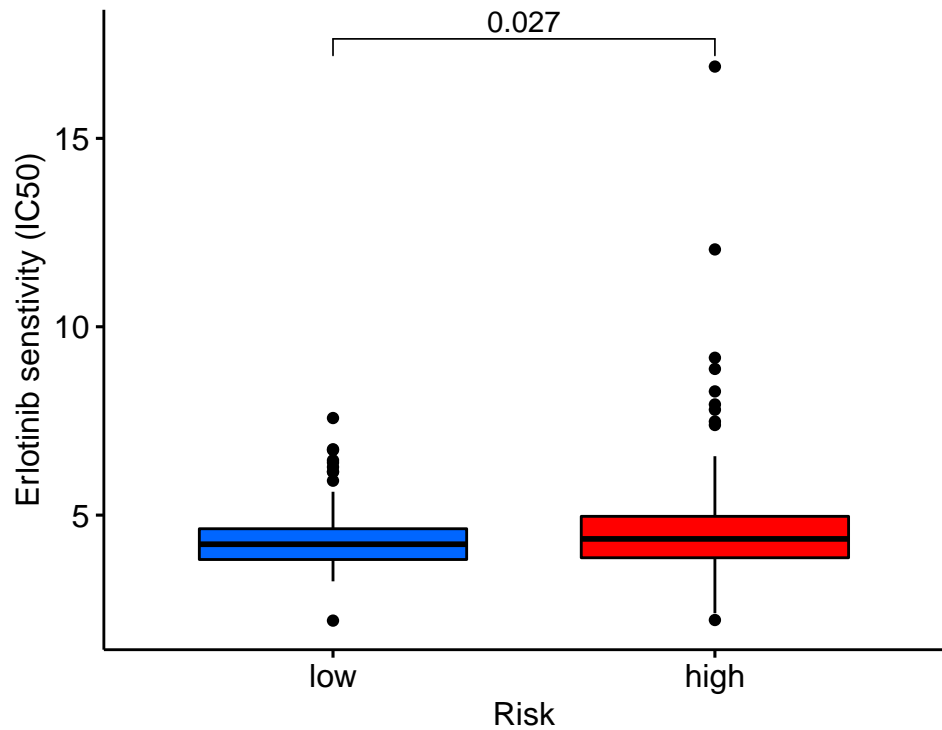

Supplement: Supplementary file 2 [file Presentation1.zip › durgSenstivity.Erlotinib.pdf]

Risk low high

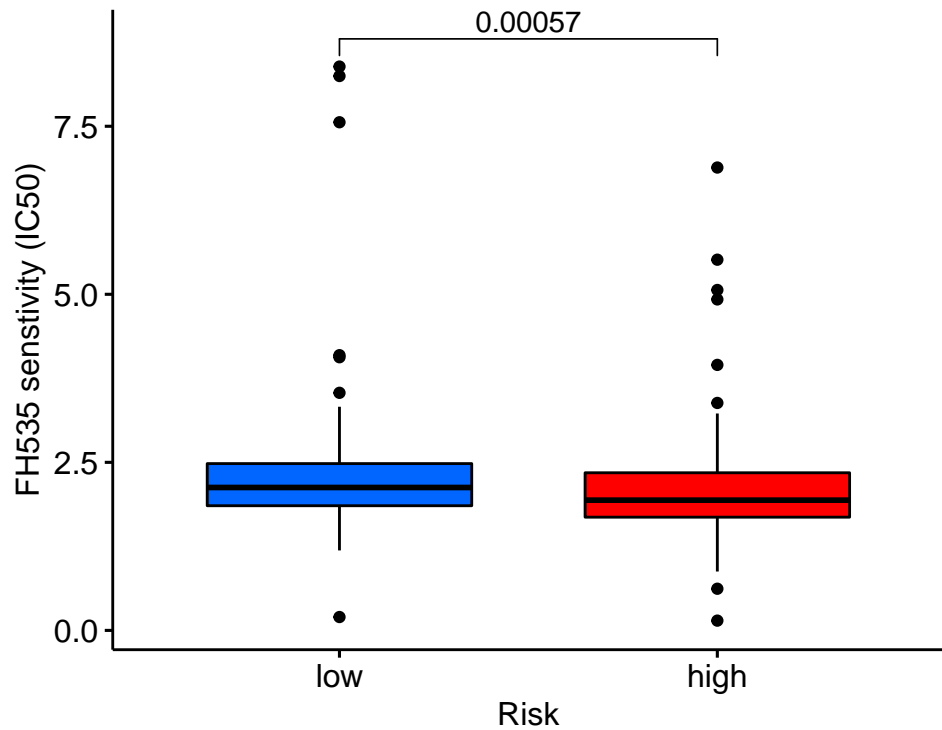

Supplement: Supplementary file 2 [file Presentation1.zip › durgSenstivity.FH535.pdf]

Risk 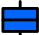 low 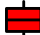 high

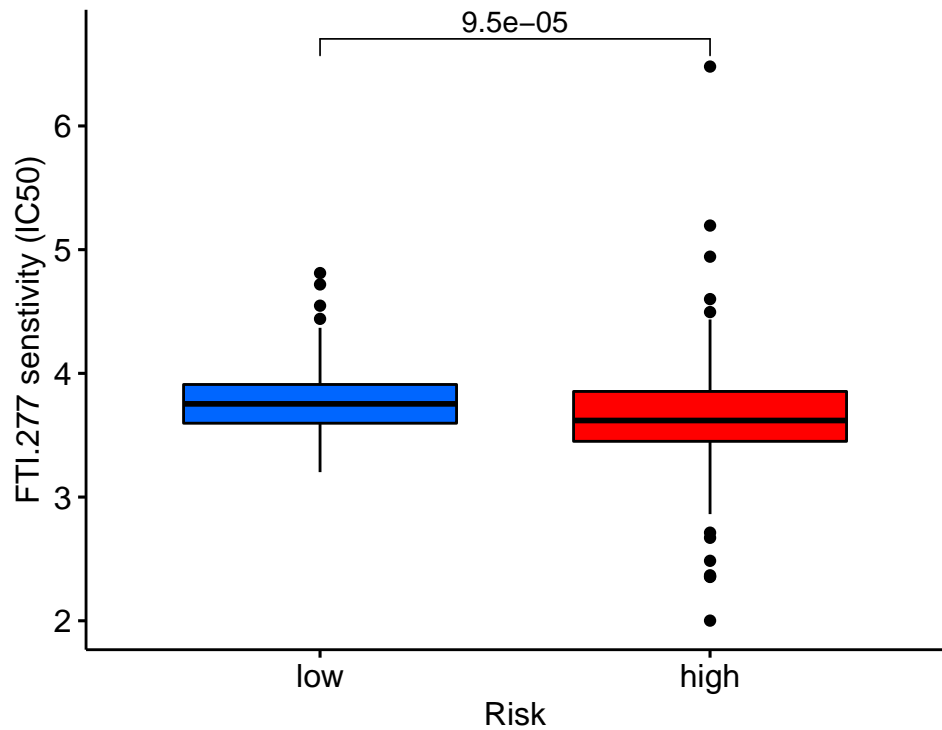

Supplement: Supplementary file 2 [file Presentation1.zip › durgSenstivity.FTI.277.pdf]

Risk 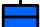 low 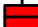 high

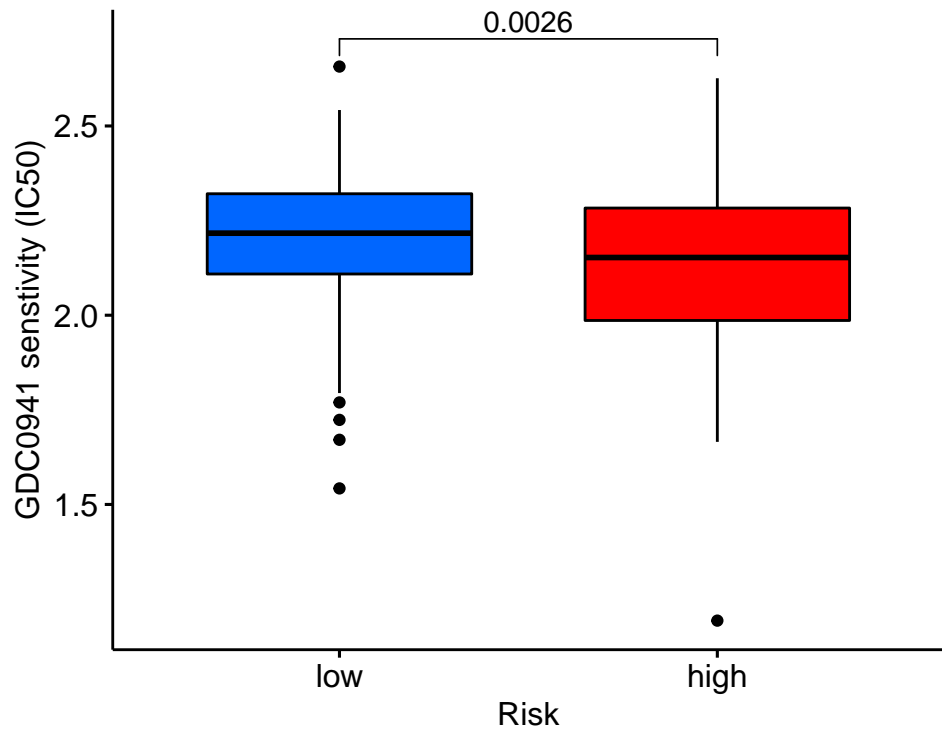

Supplement: Supplementary file 2 [file Presentation1.zip › durgSenstivity.GDC0941.pdf]

Risk 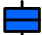 low 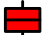 high

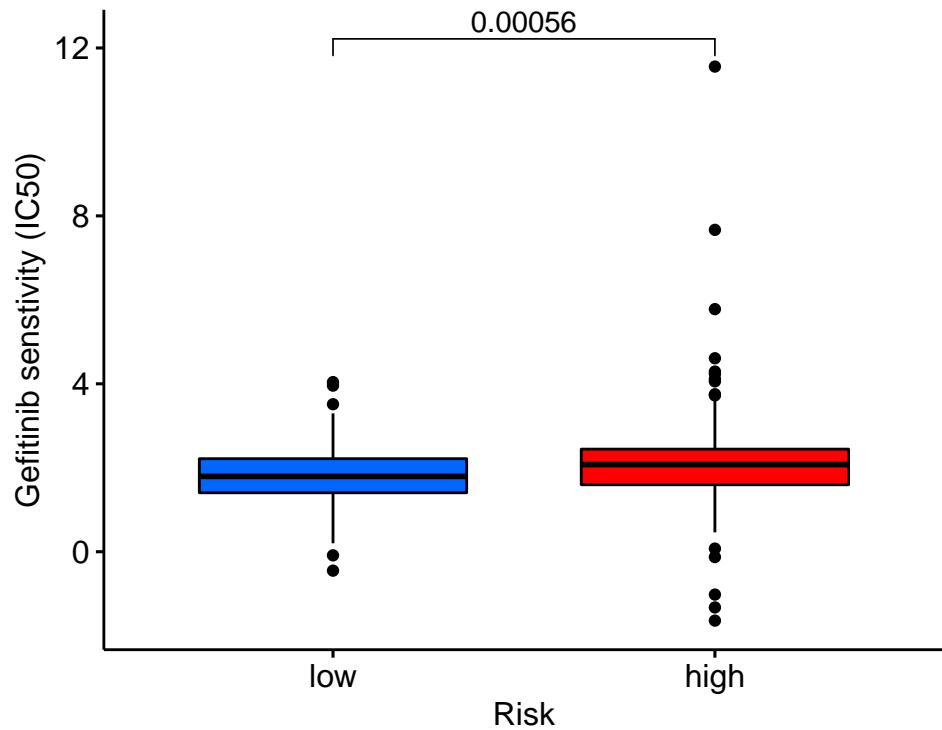

Supplement: Supplementary file 2 [file Presentation1.zip › durgSenstivity.Gefitinib.pdf]

Risk 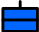 low 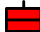 high

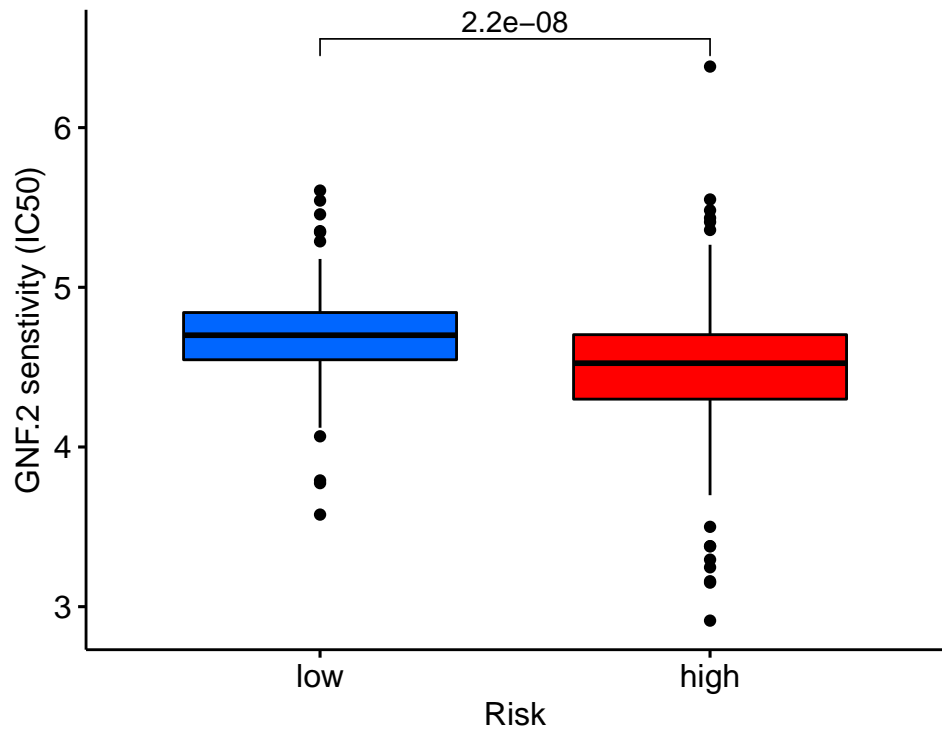

Supplement: Supplementary file 2 [file Presentation1.zip › durgSenstivity.GNF.2.pdf]

Risk 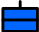 low 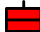 high

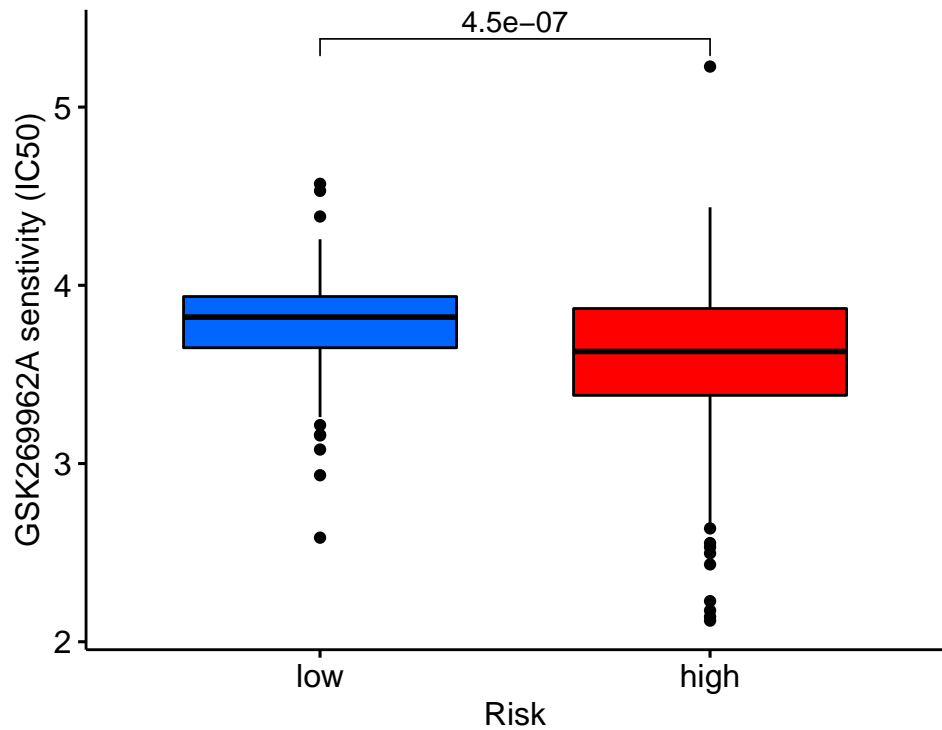

Supplement: Supplementary file 2 [file Presentation1.zip › durgSenstivity.GSK269962A.pdf]

Risk 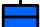 low 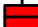 high

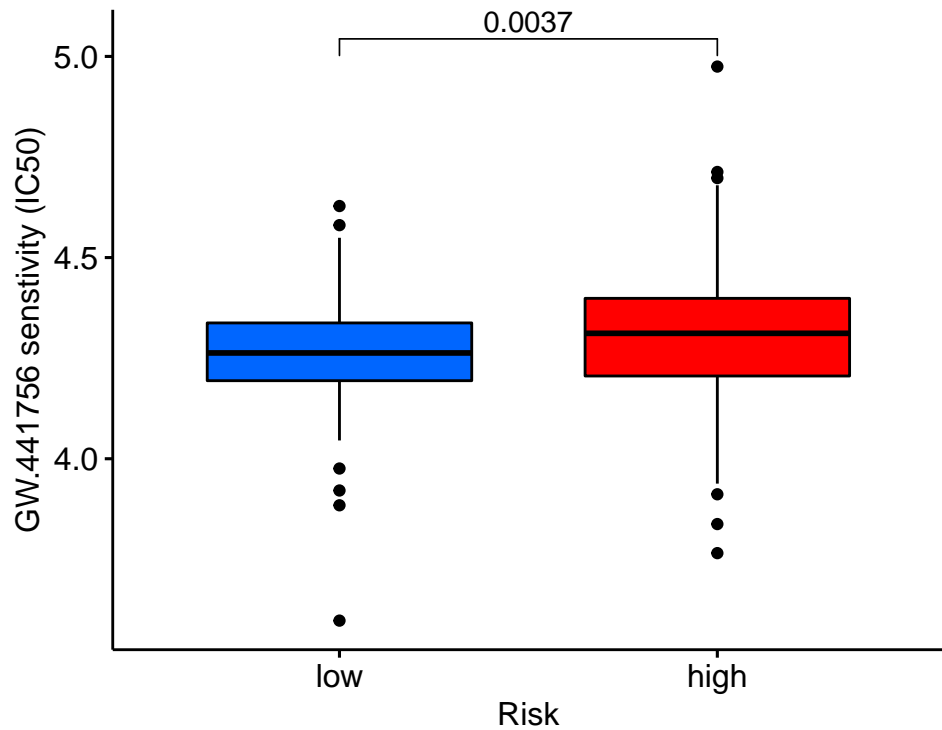

Supplement: Supplementary file 2 [file Presentation1.zip › durgSenstivity.GW.441756.pdf]

Risk 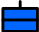 low 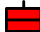 high

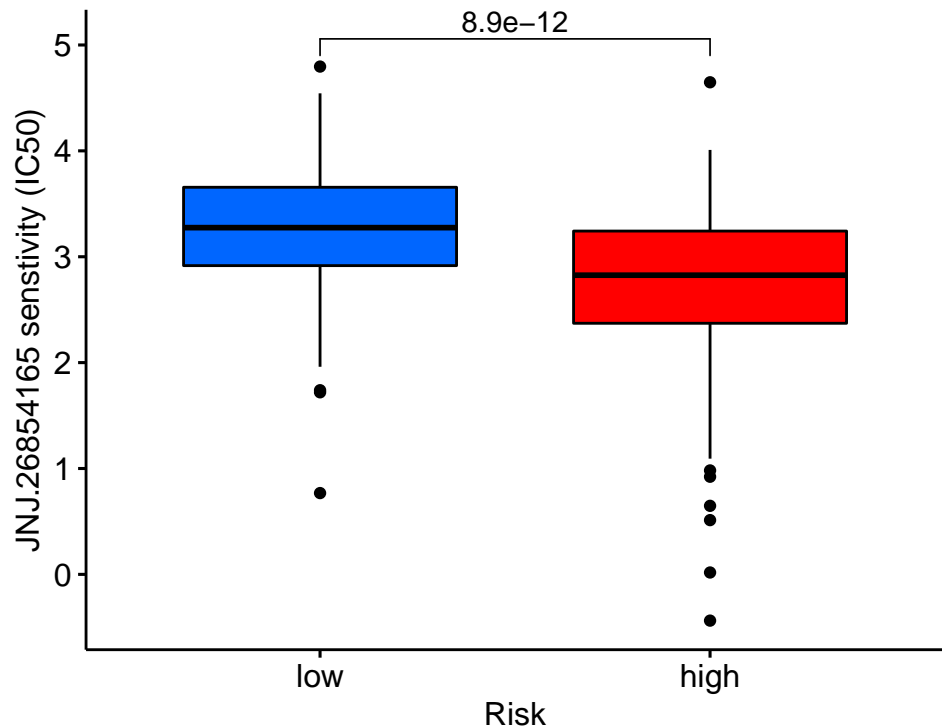

Supplement: Supplementary file 2 [file Presentation1.zip › durgSenstivity.JNJ.26854165.pdf]

JW.7.52.1 sensitivity (IC50)

Risk 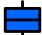 low 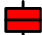 high

$7.5e-07$

low

high

Risk

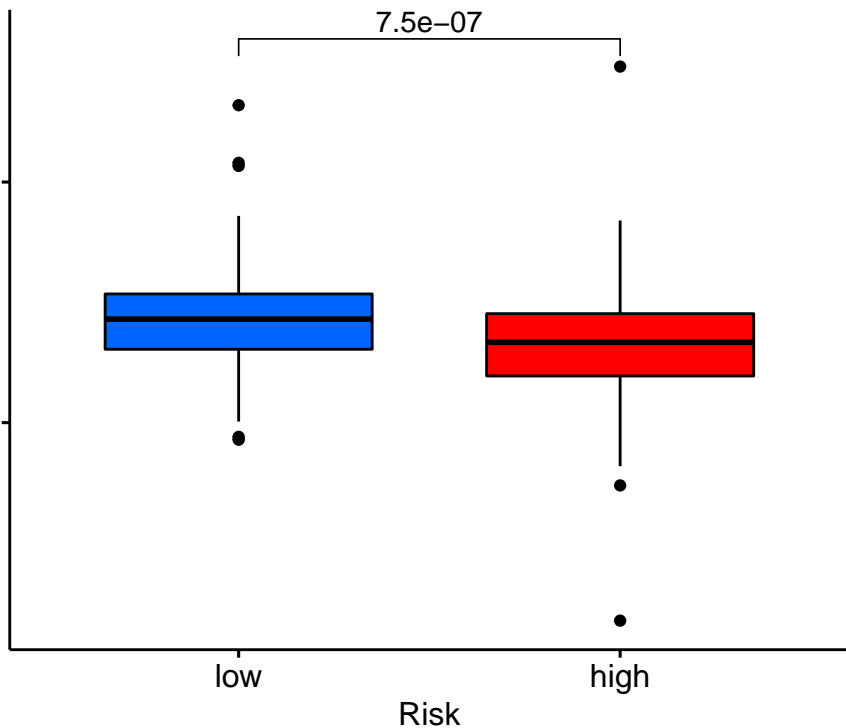

Supplement: Supplementary file 2 [file Presentation1.zip › durgSenstivity.JW.7.52.1.pdf]

Risk 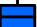 low 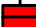 high

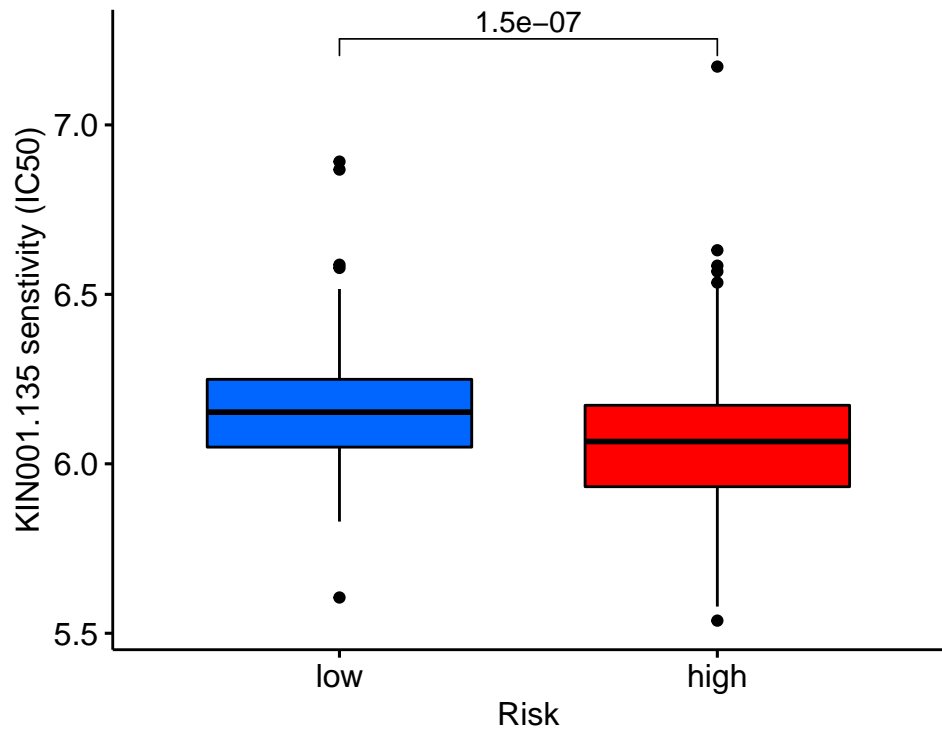

Supplement: Supplementary file 2 [file Presentation1.zip › durgSenstivity.KIN001.135.pdf]

Risk 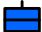 low 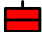 high

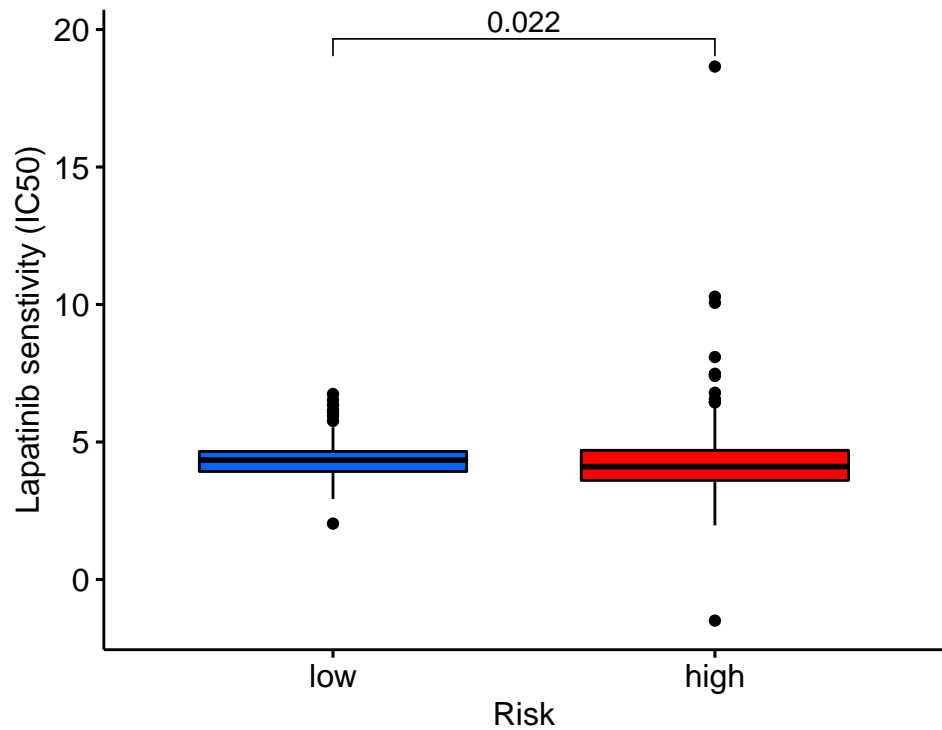

Supplement: Supplementary file 2 [file Presentation1.zip › durgSenstivity.Lapatinib.pdf]

Risk 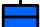 low 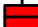 high

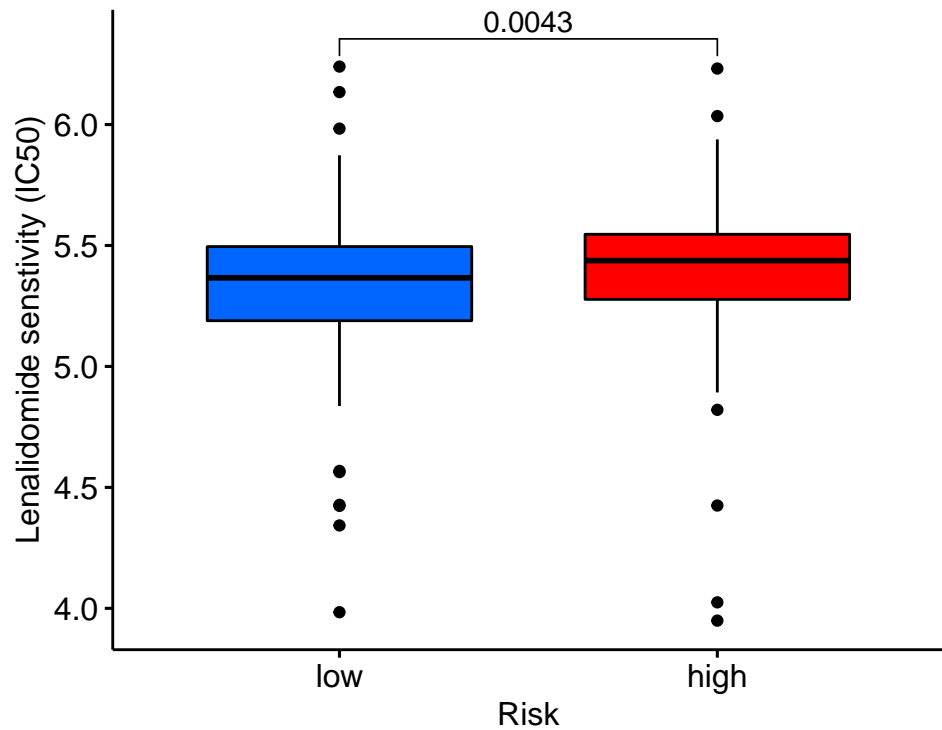

Supplement: Supplementary file 2 [file Presentation1.zip › durgSenstivity.Lenalidomide.pdf]

Risk 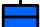 low 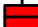 high

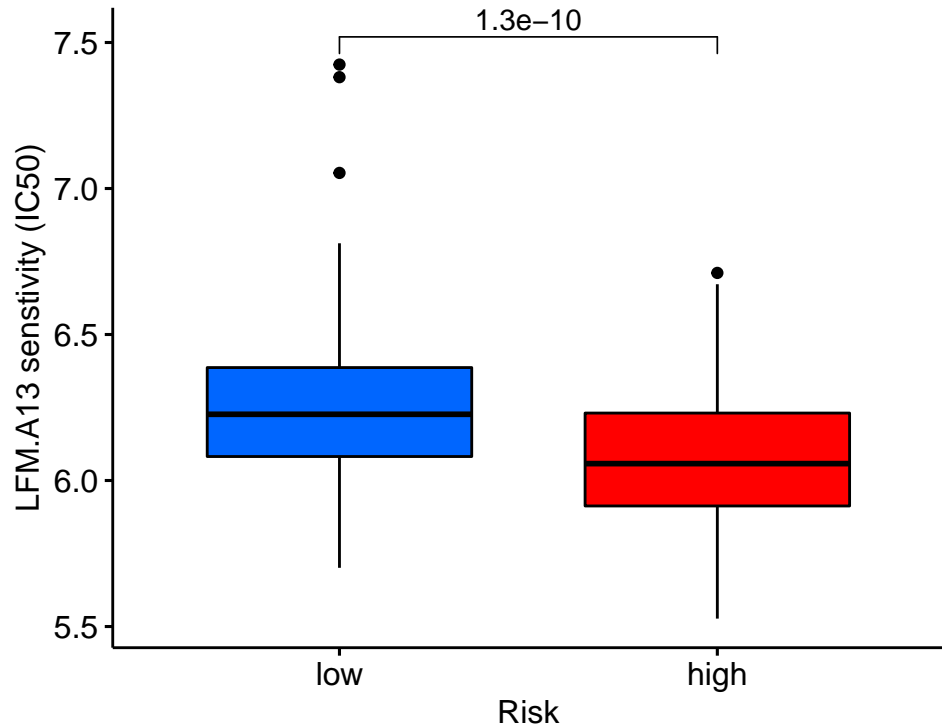

Supplement: Supplementary file 2 [file Presentation1.zip › durgSenstivity.LFM.A13.pdf]

Metformin sensitivity (IC50)

Risk 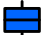 low 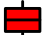 high

0.012

low

high

Risk

10

5

Supplement: Supplementary file 2 [file Presentation1.zip › durgSenstivity.Metformin.pdf]

Risk 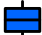 low 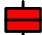 high

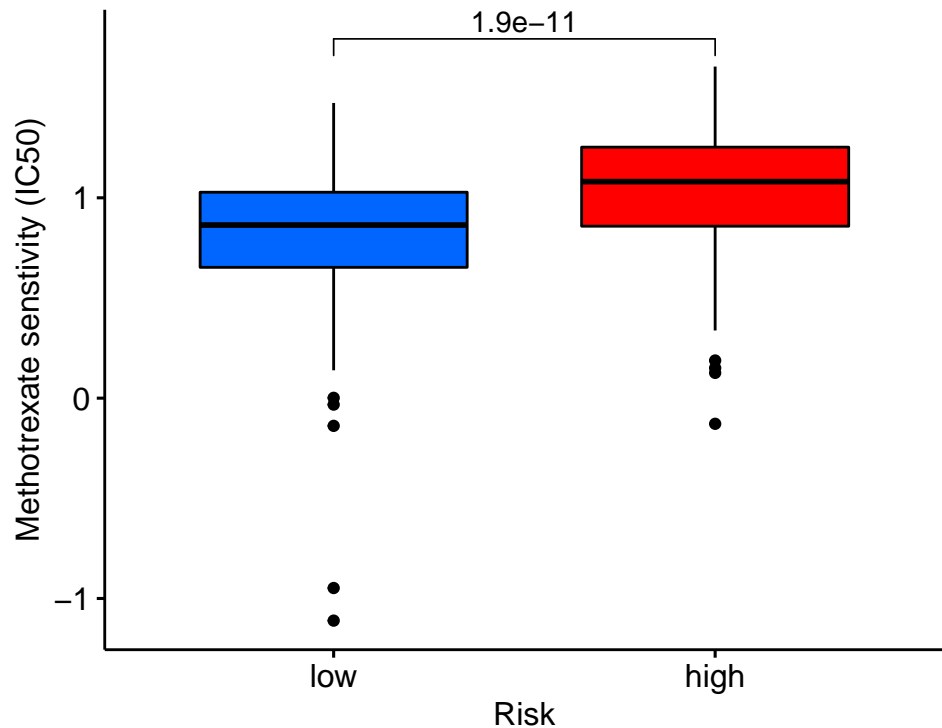

Supplement: Supplementary file 2 [file Presentation1.zip › durgSenstivity.Methotrexate.pdf]

MG.132 sensitivity (IC50)

Risk low high

0.0021

low

high

Risk

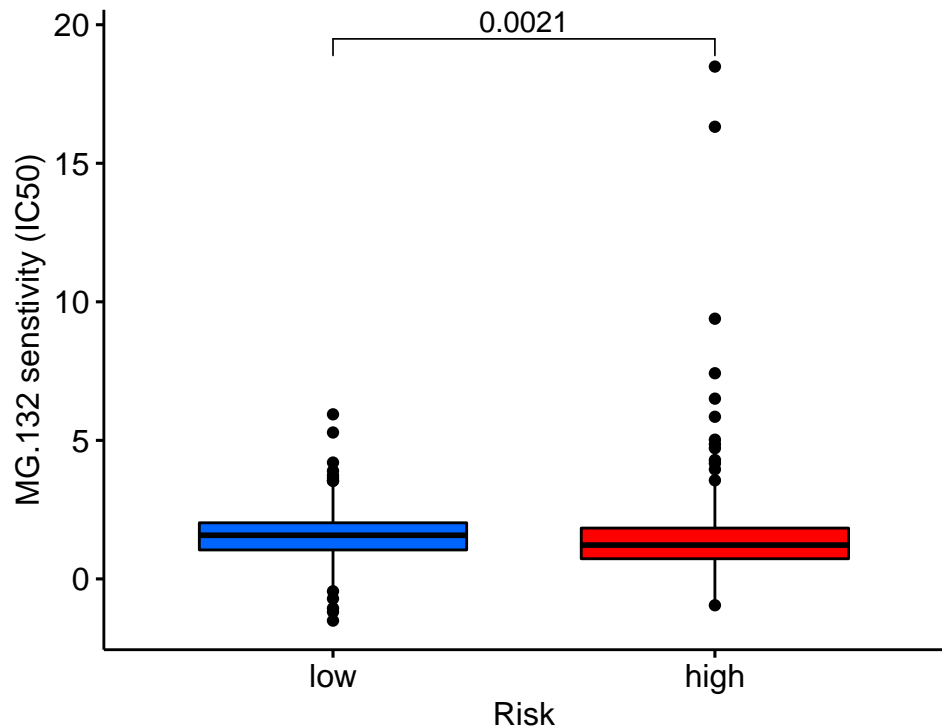

Supplement: Supplementary file 2 [file Presentation1.zip › durgSenstivity.MG.132.pdf]

Risk 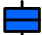 low 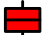 high

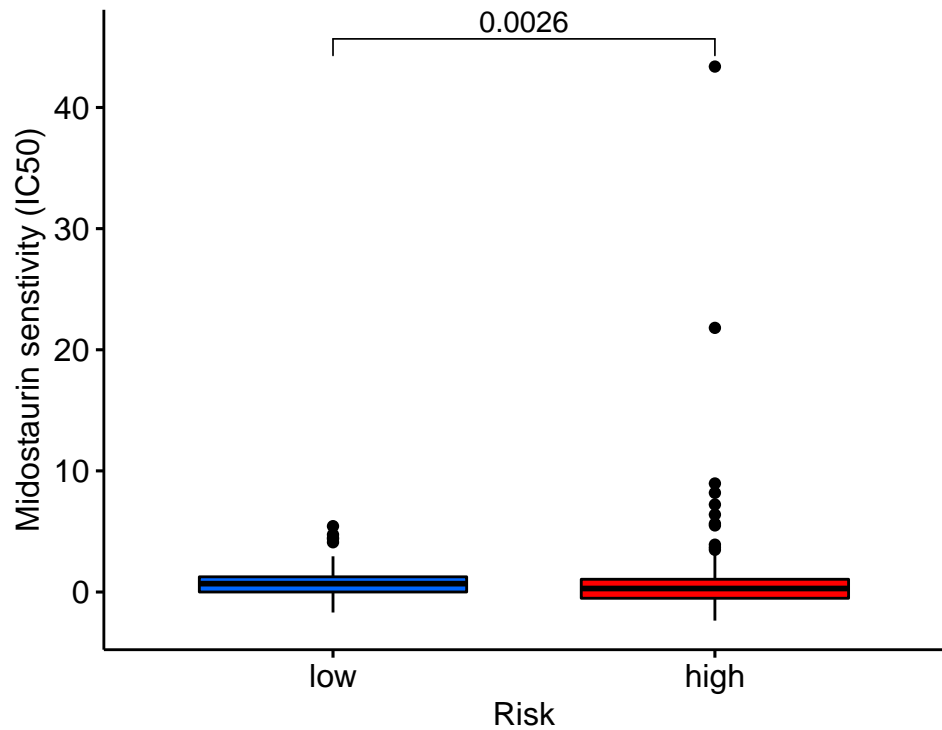

Supplement: Supplementary file 2 [file Presentation1.zip › durgSenstivity.Midostaurin.pdf]

Risk 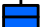 low 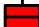 high

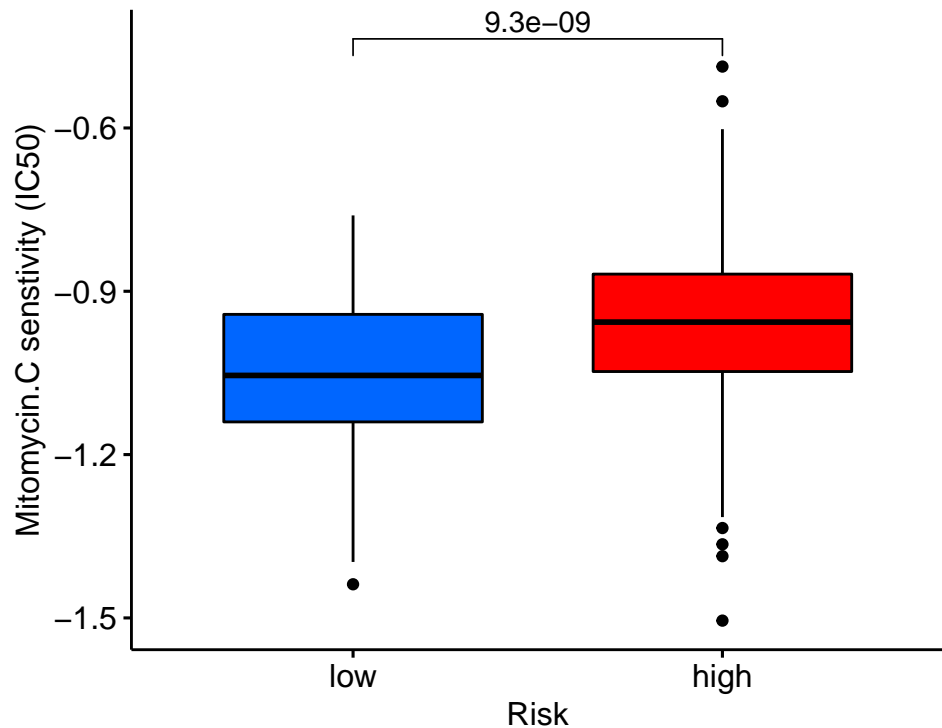

Supplement: Supplementary file 2 [file Presentation1.zip › durgSenstivity.Mitomycin.C.pdf]

Risk 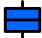 low 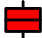 high

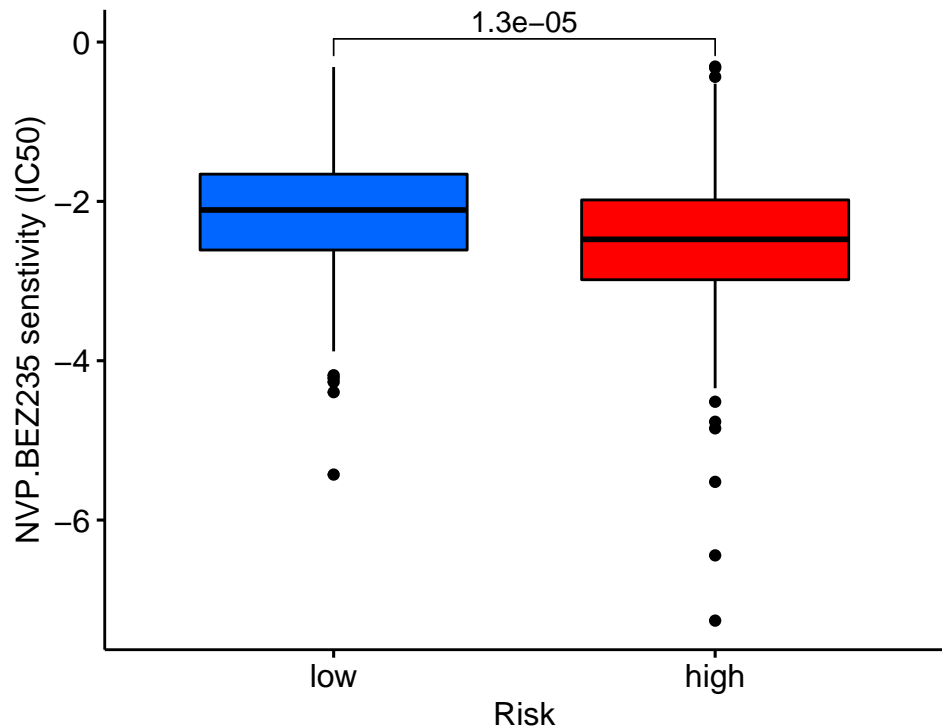

Supplement: Supplementary file 2 [file Presentation1.zip › durgSenstivity.NVP.BEZ235.pdf]

Risk 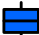 low 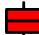 high

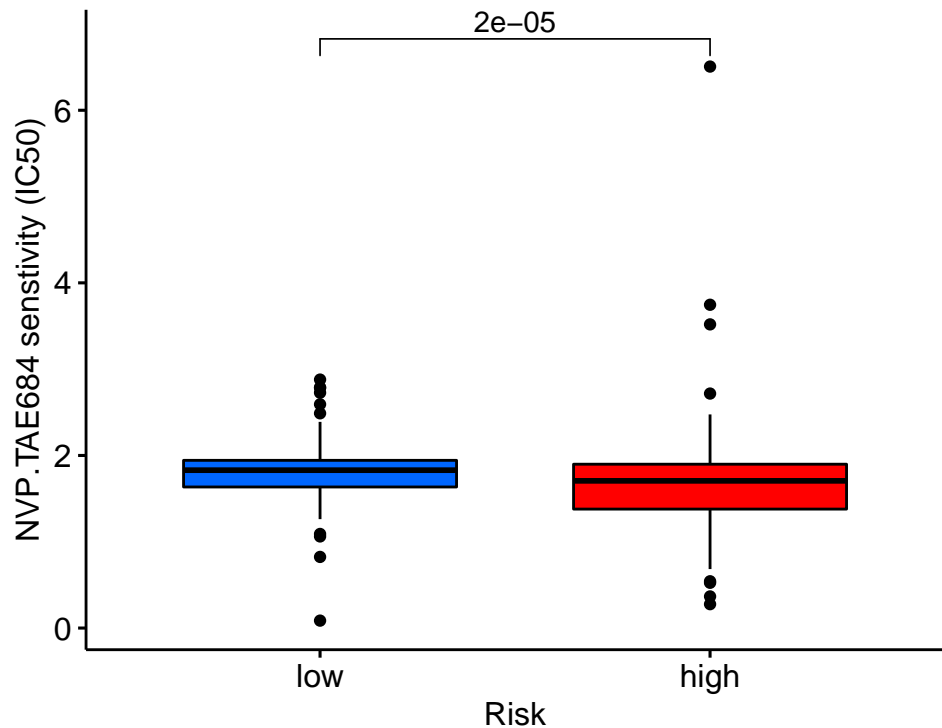

Supplement: Supplementary file 2 [file Presentation1.zip › durgSenstivity.NVP.TAE684.pdf]

Risk 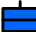 low 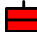 high

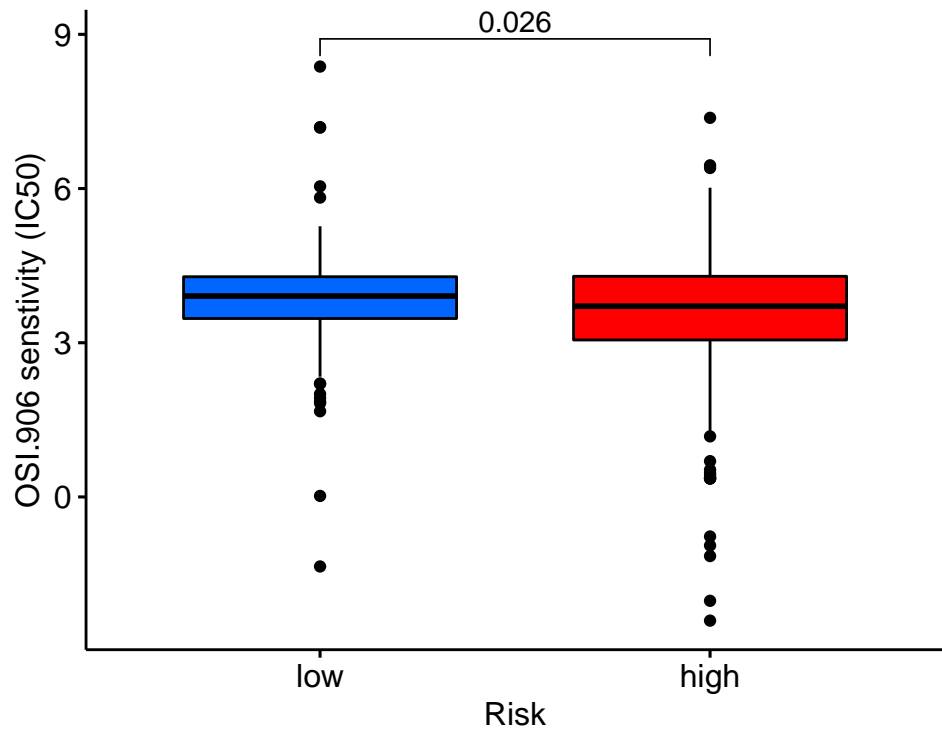

Supplement: Supplementary file 2 [file Presentation1.zip › durgSenstivity.OSI.906.pdf]
