# Supplementary material for: Prediction of Prognosis in Patients With Endometrial Carcinoma and Immune Microenvironment Estimation Based on Ferroptosis-Related Genes
Source: Front Mol Biosci. 2022 Jul 15;9:916689. doi: 10.3389/fmolb.2022.916689 (PMC9334791; doi:10.3389/fmolb.2022.916689)
Supplement: Supplementary file 3 [file Table2.DOCX]

**Table S2.** The details of the statistical values for the 6 FRGs expression across normal samples and cancer samples in TCGA database.

| Symbol | Normal samples | Cancer samples | P-value | FDR |
| --- | --- | --- | --- | --- |
| TP53 | 24.62251571 | 40.12758211 | 2.40E-08 | 5.56E-08 |
| AIFM2 | 3.189376429 | 7.125641099 | 1.03E-11 | 3.68E-11 |
| ATG7 | 2.621319114 | 3.212820421 | 0.000650472 | 0.001016362 |
| TLR4 | 3.658618643 | 1.851831548 | 9.72E-09 | 2.43E-08 |
| PANX1 | 4.8412038 | 6.002096127 | 0.030782665 | 0.038867001 |
| MDM2 | 5.207088086 | 7.196785195 | 0.009356902 | 0.012442688 |
