# Supplementary material for: Prediction of Prognosis in Patients With Endometrial Carcinoma and Immune Microenvironment Estimation Based on Ferroptosis-Related Genes
Source: Front Mol Biosci. 2022 Jul 15;9:916689. doi: 10.3389/fmolb.2022.916689 (PMC9334791; doi:10.3389/fmolb.2022.916689)
Supplement: Supplementary file 5 [file Table4.DOCX]

**Table S4.** The details of all of the ferroptosis-related genes in this signature.

| Symbol | Category | Evidence | Confidence | Source |
| --- | --- | --- | --- | --- |
| TP53 | Driver | Additionally, p53 knockdown and S392A mutant both can reduce the classical ferroptotic events by BRD7 knockin, including redox-active iron overload, lipid ROS accumulation, GSH depletion, and lipid peroxidation. | Validated | Zhang et al. 2020 |
|  |  | p53 promotes RT-induced ferroptosis partly through antagonizing SLC7A11 induction. | Validated | Lei et al. 2021 |
|  |  | p53 promoted SPIO-Serum-induced ferroptosis. | Validated | Zhang et al. 2021 |
| ATG7 | Driver | Knockout or knockdown limited erastin-induced ferroptosis. | Validated | Hou et al. 2016 |
| TLR4 | Driver | Knockdown inhibited ferroptosis. | Validated | Chen et al. 2019 |
|  |  | Inhibition of TLR4 prevents the activation of ferroptosis following HIBD. | Validated | Zhu et al. 2021 |
| PANX1 | Driver | Deletion protects against ferroptotic cell death. Silenced Panx1 expression significantly attenuated ferroptotic lipid peroxidation and iron accumulation induced by the ferroptosis inducer erastin. | Validated | Su et al. 2019 |
| MDM2 | Driver | MDM2 and MDMX promote ferroptosis by PPARα-mediated lipid remodeling. | Validated | Venkatesh et al. 2020 |
| AIFM2 | Suppressor | A glutathione-independent ferroptosis suppressor. Pharmacological targeting of FSP1 strongly synergizes with GPX4 inhibitors to trigger ferroptosis. | Validated | Doll et al. 2019 |
|  |  | A potent ferroptosis-resistance factor. Positively correlates with ferroptosis resistance. | Validated | Bersuker et al. 2019 |
|  |  | AIFM2 blocks ferroptosis independent of ubiquinol metabolism. | Validated | Dai et al. 2020 |

**REFERENCES**

Bersuker, K., Hendricks, J. M., Li, Z., Magtanong, L., Ford, B., Tang, P. H., et al. (2019). The CoQ oxidoreductase FSP1 acts parallel to GPX4 to inhibit ferroptosis. *Nature* 575:688-692. doi: 10.1038/s41586-019-1705-2

Chen, X., Xu, S., Zhao, C., and Liu, B. (2019). Role of TLR4/NADPH oxidase 4 pathway in promoting cell death through autophagy and ferroptosis during heart failure. *Biochem. Biophys. Res. Commun.* 516:37-43. doi: 10.1016/j.bbrc.2019.06.015

Dai, E., Zhang, W., Cong, D., Kang, R., Wang, J., and Tang, D. (2020). AIFM2 blocks ferroptosis independent of ubiquinol metabolism. *Biochem. Biophys. Res. Commun.* 523:966-971. doi: 10.1016/j.bbrc.2020.01.066

Doll, S., Freitas, F. P., Shah, R., Aldrovandi, M., da Silva, M. C., Ingold, I., et al. (2019). FSP1 is a glutathione-independent ferroptosis suppressor. *Nature* 575:693-698. doi: 10.1038/s41586-019-1707-0

Hou, W., Xie, Y., Song, X., Sun, X., Lotze, M. T., Zeh, H. J. 3rd, et al. (2016). Autophagy promotes ferroptosis by degradation of ferritin. *Autophagy* 12:1425-1428. doi: 10.1080/15548627.2016.1187366

Lei, G., Zhang, Y., Hong, T., Zhang, X., Liu, X., Mao, C., et al. (2021). Ferroptosis as a mechanism to mediate p53 function in tumor radiosensitivity. *Oncogene* 40:3533-3547. doi: 10.1038/s41388-021-01790-w

Su, L., Jiang, X., Yang, C., Zhang, J., Chen, B., Li, Y., et al. (2019). Pannexin 1 mediates ferroptosis that contributes to renal ischemia/reperfusion injury. *J. Biol. Chem.* 294:19395-19404. doi: 10.1074/jbc.RA119.010949

Venkatesh, D., O'Brien, N. A., Zandkarimi, F., Tong, D. R., Stokes, M. E., Dunn, D. E., et al. (2020). MDM2 and MDMX promote ferroptosis by PPARα-mediated lipid remodeling. *Genes Dev.* 34:526-543. doi: 10.1101/gad.334219.119

Zhang, Y., Xia, M., Zhou, Z., Hu, X., Wang, J., Zhang, M., et al. (2021). p53 Promoted Ferroptosis in Ovarian Cancer Cells Treated with Human Serum Incubated-Superparamagnetic Iron Oxides. *Int J Nanomedicine* 16:283-296. doi: 10.2147/IJN.S282489

Zhang, Z., Guo, M., Shen, M., Kong, D., Zhang, F., Shao, J., et al. (2020). The BRD7-P53-SLC25A28 axis regulates ferroptosis in hepatic stellate cells. *Redox Biol* 36:101619. doi: 10.1016/j.redox.2020.101619

Zhu, K., Zhu, X., Sun, S., Yang, W., Liu, S., Tang, Z., et al. (2021). Inhibition of TLR4 prevents hippocampal hypoxic-ischemic injury by regulating ferroptosis in neonatal rats. *Exp. Neurol.* 345:113828. doi: 10.1016/j.expneurol.2021.113828
