# Supplementary material for: Prediction of Prognosis in Patients With Endometrial Carcinoma and Immune Microenvironment Estimation Based on Ferroptosis-Related Genes
Source: Front Mol Biosci. 2022 Jul 15;9:916689. doi: 10.3389/fmolb.2022.916689 (PMC9334791; doi:10.3389/fmolb.2022.916689)
Supplement: Supplementary file 2 [file durgSenstivity.Paclitaxel.pdf › durgSenstivity.Paclitaxel.pdf]

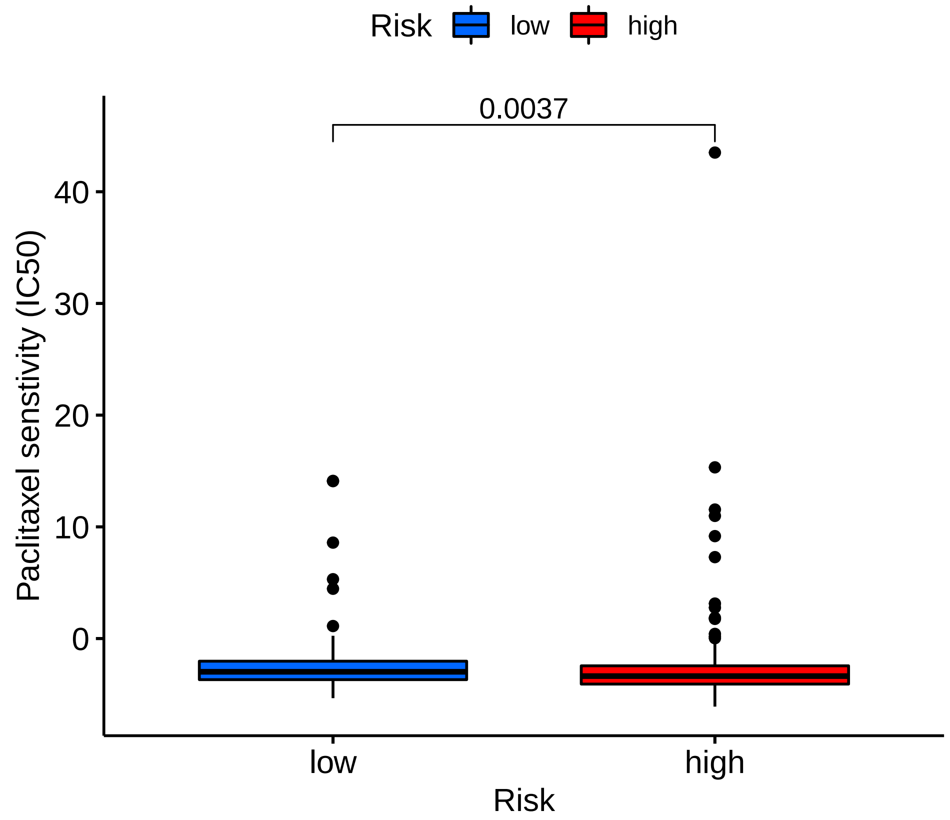

Risk
low
high
0.0037
Paclitaxel senstivity (IC50)
40
30
20
10
0
low
high
Risk
