# Supplementary material for: Prediction of Prognosis in Patients With Endometrial Carcinoma and Immune Microenvironment Estimation Based on Ferroptosis-Related Genes
Source: Front Mol Biosci. 2022 Jul 15;9:916689. doi: 10.3389/fmolb.2022.916689 (PMC9334791; doi:10.3389/fmolb.2022.916689)
Supplement: Supplementary file 2 [file durgSenstivity.Imatinib.pdf › durgSenstivity.Imatinib.pdf]

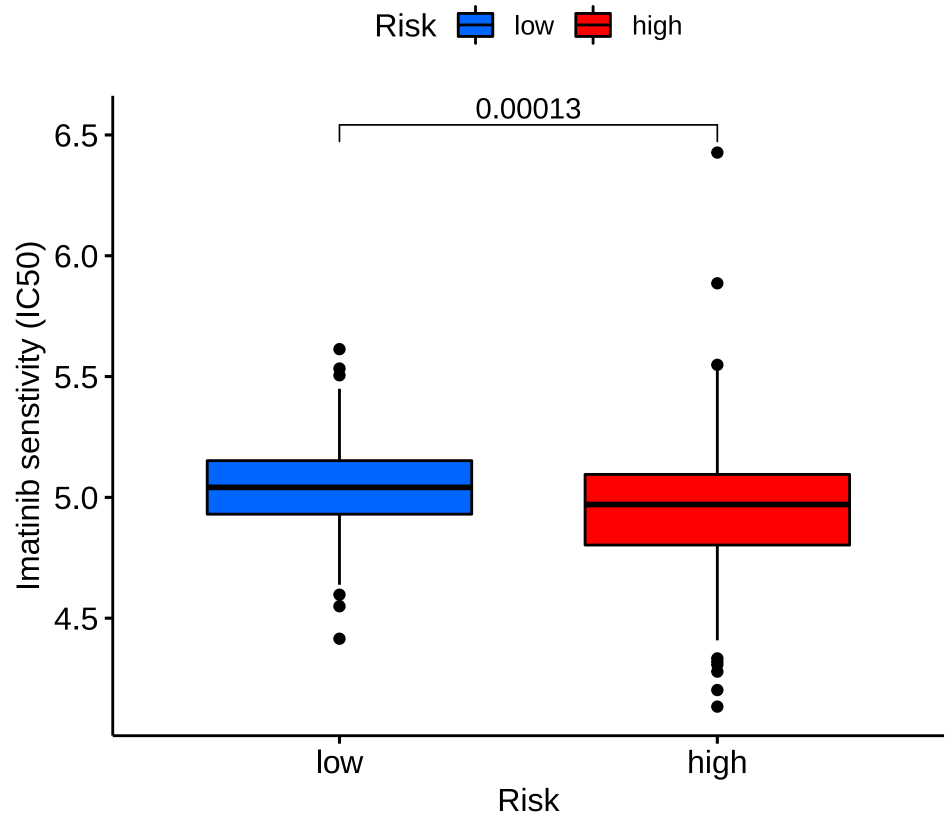

Risk
low
high
0.00013
6.5
6.0
5.5
5.0
4.5
Imatinib senstivity (IC50)
low
high
Risk
